# Dispersal can spread management benefits: Insights from a modeled Fijian coral reef network

**DOI:** 10.1002/eap.70156

**Published:** 2025-12-08

**Authors:** Ariel Greiner, Marco Andrello, Martin Krkošek, Marie‐Josée Fortin, Yashika Nand, Stacy D. Jupiter, Sangeeta Mangubhai, Amelia Wenger, Emily S. Darling

**Affiliations:** ^1^ Department of Ecology & Evolutionary Biology University of Toronto Toronto Ontario Canada; ^2^ Department of Biology Pennsylvania State University Pennsylvania USA; ^3^ Department of Biology University of Oxford Oxford UK; ^4^ Institute for the Study of Anthropic Impacts and Sustainability in the Marine Environment, National Research Council, CNR‐IAS Rome Italy; ^5^ National Biodiversity Future Center Palermo Italy; ^6^ Australian Institute of Marine Science Townsville Queensland Australia; ^7^ Wildlife Conservation Society, Fiji Country Program Suva Fiji; ^8^ Marine Program, Wildlife Conservation Society Bronx New York USA; ^9^ Wildlife Conservation Society, Melanesia Regional Program Suva Fiji; ^10^ Talanoa Consulting Suva Fiji; ^11^ Centre for Biodiversity and Conservation Science, School of the Environment, University of Queensland St. Lucia Queensland Australia

**Keywords:** alternative stable states, connectivity, conservation, coral reef, dispersal, Fiji, macroalgae, management, mathematical modeling, Melanesia, network theory

## Abstract

The combined effects of coral and macroalgal propagule dispersal, local bistability dynamics and pressures that span the land‐sea interface are not well understood, and consequently, are not well accounted for in coral reef management planning. In particular, fishing and sedimentation from nearby watersheds can tip reefs from coral‐dominated stable states to macroalgal‐dominated stable states. To address these knowledge gaps, we developed a mathematical model of the benthic cover dynamics of a 75‐Reef network in Fiji to compare the effectiveness of three different management intervention types: extending the area of periodic fishery closures to encompass more reefs (modeled by increasing herbivore grazing rates; managing a sea‐based pressure), improving water quality across Fiji (modeled by decreasing coral mortality rates; managing a land‐based pressure) and the two interventions combined (managing land and sea‐based pressures simultaneously). We ran the model with three grazing scenarios (low, medium, high) to account for uncertainty in actual herbivore grazing rates among reefs, as well as to represent regimes of macroalgal‐dominated, bistable and coral‐dominated dynamics in isolated reefs. Our results indicate that the presence of connectivity in the model stabilized the dynamics, with the final benthic cover and management effects exhibiting almost no sensitivity to initial conditions under the medium grazing scenario. The model predicts that the integrated land‐sea management is the most effective management intervention for ensuring high coral cover (>30%). We also find that fishery closure management that improves the grazing rate in less than half of the reef network can lead to increases in coral cover across the entire reef network. This result suggests that, as long as a few reefs in the network have high grazing, reefs across the network may trend to high coral cover as long as environmental conditions do not change. Based on an expected value of perfect information analysis, we find that the effectiveness of the integrated land‐sea management intervention is robust to the three grazing scenarios and suggests that this model can inform management decisions even with uncertainty. These findings advance our understanding of how a network of ecosystem patches with local bistability could behave and informs their management.

## INTRODUCTION

Globally, coral reefs are crucial to sustaining the food security and livelihoods of hundreds of millions of people (Cruz‐Trinidad et al., 2014; FAO, 2012; Sing Wong et al., 2022) and for maintaining marine biodiversity. Coral reefs are facing multiple climate stressors, resulting in coral mortality from bleaching and extreme weather events and slowed growth from ocean acidification (Hughes et al., 2018; Kroeker et al., 2010; Pandolfi et al., 2011; Sully et al., 2019). Many reefs also experience concurrent human impacts from across the land‐sea interface, including overfishing, tourism, and water pollution (including sedimentation and nutrification) (Andrello et al., 2022; Burke et al., 2011; Smith et al., 2016; Wenger et al., 2016). Coral reefs thus present a valuable opportunity to explore how best to manage land‐based and sea‐based pressures concurrently using an integrated land‐sea management approach (Alvarez‐Romero et al., 2011; Delevaux, Whittier, et al., 2018; Gilby et al., 2016). Fishing lowers fish biomass on reefs worldwide (Srinivasan et al., 2010), leading to lower grazing rates, which can result in lower coral cover through a reduction in grazers consuming algae that compete with corals for space and inhibit coral recruitment (Arnold et al., 2010; Bonaldo & Hay, 2014; McCook et al., 2001; Tanner, 1995). Various fishing regulatory protocols have been implemented on reefs worldwide to increase the sustainability of fishing activities, including periodic fishery closures that only allow fishing at certain intervals (either at certain times throughout the year or in certain years) and gear restriction or species‐restricted fishing (Cohen & Foale, 2013). Increased sedimentation levels on reefs due to erosion caused by coastal development (urban/tourism development, increased agriculture, etc.) have also been shown to increase coral mortality levels and reduce grazing rates, leading to lower coral cover and increased algal cover (Cortés & Risk, 1985; Goatley et al., 2016; Mallela et al., 2004; Nugues & Roberts, 2003; Wenger et al., 2020). To reduce sedimentation and pollution levels, managers may seek to restrict coastal development/farming and/or improve wastewater treatment (Wakwella et al., 2022; Wenger et al., 2017), but it is unclear how to manage these interacting stressors to best maintain high coral cover. Taken all together, these stressors reduce coral cover and favor other organisms (such as macroalgae and turf algae) that compete for space with coral and thus potentially reduce the stability of the coral‐dominated stable state (and by doing so, push the system to trend toward a non‐coral‐dominated stable state).

Coral reef multi‐stability has been discussed for many years (Knowlton, 1992), but was first formalized by Mumby et al. (2007). Mumby et al. (2007) used a mathematical model to determine that modification of grazing rate (e.g., by changes in fishing pressure) can transition the system from a single macroalgal‐dominated stable state at low‐grazing levels, through a bistable system where both a coral‐dominated stable state and a macroalgal‐dominated stable state coexist at medium grazing, and lastly to a single coral‐dominated stable state at high grazing. When an ecosystem has multiple stable states, it means that at that parameter combination (e.g., a certain grazing rate, mortality rate, overgrowth rate, etc.) the ecosystem can trend toward any one of the stable states, and which one it trends toward can depend on its historical state (hysteresis). While some have questioned the multiple stability paradigm in coral reefs, arguing against the existence of unstable coral–macroalgal states (i.e., that coral‐dominated and macroalgal‐dominated states exist along a stable continuum; Bruno et al., 2009; Żychaluk et al., 2012), the existence of grazing‐associated multiple stability of coral reefs has been shown through experiments and surveys of coral cover, macroalgal cover, herbivore abundance, and grazing rates in Indo‐Pacific and Caribbean reef systems (Cheal et al., 2010; Done, 1992; Hughes, 1994; Nyström et al., 2000; Rogers & Miller, 2006; Schmitt et al., 2019). Coral reef multi‐stability has also been discussed in the context of sedimentation and nutrification. For example, Fung et al. (2011) modeled predicted ecological responses to increased sedimentation and nutrification in coral reefs, and they found that these resulted in continuous or discontinuous phase shifts to lower coral cover states. Multiple stability associated with parameters affected by stressors that span the land‐sea interface (i.e., grazing‐associated and nutrification‐associated multiple stability) has been demonstrated through evidence of diverging trajectories to either a coral or a macroalgal‐dominated state on Indo‐Pacific reefs, depending on the abundance of herbivorous fish and sediment levels (among other factors, Graham et al., 2015). Determining whether coral reef multiple stable state dynamics are relevant to consider in a management context is crucial for the design of land‐sea management protocols, because ignoring these dynamics when they are present could hurt the effectiveness of any land‐sea management protocols (Suding & Hobbs, 2009). This is because such protocols would not account for the existence of stable state thresholds, potentially resulting in a managed healthy reef slowly tipping into a low coral cover stable state because an environmental parameter, like grazing rate, was allowed to fall below such a threshold.

Coral reef dynamics are also affected by the dispersal of coral larvae and macroalgal gametes. Such dispersal creates reef networks: networks defined as the set of reefs that can send or receive larvae to/from each other. Reef networks around the world vary in size (Greiner, Andrello, et al., 2022; Holstein et al., 2014), but many are likely large because coral larvae travel (fairly passively) along dominant currents and have long (>100 days) pelagic larval durations (PLDs; Gamoyo et al., 2019; Romero‐Torres et al., 2018; Wood et al., 2014). In general, dispersal is known to be a major determinant of coral reef community composition and functioning (Jones et al., 2009; Paris‐Limouzy, 2011; Veron, 1995). Studies have looked at the general relationship between coral cover and dispersal in real reef networks and found that dispersal can impact the ability of coral reefs to withstand climate change (McManus et al., 2021) and informs recommendations for marine reserve placement (Beyer et al., 2018; Hock et al., 2017; Kininmonth et al., 2011). However, many of these studies assume that more immigration is beneficial without considering that the ecosystem could have multiple stable states (e.g., does not consider that high dispersal could tip the reef into a macroalgal‐dominated stable state, see Greiner, Darling, et al., 2022). Consideration of dispersal within models that include the potential for multiple stable state dynamics provides new opportunities to focus management on important source reefs (as recommended by Hock et al., 2017; Kininmonth et al., 2011) or to think about the management of the entire reef network.

The interplay between coral reef dispersal and local multiple stability is not well understood for large reef networks but is crucial to inform land–sea conservation management decisions. In particular, models that consider multiple stability can address the question of whether high coral cover is best maintained through managing both land‐ and sea‐based stressors simultaneously or one at a time to avoid the possibility of sub‐additive interactions between management interventions or wasted effort. Dispersal and multiple stability of reefs are interlinked, as the inhibition of coral recruitment by algae is thought to be one of the key positive feedback mechanisms underlying coral–macroalgal stable states on reefs (Arnold et al., 2010; Evensen et al., 2019; Hughes, 1996; Kuffner et al., 2006; McCook et al., 2001). The interplay between dispersal and local multiple stability among coral reefs has been explored using models of one and two reefs, finding that propagule dispersal can facilitate coral resilience (Baskett et al., 2010; Elmhirst et al., 2009; McManus et al., 2019). In particular, Greiner, Darling, et al. (2022) developed a two‐reef model that demonstrated the existence of mixed coral–macroalgal stable states and dispersal‐driven tipping of high‐grazing reefs to macroalgal dominance. However, as most reef networks include more than two reefs, it is crucial to understand how dispersal among many reefs impacts the stable states of larger reef networks as this may help maximize the effectiveness of network‐wide management for sustaining high coral cover.

Here, we develop a benthic cover dynamics model of 75 reefs in Fiji and assess how they respond to three types of network‐wide management initiatives proposed by local conservation practitioners: (1) spatially expanding periodic fishery closures (sea‐based management); (2) improving water quality, as simulated by reducing the coral mortality linked to reduced sedimentation influx (land‐based management); and (3) both management interventions together (integrated land‐sea management). We model each reef with a system of equations that describe how the benthic cover of each reef changes over time due to herbivorous grazing, spatial competition, natural mortality, and dispersal within and among the 75 reefs. We run the model across a range of grazing scenarios to account for uncertainty in herbivorous grazing rates and the known importance of grazing for local reef multiple stability. We then use an expected value of perfect information (EVPI) analysis (Shea et al., 2014) to determine whether resolving the grazing rate uncertainty would improve our ability to assess the performance of management interventions. We also perform sensitivity analyses to determine whether our results are sensitive to the initial conditions chosen and to explore whether the performance of each management intervention, and our ability to determine the best management intervention, are sensitive to the parameter values. This study finds that dispersal amplifies the effects of management interventions across a reef network and shows that the choice of best management intervention is robust to grazing rate uncertainty and not sensitive to the parameter values or initial conditions. This work informs coral reef management in Fiji and improves our knowledge of how large networks of multiple stable state marine ecosystems behave.

## METHODS

### Overview

We designed and parameterized a model of 75 Fijian reef sites (“75‐Reef Fiji model”) to assess whether various management interventions increase the coral cover of the reef network, when multiple stability of each reef and dispersal among reefs are explicitly considered. We define a reef network as a set of reefs that are connected by propagule dispersal (>0 modeled dispersal probability) directly and indirectly and do not send or receive propagules to/from any other reefs. We selected a set of 75 Fijian reef sites for which we had sufficient benthic cover and fish abundance survey data collected between 2017 and 2020, out of the 551 total Fijian reef sites for which we had coordinates (see Appendix S1). We refer to these “reef sites” as “reefs” since we have separate benthic and fish data for each of them.

We parameterized a set of equations for each of these 75 reefs (and will refer to this as “the 75‐Reef Fiji Model”) using coral reef survey data from the Wildlife Conservation Society (WCS) of Fiji (accessed via the MERMAID dashboard; https://dashboard.datamermaid.org/), a global human pressures dataset (Andrello et al., 2022) and modeled dispersal probabilities for coral larvae and macroalgal gametes. We modeled dispersal probabilities for both by simulating propagule dispersal from 2009 to 2018 in reefs around Fiji using a Lagrangian dispersal simulator; we then translated the outputs of this simulation into coral and macroalgal connectivity matrices (see Appendix S2 for more details). From these data sources, every reef is given specific initial benthic cover values and values of the grazing, coral larval and macroalgal gamete dispersal, and coral mortality parameters (further details of the model parameterization are included in Appendix S3).

We then used the 75‐Reef Fiji model to make projections of future coral cover, given the initial coral cover states of the 75 reefs (derived from the WCS survey data). Then, we compared three management intervention types of interest to local managers: (M1) expanding periodic fishery closure sizes (which have been shown to increase live coral cover in Fiji in the past, Rasher et al., 2013), (M2) improving water quality as simulated through reduced coral mortality, and (M3) applying these two management interventions together (see Table 1) to assess how each management intervention might change the final coral cover of each reef (in the model) in comparison to the final coral cover of each reef under the baseline simulation (no additional management interventions). We compare the final benthic cover values that result from different management simulations as we are interested in whether the management interventions applied change the modeled outcome, not how these final benthic cover values differ from the initial state of each reef. We focus on the final benthic cover values derived from the model simulations because they represent the percent cover that each reef is predicted to trend to based on the 2017–2020 data (initial conditions, parameter values) and any added management intervention.

**TABLE 1:** We simulated three different types of management interventions, running simulations for each type of management intervention at a lower and a higher magnitude and across three different grazing scenarios: low, *g*_median_ = 0.1; medium, *g*_median_ = 0.3; high, *g*_median_ = 0.5.

Management intervention	Description
Type	
M1. Expand fishery closures	All reefs newly included in each fishery closure given the same grazing rate ($g_{i}$) as the reef originally in the fishery closure.
M2. Improve water quality	Reduce coral mortality ($d_{i}$) for all reefs.
M3. Combination	Expand fishery closures + improve water quality
Intervention magnitude	
Lower	
M1‐2 km	Increase size of fishery closures to ~2 km, only reefs within the same fishing ground included.
M2‐10%	Coral mortality (*d*_*i*_) in all reefs decreased by 10%.
M3‐2 km + 10%	M1‐2 km + M2‐10%
Higher	
M1‐5 km	Increase size of fishery closures to ~5 km.
M2‐25%	Coral mortality (*d*_*i*_) in all reefs decreased by 25%.
M3‐2 km + 25%	M1‐2 km + M2‐25%

*Note*: Three baseline simulations were also run (one at each grazing scenario), where base values of $g_{i}$ (determined by the relevant grazing scenario) and $d_{i}$ were used for all reefs. Base values of $g_{i}$ and $d_{i}$ under each management intervention were derived from WCS data or Andrello et al. (2022) data as described in Appendix S3, these base values are then modified under Management Interventions M1, M2, and M3 in the method described in this table.

We ran the 75‐Reef Fiji model under three different grazing scenarios (low, medium, and high [see Table 1]; different median grazing rate values for the entire network) to account for the uncertainty in the true herbivorous fish grazing rates in the modeled reefs (i.e., missing consumption rates). These median grazing values were chosen because in single‐reef (Elmhirst et al., 2009; Mumby et al., 2007) and two‐reef versions of this model (Greiner, Darling, et al., 2022), they correspond with three key regions of parameter space: coral dominance at high grazing (high percent cover of coral), bistability at medium grazing, and macroalgal dominance at low grazing (see Table 1). We also assess whether having perfect knowledge of the grazing scenario would improve our ability to choose a management intervention using the EVPI (Shea et al., 2014). We focus this EVPI analysis on the three grazing scenarios analyzed because: (1) we did not have the data to parameterize the grazing rates of the reefs in the model precisely and (2) past models (such as Blackwood et al., 2012; Greiner, Darling, et al., 2022; Mumby et al., 2007, etc.) tell us that it is an important control parameter in the system and one that fishery managers can modify. Lastly, we performed sensitivity analyses to assess how sensitive our findings were to the parameter and initial condition values used in the model. All the simulations and analyses were performed in R (v3.5.3, R Core Team, 2019) using the igraph (Csárdi et al., 2023), deSolve (Soetaert et al., 2010), sf (Pebesma, 2018) and ggplot2 (Wickham, 2016) packages.

### Study system

Many Fijian reefs have avoided or recovered strongly from past heat stress events between 2000 and 2020, though they have been chronically affected by high levels of pollution and overfishing (Mangubhai et al., 2019). Recent intense storms, such as Tropical Cyclone Winston in 2016, had a variable impact on coral cover and benthic structure, with differential subsequent recovery (Mangubhai, 2016; Mangubhai et al., 2019; Price et al., 2021). Indigenous (*iTaukei*) Fijians have use and access rights to their customary fishing grounds (qoliqoli); thus, all reefs within these fishing grounds could be considered under limited management through access restrictions. Many communities in Fiji, and across the broader Western Pacific, have additionally created self‐imposed rules that apply within locally managed marine areas (LMMAs) within their fishing grounds: these rules can include measures such as no‐take areas, species restrictions, gear restrictions, catch limits, and access restrictions (Jupiter et al., 2014). Periodically harvested fishery closures are the most common form of management within Fiji LMMAs and refer to a no‐take area (locally called a *tabu*) that may be opened and harvested for variable periods (Cohen & Foale, 2013). The same Indigenous rightsholders who can establish LMMA rules also hold tenure over adjacent lands (though sometimes the same areas are shared between multiple groups), and as such can make decisions about protective and restorative land‐based actions to reduce sedimentation and improve downstream water quality (Clarke & Jupiter, 2010; Jupiter et al., 2024). Thus, the management interventions tested in the 75‐Reef Fiji model represent realistic types of management action for Fijian coral reefs.

### Model

The 75‐Reef Fiji model is based on the models of Mumby et al. (2007), Elmhirst et al. (2009), and extends the two‐reef model of Greiner, Darling, et al. (2022) to model a multi‐reef system. We modify the equations from Greiner, Darling, et al. (2022) following McManus et al. (2019) (see Fung et al., 2011 and Blackwood et al., 2018 for more examples) to consider three state variables: coral ($C_{i}$), macroalgae ($M_{i}$), and free space ($F_{i}$). Here, “free space” represents the cover of other space‐filling benthic organisms on reefs that can be settled by coral and macroalgal recruits (i.e., turf algae, crustose coralline algae, etc.) (Sheppard et al., 2019). The 75‐Reef Fiji model equations that control the benthic cover dynamics of each reef i (i: {1–75}) are as follows:

(1) $$\frac{dM_{i}}{\mathrm{dt}}=aM_{i}C_{i}-\frac{g_{i}M_{i}}{M_{i}+F_{i}}+\sum_{j}\gamma n_{\mathrm{ij}}F_{i}M_{j},$$

(2) $$\frac{dC_{i}}{\mathrm{dt}}=\sum_{j}rk_{\mathrm{ij}}F_{i}C_{j}-aM_{i}C_{i}-d_{i}C_{i},$$

(3) $$1=M_{i}+C_{i}+F_{i}.$$

Equation (1) describes how the proportion of macroalgae cover in reef i ($M_{i}$) changes over time. The macroalgae cover increases due to mature macroalgae overgrowing mature coral at a rate a, and as gametes from reef j (j could be the focal reef (reef i) or any of the other 74 reefs in this 75‐Reef Fiji model) settle on free space ($F_{i}$) in reef i at a rate $n_{\mathrm{ij}}$ at a gamete production rate of γ ($\sum_{j}\gamma n_{\mathrm{ij}}F_{i}M_{j}$). The macroalgae cover decreases due to indiscriminate grazing by herbivores at a rate $g_{i}$ on macroalgae and free space, as modeled by ($g_{i}✕\frac{M_{i}}{M_{i}+f_{i}}$). Equation (2) describes how the proportion of coral cover in reef i ($C_{i}$) changes over time. $C_{i}$ increases due to coral larvae from reef j (j could be the focal reef (reef i) or any of the other 74 reefs in this 75‐Reef Fiji model) settling on free space ($F_{i}$) in reef i at a rate $k_{\mathrm{ij}}$ at a larval production rate of *r* ($\sum_{j}rk_{\mathrm{ij}}F_{i}C_{j}$). The coral cover decreases due to mature macroalgae overgrowing mature coral at a rate a and due to coral mortality at the rate $d_{i}$. Equation (3) implicitly gives the dynamics of free space ($F_{i}$). For each simulation, we ran the model for 20,000 time steps, which was more than sufficient for all of the state variables to stabilize. Note that the gamete production rate (γ), larval production rate (r) and the rate that macroalgae overgrow mature corals (a) are the same for every reef; for more details see Appendix S3.

**FIGURE 1:**
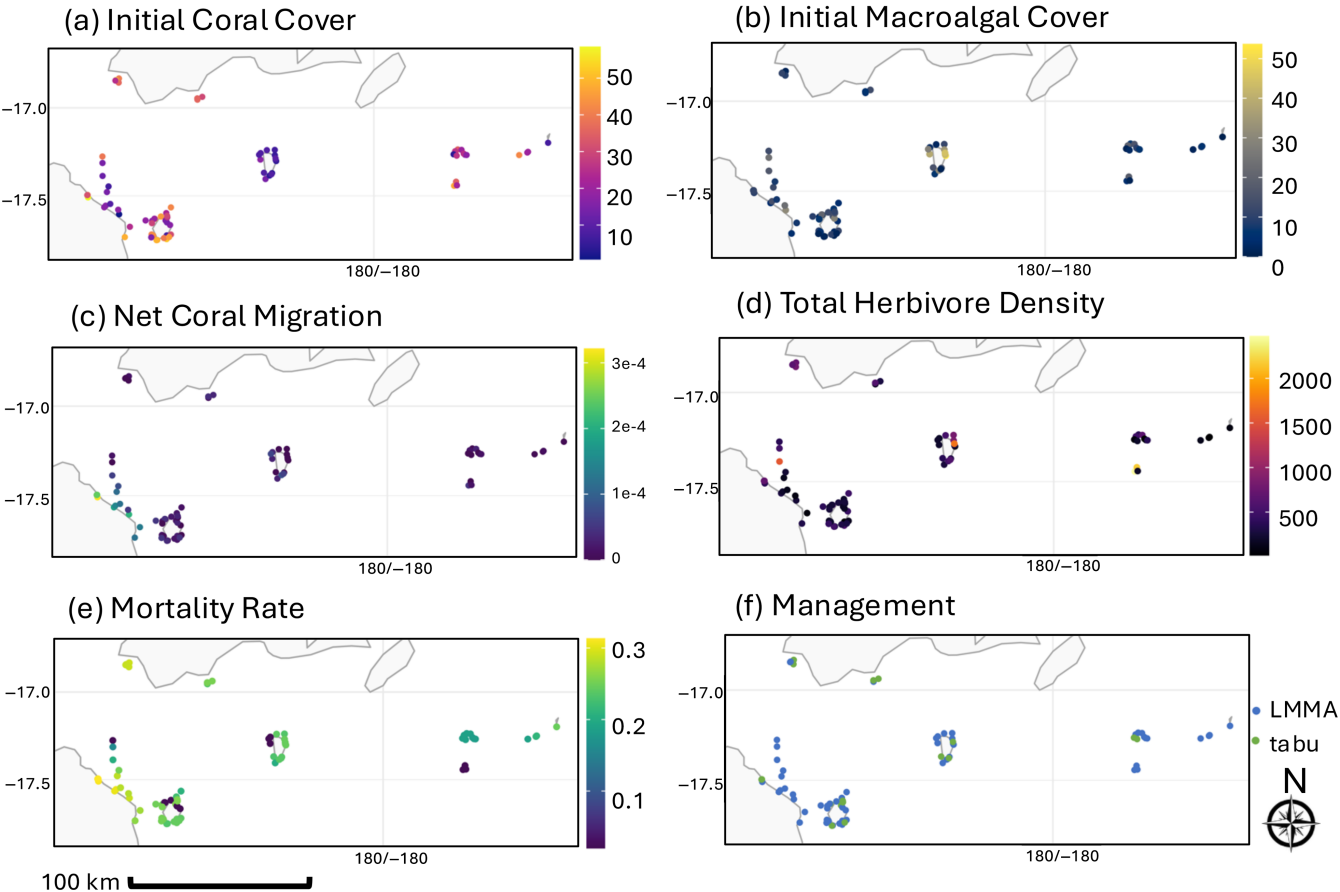
Model parameterization maps—(a) Initial coral cover: Derived from benthic point intercept transect surveys performed by WCS for each of the 75 reefs between 2017 and 2020. These values correspond to the initial values of $C_{i}$ in the 75‐Reef Fiji Model. (b) Initial macroalgal cover: Derived from benthic point intercept transect surveys performed by WCS for each of the 75 reefs between 2017 and 2020. These values correspond to the initial values of $M_{i}$ in the 75‐Reef Fiji Model. (c) Net coral migration: The rate at which coral larvae immigrate from reef j to reef i ($k_{\mathrm{ij}}$) was calculated by generating a coral larval connectivity matrix from a hydrodynamic model for Fiji. In this panel, we show the net proportion of coral migrating to each reef i (immigration‐emigration). (d) Total herbivore density: Density (kg/ha) of detritivore herbivores and macroalgal herbivores (as defined in the MERMAID dashboard, https://dashboard.datamermaid.org/) from fish belt surveys performed by WCS between 2017 and 2020. These values are then transformed into grazing rate values for each of the 75 reefs ($g_{i}$) by multiplication with a consumption rate term (see Appendix S3). (e) Mortality rate: The coral mortality rates for the 75 reefs ($d_{i}$) were derived from sedimentation levels from Andrello et al. (2022) (by rescaling them according to mortality rates used in previous models—see Appendix S3 for more details) as computed for grid cells (5 km × 5 km) in this region. (f) Management levels: Current management levels as defined by local experts at WCS for each of these reefs (LMMA = locally managed marine area, tabu = periodic fishery closures). Gray areas in maps indicate land. Note that the rate at which mature macroalgae overgrow mature coral (a) was set at 0.1 following from previous modeling studies (Elmhirst et al., 2009; Greiner, Darling, et al., 2022; Mumby et al., 2007) in the absence of empirical information on this parameter from Fiji. More details on how all these parameter values were calculated is in Appendix S3.

We model the dynamics of the 75 reefs that had sufficient survey data available to initialize Equations ((1), (2), (3))–((1), (2), (3)) (i.e., the 75‐Reef Fiji model). Modeling 75 reefs allowed us to have a reasonably sized reef network (all reefs in the same highly connected network, show characteristics of a scale‐free network (Lewis, 2011); see Appendix S4: Table S7 for more details) that was also small enough to allow us to understand the mechanisms underlying the results. We only used the benthic (coral and macroalgal) cover and fish density data between 2017 and 2020 to represent the conditions after the Category 5 tropical cyclone Winston. For each reef, we only used data from the most recent surveyed year with both fish and benthic cover data, so the initial values of $C_{i}$, $M_{i}$ and $F_{i}$ for each reef come from only 1 year in the 2017–2020 range and thus may depict reefs at different points along their post‐cyclone Winston trajectories (for more details on model parameterization, see Appendix S3). These 75 reefs are clustered in the Vatu‐i‐Ra Seascape (in comparison to the general distribution of Fijian reefs, see Appendix S1), a region of particular conservation interest due to its high biodiversity (over 300 species of coral) and endemic and endangered species (Jupiter et al., 2012). The 75 reefs are affected by variable sedimentation loads and management rules, and support different herbivorous fish densities (Figure 1). The 75 reefs included in this study varied in exposure level (exposed, semi‐exposed, sheltered), reef type (fringing, patch, lagoon, and barrier reefs), and reef zone (backreefs and forereefs), and the survey data from these reefs varied in depth from 6 m to 10 m.

### Management interventions

We explored a baseline simulation with no additional management as well as simulations with additional management in the form of one of three management intervention types (M1, M2, and M3; Table 1): expanding the size of all the periodic fishery closures (*tabu*, hereafter referred to as a “fishery closure” or “closure”) areas (M1, Table 1); improving the water quality across all the reefs (specifically, simulating reducing sedimentation levels via reducing the coral mortality across all the reefs; M2, Table 1); and performing both management interventions concurrently (M3, Table 1). We ran two different fishery closure‐based interventions: (1) increasing the area of the fishery closure to include all other reefs in the same fishing ground within a ~ 2‐km radius of the reef originally in a fishery closure (increased from ~0.5‐km radius in most cases; M1‐2 km in Tables 1 and 2) increasing the area of the fishery closure to include all reefs within a ~5‐km radius (regardless of whether or not they are in the same fishing ground) (M1‐5 km in Table 1) (details in Appendix S5). M1‐5 km is considered a less realistic management intervention and is included for comparison of spatial effects, as a 5‐km fishery closure radius exceeds the spatial scale of most local management in Fiji (larger than some fishing grounds) and would thus necessitate collaboration among fishing ground rightsholders and would severely limit fishing access for those that rely on it. To implement these fishery closure interventions, all reefs newly included in the fishery closure (of the 75 reefs in the model) were given the same grazing rate as the reef originally in the fishery closure (or average grazing rate of those original fishery closure reefs, if any reef was in multiple fishery closures; details in Appendix S5). Twenty‐one of the 75 reefs in the model were originally in fishery closures; under M1‐2 km, 15 additional reefs were included in the newly expanded fishery closure, and under M1‐5 km, 31 additional reefs were included. Most of the time the grazing rate of the reefs in the expanded fishery closures (original reefs and newly included reefs) increased but not always: 16 reefs' grazing rates increased, 6 decreased under M1‐2 km and 31 reefs increased, 14 decreased under M1‐5 km. This is consistent with prior evidence of higher grazing rates of macroalgae in fishery closures in Fiji in the past (Bonaldo et al., 2017; Rasher et al., 2013).

**TABLE 2:** Expected value of perfect information—The numbers in the table below represent the number of reefs under each management intervention and grazing scenario whose final percent coral cover is >30%.

Grazing scenario/management intervention	Low (median = 0.1)	Medium (median = 0.3)	High (median = 0.5)	Average across scenarios
1. M1‐2 km	**1**	11	12	8
2. M1‐5 km	**1**	12	12	8.333
3. M2‐10%	0	12	14	8.667
4. M2‐25%	**1**	13	**16**	10
5. M3‐2 km + 10%	**1**	12	14	9
6. M3‐2 km + 25%	**1**	**15**	**16**	*10.667*
Average across management interventions	0.8333	12.5	14	

*Note*: The numbers in **bold** represent the highest values in each column (i.e., the management intervention that results in the highest no. reefs with a percent coral cover >30% under that grazing scenario) and the value in *italics* (10.667) is the highest average no. reefs with percent coral cover >30% across all management interventions (averaged across grazing scenarios). Taking the average of the highest value in each of the three middle columns (i.e., the numbers in **bold** (i.e., (1 + 15 + 16)/3 = 10.667)) and then subtracting the highest value in the final column (i.e., the number in *italics*: 10.667) gives the EVPI value for this analysis (0).

We explored the potential effects of improving the water quality across all of Fiji's coastal waters instead of improving the water quality at specific locations because: (1) Fiji‐wide water quality management is of interest to local managers and (2) the 75‐Reef Fiji model is not precise enough to predict the true effects of improving water quality in any particular area, as it only models a subset of all reefs in Fiji. In the same way, we focused on the effects of increasing the size of all the fishery closures in Fiji (i.e., M1) included in the 75‐Reef Fiji model and not specific fishery closures so as to explore, in general, the effects of such a management intervention. In Appendix S6, we include results from running management interventions (referred to as “scattered water quality management interventions”) that improve water quality at only the reefs newly included in fishery closures under the M1‐2 km management intervention. This “scattered water quality management intervention” is unlikely to be operational, as it is not possible to control sediment flows this precisely, but we include it so as to more directly compare the impact of the fishery closure management interventions with the water quality management interventions. We ran two water quality improvement interventions, improving the water quality by either 10% (M2‐10%, see Table 1) or 25% (M2‐25%, see Table 1) by multiplying the coral mortality ($d_{i}$) by $1-\frac{x}{100}$ (*x* = {10,25}) for all reefs. In the absence of precise information regarding the exact relationship between sedimentation levels (water quality) and coral mortality in Fiji, we assume that reducing the coral mortality by *x*% represents reducing the sedimentation level (and thus, improving the water quality) by *x*% (Erftemeijer et al., 2012; Fabricius, 2005). We focus on the well‐documented effect of sedimentation on coral mortality (Bainbridge et al., 2018; Erftemeijer et al., 2012; Fabricius, 2005; Nugues & Roberts, 2003) and do not explore any of the other more complex ways that increased sedimentation can impact coral reef ecosystems (modifying grazing rates—Gordon et al., 2016; modifying recruitment rates—Bainbridge et al., 2018; Speare et al., 2019; Tebbett et al., 2018; Tebbett & Bellwood, 2019, etc.), so it is easier to understand the effects of this type of management intervention in this model. We also explored seven other water quality improvement interventions (30%, 40%, 50%, 60%, 70%, 80%, and 90%) that are less realistic (according to expert opinion and some preliminary exploratory modeling studies; Delevaux, Jupiter, et al., 2018; Appendix S7). Lastly, we combine M1‐2 km and M2‐10% (M3‐2 km + 10%, see Table 1) and M1‐2 km and M2‐25% (M3‐2 km + 25%, see Table 1) to explore whether applying land‐ and sea‐based management interventions that were reasonable to practitioners concurrently would have super‐additive, additive or sub‐additive effects on the final benthic cover of the reef network.

To assess the effects of all these management interventions, we computed the differences in the final percent coral and macroalgal cover in each reef between each management intervention and the baseline simulation under the same grazing scenario. By looking at the change in final percent coral cover and the change in final percent macroalgal cover, we can see which reefs could be trending toward a coral‐dominated state or toward a macroalgal‐dominated state (respectively).

### Expected value of perfect information (EVPI) assessment

To explore the importance of obtaining precise estimates of grazing rates when making management intervention decisions, we used the EVPI framework (Shea et al., 2014; Shea et al., 2020). We focused on assessing how robust the management interventions are to differences in grazing scenarios. Although grazing rates are not often assessed directly during fieldwork surveys, changing the grazing rate is known to change the stability of the coral‐dominated state in models (Blackwood et al., 2012; Elmhirst et al., 2009; Fung et al., 2011; Greiner, Darling, et al., 2022; McManus et al., 2019; Mumby et al., 2007) and in field experiments/surveys (Graham et al., 2015; Schmitt et al., 2019). Hence, we wanted to assess whether more precise knowledge of the grazing rates on each reef would be necessary to make informed management decisions in the 75‐Reef Fiji model. In this analysis, the EVPI value communicates the extent to which our ability to choose a management intervention is influenced by grazing scenario uncertainty. If the value of the EVPI is small, that indicates that better knowledge of the grazing scenario would not help us make a better management intervention decision and indicates that there is a clear best management intervention that is robust to grazing scenario uncertainty. To calculate the EVPI, we assigned the number of reefs with >30% coral cover as our metric of interest and calculated the value of this metric under each of the management interventions, and then determined: (1) the largest value of this metric under each grazing scenario and (2) the average value of this metric for each management intervention, across all the grazing scenarios. The >30% cut‐off was chosen as reefs with >30% coral cover are considered “healthy” enough to maintain biodiversity and fisheries over time (Birrell et al., 2020; WCS, 2022). Then, the EVPI value is calculated by subtracting the largest of the values calculated in (2) from the average of the values calculated in (1) (i.e., “best of the averages” subtracted from the “average of the best”).

### Parameter and initial condition sensitivity analyses

To determine whether our results were robust to varying the median coral mortality rate (*d*_med_) (across all 75 reefs), the rate of overgrowth of mature macroalgae over mature coral (*a*), and the coral larval dispersal rates (*k*_*ij*_), we ran the model over various values of *d*_med_, various values of *a*, and with different underlying coral larval connectivity matrices. The values of *d*_med_ and the values of *a* were chosen to span a similar range of values used for these parameters in similar benthic cover models (Mumby et al., 2007; Elmhirst et al., 2009; Fung et al., 2011; Blackwood et al., 2012; Fabina et al., 2015; McManus et al., 2019; Greiner, Darling, et al., 2022; for more details, see Appendix S4). To construct the coral larval connectivity matrices, we varied the PLD of the coral larvae systematically from a PLD of 10 days to 130 days to reflect variation in coral PLDs in the literature (Treml et al., 2008 (15–60 days PLD); Wood et al., 2014 (1–120 days PLD); Schill et al., 2015 (30 days PLD); Treml et al., 2015 (10–60 days PLD); Hock et al., 2017 (7–14 days PLD); Romero‐Torres et al., 2018 (30–150 days PLD); Gamoyo et al., 2019 (15–60 days PLD)), and our uncertainty in coral PLDs in Fiji (note that the connectivity matrix used to generate the other results in this study was a weighted average of all of these connectivity matrices, see Appendix S2 for more details). The resulting network structure of most of these connectivity matrices was similar to that of the original weighted average PLD connectivity matrix–though the smaller PLD connectivity matrices (10 days, 15 days) resulted in reefs in multiple networks as opposed to just one and some of the graphs share characteristics with two regular networks (Lewis, 2011; Appendix S4: Table S7). We then reran the baseline and management intervention simulations described above across all the grazing scenarios under each of these values of *a*, median values of *d*, and different connectivity matrices to assess whether varying these values impacted the performance of the management interventions. We also performed an EVPI analysis across the different grazing scenarios, the values of *a*, the median values of *d* and the connectivity matrices to assess whether the effectiveness of the management interventions is robust to our uncertainties in grazing, coral mortality, rate of overgrowth of mature macroalgae over mature coral and coral larval dispersal. Since the effectiveness of the management interventions was not affected by varying any of these parameters, the results of this analysis are in Appendix S4.

To determine the sensitivity of our management results to the initial conditions derived from the empirical data, we reran the baseline and management intervention simulations described above across all of the grazing scenarios under a variety of initial condition scenarios (see Appendix S8 for further details). We chose to explore very high and very low coral cover initial conditions to assess whether we could detect an impact of the multiple stable state dynamics in the model on the management intervention performance, under the different grazing scenarios. Since varying the initial conditions only resulted in minimal changes to the final results, the results of this analysis are in Appendix S8.

## RESULTS

### Baseline simulation

**FIGURE 2:**
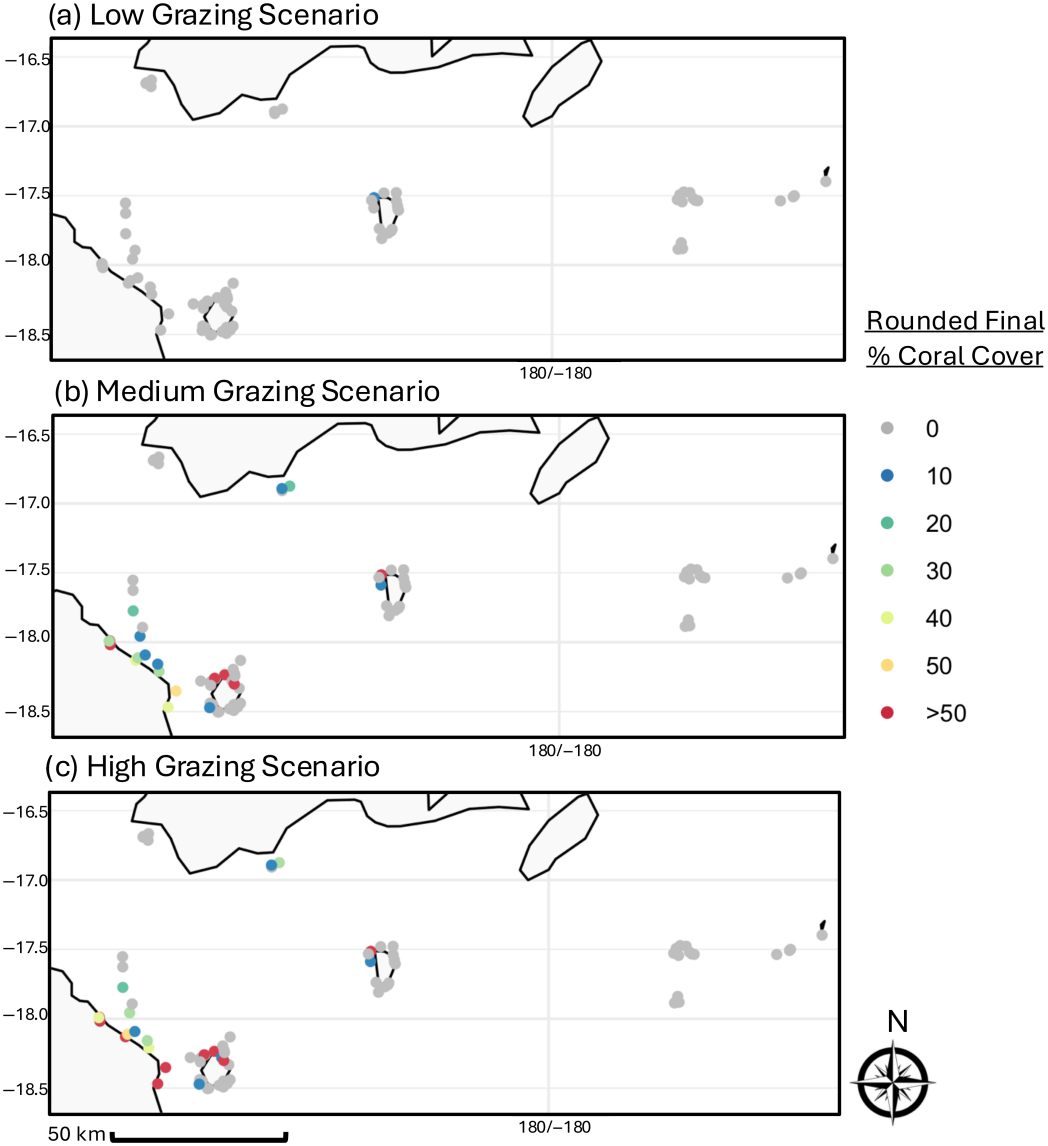
Baseline Simulation Maps**—**Final percent coral cover in all the reefs under the three grazing scenarios (a–c) without additional management interventions. Percent coral cover rounded to the nearest 10% for 0%–50% and then all values >50% are shown as simply “>50” for ease of visualization.

Under the baseline simulation, the final coral cover was less than 10% on all 75 reefs in the low‐grazing scenario and less than 10% on 60 and 56 reefs in the medium and high‐grazing scenarios, respectively. The number of reefs with higher than 30% final coral cover increased as the median grazing rate increased, with 0 reefs above 30% coral cover in the low‐grazing scenario but 10 and 12 reefs above 30% coral cover in the medium and high‐grazing scenarios, respectively (Figure 2).

### Effects of grazing, coral mortality, and coral migration on final coral cover

**FIGURE 3:**
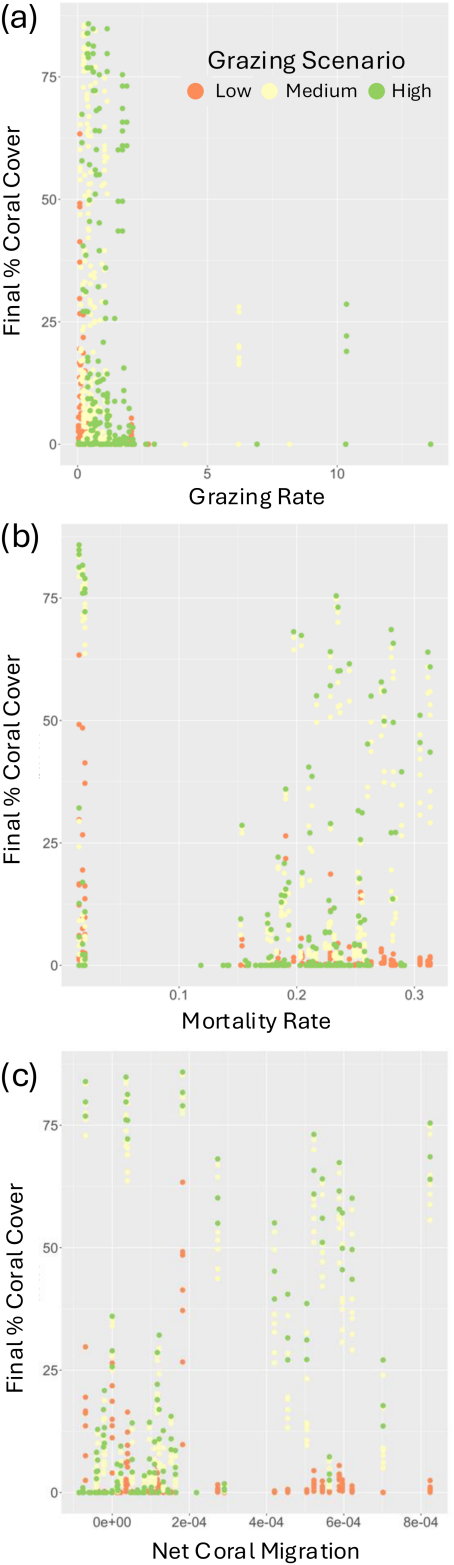
Scattergrams relating Coral Cover and Grazing Rate, Coral Cover and Mortality, and Coral Cover and Net Migration Levels According to Grazing Scenarios—Final percent coral cover plotted in relation to the grazing rate (a), mortality rate (b), and net coral migration (c) for each reef across all management interventions listed in Table 1 (baseline simulation and M1, M2, M3), colored according to grazing scenario. Each point represents the final % coral cover of a particular reef under a particular management intervention.

Coral cover did not exhibit a strong relationship with grazing rate, mortality rate, or net migration of coral. The medium and high‐grazing scenarios led to higher final coral cover than the low‐grazing scenario, but the reefs with the highest grazing rates did not have the highest final coral cover (Figure 3a). As the mortality rate increased, there was a slight decline in the highest final coral cover achieved across all reefs. Under the medium and high‐grazing scenarios, the reefs exhibited a wide range of final coral cover values at all mortality rates, but under the low‐grazing scenario, the final coral cover more noticeably declined as the mortality rate increased (Figure 3b). The relationship between net migration (the amount of larvae that enter each reef—the amount of larvae that leave each reef) and final coral cover varied by grazing scenario; under the low‐grazing scenario, final coral cover declined with increasing net migration rate; under the medium and high‐grazing scenarios, the final coral cover increased as net coral migration rate increased for some reefs and for other reefs it decreased (Figure 3c).

### Management intervention simulations

**FIGURE 4:**
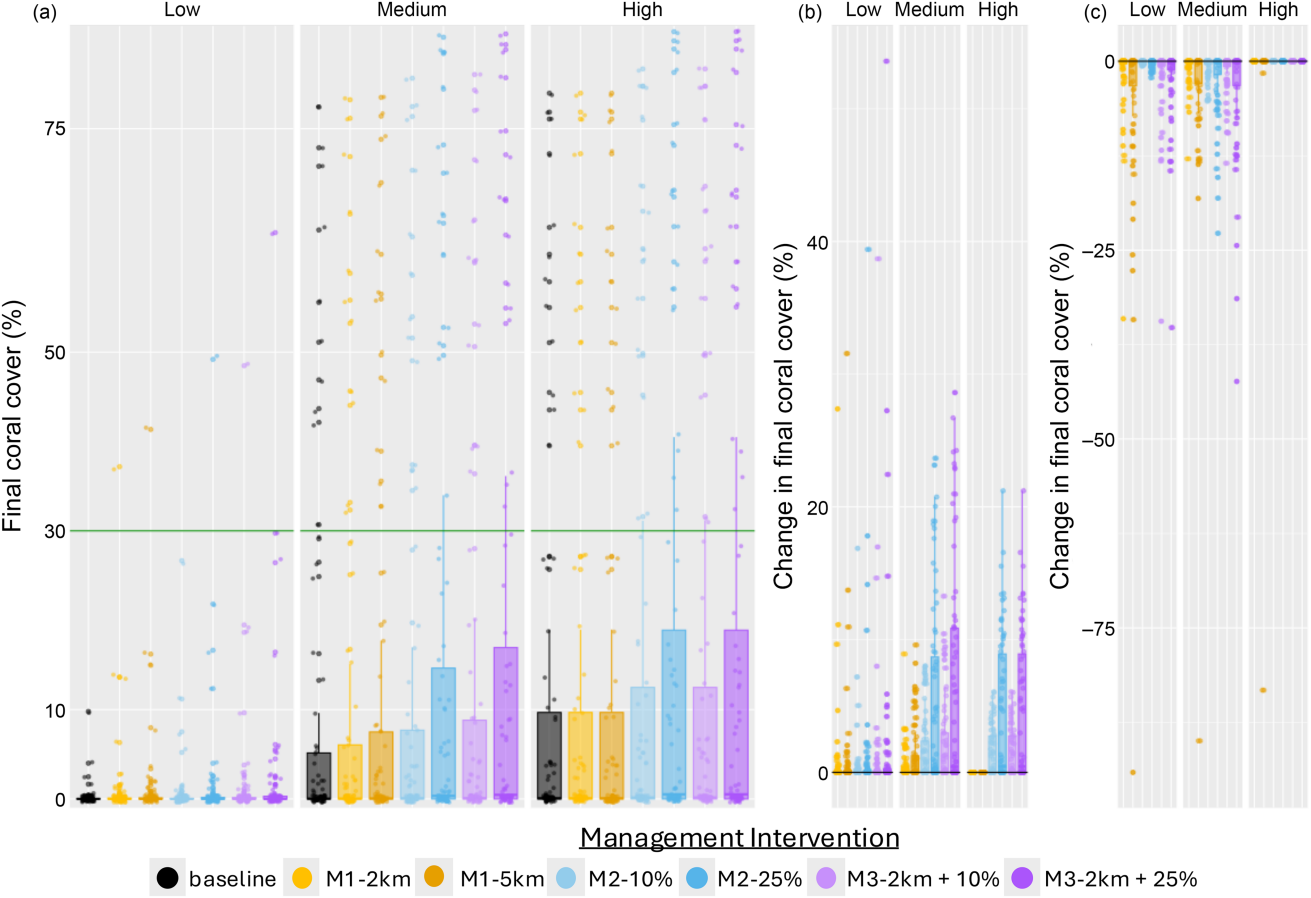
Effects of the Management Interventions—(a–c) The effect of the management intervention on the final coral cover of each reef, while each panel shows the effect of the management under each grazing scenario. (a) Final percent coral cover in each reef, with a green line at 30% indicating a healthy reef (Birrell et al., 2020; WCS, 2022). (b) Difference in the percent coral cover in each reef between each management intervention and the baseline simulation, the black line at 0 indicates the reefs that went through no change in percent coral cover. (c) Difference in the percent macroalgal cover in each reef between each management intervention and the baseline simulation, the black line at 0 indicates the reefs that went through no change in percent macroalgal cover. “baseline” represents the baseline simulations with no modeled management intervention. In (a–c), each point represents the final % coral cover of a particular reef and box plots showing the inter‐quartile range of the values are placed behind the points to indicate spread; in (a) the points are jittered along the *x*‐axis to make it easier to distinguish individual points.

Overall, all management interventions resulted in an increase in coral cover and a decrease in macroalgal cover across the network relative to the baseline simulation, with the largest gains made in the medium grazing scenario (Figure 4). The high mixed management intervention (M3‐2 km + 25%) led to the largest improvement from the baseline simulation across all grazing scenarios (Figure 4). When the magnitude of the intervention was increased, the coral cover increase was greater (i.e., larger increase in fishery closure (M1‐2 km to M1‐5 km), larger increase in water quality (M2‐10% to M2‐25%), low mixed to high mixed (M3‐2 km + 10% to M3‐2 km + 25%); Figure 4). Water quality interventions (M2‐10%, M2‐25%) led to greater improvements in final coral cover than fishery closure‐based interventions (M1‐2 km, M1‐5 km) (Figure 4a,b), but the fishery closure‐based interventions led to greater declines in macroalgal cover (Figure 4c). When fishery closure‐based interventions (M1‐2 km, M1‐5 km, M3‐2 km + 10%, M3‐2 km + 25%) were applied, coral cover increased in all the reefs, not just those newly included in fishery closures (also, macroalgal cover decreased in some reefs newly and originally in fishery closures; Appendix S6).

In all the grazing scenarios, the high mixed management intervention (M3‐2 km + 25%) led to the most reefs with >30% final coral cover and the most reefs with >30% final coral cover on average (when the average was taken across the three scenarios) (Table 2). Note that overall there was not a large increase in the number of “healthy” (>30% coral cover) reefs (compared to baseline, see Figure 2) under added management interventions under any grazing scenario, with the largest increase observed under the high‐grazing scenario under the M2‐25% and M3‐2 km + 25% management interventions (12–16) (Table 2).

Under the 30%–90% water quality improvement interventions, there was a fairly linear increase in the final percent coral cover with percent improvement (Appendix S7). Water quality improvement interventions of 40%–90% increased final coral cover more than the high mixed management intervention (M3‐2 km + 25%) did (Appendix S7: Figure S1), but even under such large water quality improvement interventions, there were always at least 30 reefs that had a final percent coral cover of less than 1% (Appendix S7: Table S1). When the water quality was improved on only certain scattered reefs (i.e., only the reefs newly included in fishery closures under the M1‐2 km management intervention) (Appendix S9; management interventions M2‐10% scat, M2‐25% scat; “scat” is short for “scattered” in this instance), the increase in coral cover was similar to when those same reefs were newly included in fishery closures (M1‐2 km) and lower than when the water quality of all of the reefs was improved (M2‐10%, M2‐25%) (Appendix S9: Figure S1). The mixed management interventions with scattered water quality improvement (Appendix S9—management interventions “M3‐2 km + 10% scat” and “M3‐2 km + 25% scat”) were also less effective than M3‐2 km + 10% and M3‐2 km + 25% under which all of the reefs improved in water quality (Appendix S9: Figure S1).

### EVPI assessment of grazing scenario information

If we had perfect information about which grazing scenario was the most accurate when choosing the ideal management intervention, there would be no gain in the number of reefs with >30% coral cover as the EVPI value was 0 (Table 2).

### Parameter and initial condition sensitivity analyses

Overall, varying the coral PLD, median coral mortality rate, and rate of overgrowth of mature macroalgae over mature coral did not alter the relative effectiveness of the different management interventions, but it did change the final benthic cover of the reefs (Appendix S4). For the relevant connectivity matrices (i.e., the connectivity matrices that resulted in nonzero final coral cover values), the values of the median coral mortality rate and the values of the rate of overgrowth of mature macroalgae over mature coral that we assessed, the trends that we observed in final benthic cover under each management intervention remained the same (e.g., M1 interventions more effective at decreasing final macroalgal cover but less effective at increasing final coral cover than the M2 interventions; increasing the magnitude of the intervention led to a larger increase in final coral cover) (Appendix S4: Figures S1–13a–c). We also found that the M1 and M3 interventions could still increase the final coral cover of reefs and decrease the final macroalgal cover outside and inside of fishery closures (Appendix S4: Figures S1–S13d–i). The results of the expanded EVPI analysis, which included all of the grazing scenarios in conjunction with the relevant connectivity matrices, the median coral mortality rates, and the rates of overgrowth of macroalgae over mature coral, were also unchanged (EVPI value of 0; Appendix S4: Tables S2, S4, and S6).

Varying the initial conditions did not noticeably alter the relative effectiveness of the different management interventions or change the number of reefs with final coral cover >30% under the low and high‐grazing scenarios (Appendix S8: Table S2, Figure S1–S6a). Under the medium grazing scenario, it did not noticeably alter the effectiveness of the different management interventions beyond increasing the effectiveness of the M3‐2 km + 25% intervention (i.e., larger changes in final benthic cover; one additional reef had a final coral cover >30% compared to the empirical initial condition values) (Appendix S8: Table S2, Figures S1–S6).

## DISCUSSION

The goals of this study were to model the coral cover dynamics of a 75‐reef network in Fiji to assess (1) the interplay between coral and macroalgal dispersal and local reef multiple stability and (2) how the reef network responds to different types of network‐wide management interventions proposed by local conservation practitioners that span the land‐sea interface. The results indicate that a combined land‐sea‐based intervention approach of improving water quality and expanding fishery closures to 2 km within fishing ground boundaries (M3‐2 km + 25%, see Table 1) leads to a larger increase in coral cover in these 75 reefs than doing either in isolation (M1‐2 km and M2‐25%; a sub‐additive increase, Figure 4c) or expanding fishery closures to have a 5‐km radius (M1‐5 km) (an unrealistic increase) (Figure 4, Table 2; but also see Appendices S4 and S8). Furthermore, this result is robust to the grazing scenarios we considered according to our EVPI analysis and robust to varying the coral larval dispersal rate, the median coral mortality rate, the rate of overgrowth of mature macroalgae over mature coral and varying the initial conditions (Figure 4, Table 2; Appendix S4: Tables S2, S4, and S6; Appendix S8: Table S2). Improving the grazing rates at a few reefs (M1‐2 km, M2‐5 km) was less effective than increasing the water quality of all the reefs (M2‐10%, M2‐25%) (Figure 4; Appendix S4: Figures S1–S13b; Appendix S8: Figures S1–S6b), but when both management interventions were performed at the same scale (M2‐10% scat, M2‐25% scat—see Appendix S9) they had similar effects. However, the fishery closure‐based interventions (M1‐2 km, M2‐5 km) had a bigger impact on decreasing the macroalgae cover than even the network‐wide water quality improvement management intervention under the low and high grazing scenarios (Figure 4; Appendix S4: Figures S1–S13c; Appendix S8: Figures S1–S6c).

Increases in grazing rates in certain reefs led to increases in coral cover throughout the reef network due to the dispersal among the reefs in the network. When the grazing rates of a handful of reefs were changed (via Management Intervention M1), the final coral cover of reefs in the system whose grazing rates (and other parameters) were not changed also increased (Appendix S6; also see Appendix S4: Figures S1–S13d–i and Appendix S8: Figures S1–S6d–i). We also found that increasing the median grazing rate of the 75‐reef system (i.e., changing to a more optimistic grazing scenario) led to an overall higher final coral cover for the entire system, even increasing the coral cover of reefs whose grazing rates were so low that they would be expected to collapse to zero coral cover in single‐reef models (Elmhirst et al., 2009; Mumby et al., 2007) (Figure 3a). In particular, the final coral cover of most of the reefs under the high‐grazing scenario was much higher than the final coral cover of most of the reefs under the low‐grazing scenario, under all management interventions (Figure 3a). We posit that, in both cases, this increase in overall coral cover of the studied area is due to the increase in grazing rate of a few specific reefs (or, due to increasing the grazing rate of a certain proportion of reefs in the network beyond some threshold) that are then able to supply enough larvae to reefs with low‐grazing rates to tip them toward a coral‐dominated state. Models such as this one can be used to run “node‐removal” experiments to directly assess which reefs are more or less susceptible to the grazing rates of surrounding reefs and which reefs' grazing rates (if increased) could help the most surrounding reefs. This information could then be used to determine key locations for fisheries management efforts.

The addition of dispersal of coral larvae and macroalgal gametes to the model stabilized what are otherwise local multiple stability dynamics in isolated reefs. This stabilizing role of spatial dynamics reflects long‐standing ecological theory (e.g., Durrett & Levin, 1994). In our model, this was evident in the initial condition sensitivity analysis where we saw only minor changes to the final coral cover of the reefs across the reef network under a wide range of initial benthic cover values under the medium grazing scenario (only one additional reef with >30% final coral cover under the M3‐2 km + 25% management intervention under very high initial coral cover; Appendix S8: Figures S4a, S5a, S6a and Table S2). When reefs have grazing rates of 0.3 (the median grazing rate under the medium grazing scenario in this model) in one‐reef models, both the coral‐ and macroalgal‐dominated states are stable (Elmhirst et al., 2009; Mumby et al., 2007), and the final coral cover of the system exhibits sensitivity to initial conditions. In Greiner, Darling, et al. (2022)'s two‐reef model they found that dispersal between the two reefs resulted in three stable states under medium grazing—a coral‐dominated stable state, an additional mixed coral–macroalgal stable state and a macroalgal‐dominated stable state. The stabilizing effect of dispersal in this larger network could be seen as an extension of that result, as this larger reef network could be resulting in an overall flattening out of the system dynamics due to a manifold increase in stable states (minimizing the unstable regions between them). We also noticed that some reefs with high‐grazing rates had low final coral cover (Figure 3a), corroborating previous findings of high‐grazing reefs having macroalgal‐dominated stable states when connected to low‐grazing reefs by coral larval and macroalgal gamete dispersal in the same two‐reef network model (Greiner, Darling, et al., 2022). Similarly, we found that the relationships between final coral cover and grazing, coral mortality, and net coral migration were less clear than in analogous one‐ and two‐reef models (Elmhirst et al., 2009; Greiner, Darling, et al., 2022; Mumby et al., 2007) (Figure 3). The corresponding relationships between these processes in real‐world reef systems and coral cover are similarly unclear (Graham et al., 2015; Jouffray et al., 2015; Schmitt et al., 2019), indicating that this large reef network model could be a more accurate depiction of real reef dynamics than one‐reef or two‐reef models, though more assessment is needed.

The finding that we are able to make management decisions without reducing our uncertainty in the grazing scenario (EVPI analysis, Table 2; also Appendix S4: Tables S2, S4, S6 and Appendix S8: Table S2) also implies that the multiple stable state dynamics present in the model do not need to be accounted for when making management decisions (Suding & Hobbs, 2009). However, without knowing the full stability properties of the 75‐Reef Fiji Model, we are unable to definitively conclude that dispersal among reefs has stabilized the local multiple stable state dynamics, as it is unclear whether the initial condition and grazing parameter values we explored fully capture the range of dynamics present in this model. That said, since these values were chosen based on our understanding of what best indicates multiple stable state dynamics in the one‐ and two‐reef models (Elmhirst et al., 2009; Greiner, Darling, et al., 2022; Mumby et al., 2007), we can be reasonably confident that—while such dynamics are present locally—they do not appear to influence management effectiveness or alter dynamics at the network scale in this 75‐reef system.

It is also worth noting that the extent to which the integrated land–sea management intervention (M3) improved the coral cover (i.e., changed the final % coral cover) more than the sea‐based interventions (M1) and land‐based interventions (M2) alone varied by grazing scenario (low–almost additive; medium, high– sub‐additive, but note high baseline final coral cover) and was never fully additive or super‐additive (Figure 4b). This relates to findings from Gilby et al. (2016) that found that integrated land–sea management improves outcomes for coral reef systems (this time in Moreton Bay, Australia) but that super‐additive effects are only evident when maximum investment into both types of management occurs (beyond the scope of fisheries management explored here; we limited our management interventions to ones considered to be realistic for Fiji). Also the extent to which the integrated land–sea management intervention (M3) improved the number of reefs with “healthy” coral cover (Birrell et al., 2020; WCS, 2022) more than the sea‐based methods (M1) and land‐based methods (M2) was sub‐additive across all grazing scenarios (other than the low‐grazing scenario, for M3‐2 km + 10%, for which it was additive; Table 2). These results imply that while the integrated land–sea management intervention (M3) improves the final coral cover across the network the most across all grazing scenarios, it might depend on cost–benefit considerations given diminishing returns. However, we caution against focusing too much on this result as (1) a null model would be needed to properly quantify the sign of this interaction, (2) because every management intervention was found to increase coral cover and/or decrease macroalgal cover in the context of the model (see Côté et al., 2016) and (3) we took a fairly simplistic approach to modeling sedimentation and thus our results from this model do not necessarily reflect real‐world land–sea management interventions (discussed further below).

Our model was parameterized from WCS survey data collected between 2017 and 2020 (for the 75 reefs included in the model). While that limited the model to a proportion of a broader reef network, the resulting 75 reef system was large enough to be realistic and enabled far more analysis than if there were no survey data at all. These 75 reefs were also fairly spread out and covered a wide variety of benthic cover and herbivorous fish abundance levels, sedimentation levels, and management regimes (Figure 1). Both fish and invertebrates consume macroalgae and turf algae on reefs; however, we did not have any data on invertebrate herbivore abundances on these reefs. In some reef systems, invertebrate herbivores are the main macroalgae grazers (Francis et al., 2019); however, this topic has been under‐researched in Fiji as studies of macroalgae grazing in Fiji only mention fish herbivores (Bonaldo et al., 2017; Ford et al., 2018; Rasher et al., 2013). There is evidence that there is a greater biomass/abundance of fish herbivores that consume macroalgae than that of fish herbivores that consume other benthic substrate in reefs in Fiji (but no direct comparison of total grazing on different benthic substrata was made; Bonaldo et al., 2017 and Appendix S10: Table S1) and that there is more herbivorous grazing of all substrata (Bonaldo et al., 2017) in protected reefs than unprotected reefs in Fiji (increasing in fairly equal proportions– Bonaldo et al., 2017; Rasher et al., 2012, 2013). In the absence of additional Fiji‐specific information on grazing patterns, and since we only had access to broad fish herbivore classifications (for the reefs included in this study during this time period; see Appendix S3), we followed what was done in previous similar models (Elmhirst et al., 2009; Greiner, Darling, et al., 2022; Mumby et al., 2007) and assumed indiscriminate grazing on algae when formulating the model. It is unlikely that accounting for these potential differences in grazing between macroalgae and other benthic substrate would have changed our conclusions unless the overall rate of grazing on macroalgae compared to turf algae (across all herbivores) was quite different on many reefs–if many reefs had much higher overall grazing rates for turf algae than for macroalgae, more reefs with high macroalgae cover levels may have resulted. Also, our macroalgae cover data did not disambiguate between fleshy macroalgae (“seaweed”) that compete with coral for space and encrusting macroalgae that are not as competitive with corals (Sheppard et al., 2019) (i.e., both types of macroalgae were just recorded as “macroalgae”), meaning that not all of what we were counting as macroalgae behave as we are modeling it to behave (i.e., seaweed‐type macroalgae). Our initial conditions sensitivity analysis (Appendix S8) showed that our management results were largely robust to variations in initial conditions, suggesting that the uncertainty created by this ambiguity likely did not affect our study's findings that the different types of macroalgae observed around Fiji have been compiled (South & Skelton, 2003), but these data are not sufficient to allow us to disambiguate fleshy from encrusting macroalgae in the WCS survey data post hoc. Ideally, future surveys of Fijian reefs should distinguish between these two types of macroalgae to better enable models of this sort to make accurate predictions.

The 75 reefs studied here represent only a few reefs in a larger reef dispersal network for which the survey data necessary to parameterize this model were not available. Thus, we could not assess how coral larval and macroalgal gamete dispersal among the 75 studied reefs and the unstudied reefs in the same reef dispersal network would affect the final coral cover of the 75 reefs (this also affected the scope of the coral larval dispersal rate sensitivity analysis, see Appendix S4). In addition, if we had empirical estimates of coral larval dispersal it would have been possible to ground‐truth the simulated dispersal probabilities (e.g., Bode et al., 2019 for coral reef fish), which suffer from uncertainty in biological and hydrodynamic parameters. We also did not model herbivorous reef fish larval dispersal, even though that likely would have had interesting feedback effects on the grazing rates of the reefs in the model, because we did not have information on reef fish larval dispersal around Fiji. Incorporating reef fish dynamics into the model would make it significantly more complicated in ways that would have made the final results harder to interpret, as we have not seen studies that looked at the impact of reef fish larval dispersal on coral reef cover networks using differential equation‐based models like this one. However, having data for these 75 reefs and accompanying coral larval and macroalgal gamete dispersal connectivity matrices still allowed us to explore the interplay between larval dispersal and three types of management interventions and make predictions about how those interventions might improve future coral cover. Also, the results of our sensitivity analysis imply that our conclusions related to management effectiveness were robust to some variation in coral larval dispersal rates and network structure (Appendix S4; also see Fabina et al. (2015)'s finding that coral recruitment rates were not important for determining response to bleaching). Although the network structure of these other connectivity matrices was overall fairly similar to that of the weighted connectivity matrix (see Appendix S4: Table S7) used to generate the main results, it is interesting that the main management results stayed the same even though the number of reefs disconnected from the main network increased (from 0, see Appendix S4: Table S7; which likely drove the changes to final benthic cover; Appendix S4: Table S6, Figures S9–S13). We also could not find any information on macroalgal gamete dispersal rates among Fijian reefs and thus we approximated macroalgal gamete dispersal assuming a 5‐day passive dispersal process (and otherwise followed the procedure we used to model coral larval dispersal; see Appendices S2 and S3).

There is evidence that sedimentation reduces coral recruitment by making it harder for larvae to disperse (Bainbridge et al., 2018) and settle on turf and other substrates (Speare et al., 2019; Tebbett et al., 2018; Tebbett & Bellwood, 2019) and that it reduces herbivorous grazing levels (Gordon et al., 2016), but we did not incorporate these additional effects of sedimentation into how we modeled our water quality management interventions because we (1) did not want to overcomplicate the model with secondary effects of sedimentation (Nugues & Roberts, 2003; Rogers, 1990), (2) did not have survey data that disambiguated between turf that does impede coral recruitment and those that do not (Wakwella et al., 2020), and (3) because the coral dispersal probabilities in the model were already quite small. It is difficult to anticipate how properly accounting for all of these secondary effects of sedimentation would have altered our conclusions, since the processes that they affect interact with each other in each reef and affect how the reefs interact with each other (but see Fung et al. (2011) for an exploration of how this stressor affects a simpler reef model). However, we did run a sensitivity analysis of different median coral mortality rates (Appendix S4: Table S2 and Figures S1–S4) and found that our management results were robust to the various median values that we explored.

Our analysis of expanding fishery closure size did not include reefs without survey data, some of which are presently in these fishery closures or would be in fishery closures if they were to increase in radius (as we simulate in this study). Having data from more reefs would likely not change our findings regarding the effectiveness and robustness of the fishery closure management interventions but limits our ability to provide specific recommendations for specific Fijian reefs (e.g., recommending adding specific reefs to specific fishery closures). Instead, we focused on assessing how management interventions change the final coral cover of the entire 75‐reef network in this model. Ford et al. (2018) promote measuring resilience‐based metrics (e.g., turf height, coral recruitment rate, and herbivorous fish grazing rates) instead of conventional metrics (e.g., proportional cover of various benthic substrata groups and herbivorous fish biomass) to assess the effectiveness of community‐based management in Fiji. Future research should focus on measuring these resilience‐based metrics and measuring how stressors affect these resilience‐based metrics so that said metrics can be more accurately modeled (in models like the one in this study) to assess how they can be most optimally managed. Future modeling studies should also add realistic levels of demographic and environmental stochasticity, predictions of future disturbances, and future survey data to improve the ability of such models to assess management intervention effectiveness and coral reef cover change over time.

The extent to which our findings regarding management interventions can be generalized to other reef systems depends on how closely this set of 75 reefs in Fiji mimic the dispersal networks and pressures of sedimentation and overfishing/grazing rates of other reef systems. For example, if the overall network structure is different (smaller networks, higher dispersal probabilities among reefs, etc.) the dynamics might change in ways that are hard to anticipate, as the impact of network structure on coral reef multiple stability dynamics is not well understood (two‐reef systems—Baskett et al., 2010; Baskett et al., 2014; Greiner, Darling, et al., 2022; larger rocky reef system—Karatayev & Baskett, 2020 are some of our only reference points; though note that our coral larval dispersal rate sensitivity analysis found that the management effectiveness results were robust to variations in the coral larval dispersal rate, Appendix S4). However, given that coral dispersal is thought to involve high mortality and long distances (Graham et al., 2008), the studied reef network is likely to share similar characteristics with other reef networks, especially when explored at similar spatial scales. Since we looked across a variety of grazing scenarios that encompass a variety of different stability regimes in one‐ and two‐reef systems (Elmhirst et al., 2009; Greiner, Darling, et al., 2022; Mumby et al., 2007), the relationships that we observed among management, dispersal, and grazing rates may be generalizable to other reef networks. However, it is hard to say how generalizable these relationships are because different reef systems vary in herbivore community composition and abundance and because grazing rates are rarely estimated for reef systems, since grazing data are arduous to collect (see Bonaldo et al., 2017; Ford et al., 2018; Rasher et al., 2013; Schmitt et al., 2019 for some examples). Also, we explored a variety of rates of overgrowth of macroalgae over mature coral, median coral mortality rates across the reef system and initial conditions through our sensitivity analyses (Appendices S4 and S8) and found our management results to be robust, providing some evidence that our results may be generalizable. However, it is important to note that we applied these management interventions at many or all reefs across the modeled reef network and then assessed their effects at the network scale; the same types of interventions implemented at just one reef in a network would not have the same effects. Even with all of those caveats, our general conclusions likely hold for other reef systems: (1) dispersal amplifies the effects of management interventions (i.e., increasing the grazing rate at a few reefs in the network can increase coral cover across the network) and can stabilize local multiple stable dynamics, and (2) reef management interventions that tackle water quality and fishing concurrently (i.e., integrated land‐sea management) across a reef network can be more effective for increasing coral cover across the network than singular network‐wide management interventions (corroborated in Gilby et al., 2016).

Models such as this one can aid local management practices by identifying which processes or features of reef systems could be most relevant for shaping the distribution of coral cover in a reef network and identifying the combination of management interventions that could have the most effective ecological outcomes. Integrated land‐sea management is rarely pursued but often thought to be necessary to counteract interacting stressors on coastal ecosystems such as coral reefs (Alvarez‐Romero et al., 2011); within this model framework, we were able to show how effective it could be. This model points to the importance of dispersal, local grazing rates and local mortality rates as key processes structuring coral cover in a reef network. Coral recruitment rate and herbivorous fish grazing rates were also shown to be important to understanding the effectiveness of community‐based management interventions in Fiji by Ford et al. (2018). In particular, this work emphasizes the importance of coral reef connectivity, showing that even the low coral larval dispersal probabilities among reefs in this model change the relationship between local grazing rate and local coral cover. This encourages planning management interventions for connected networks of reefs, rather than for single reefs. This study also encourages the collection of more macroalgal gamete and coral larval dispersal data and grazing rate data and further exploration of the relevance of multiple stable state dynamics across realistic networks of locally multi‐stable ecosystems, so that similar models can be more accurately parameterized and thus be better able to inform specific management practices. In conclusion, this study increases our understanding of how networks of reefs connected by coral larval and macroalgal gamete dispersal behave and motivates the consideration of reef network structure when designing integrated land‐sea management plans for coral reefs in Fiji and around the world.

## AUTHOR CONTRIBUTIONS

Ariel Greiner designed the project, coded the simulations and analyses, and led the writing of the manuscript. Marco Andrello ran the Lagrangian larval dispersal simulations and contributed to and advised on the writing. Marie‐Josée Fortin and Martin Krkošek contributed to project design and advised on analysis and writing. Yashika Nand, Sangeeta Mangubhai, Stacy D. Jupiter, and Amelia Wenger advised on project design, data interpretation, and analysis and contributed to writing. Emily S. Darling advised on project design, orchestrated the collaboration as a whole and aided in data interpretation and analyses.

## CONFLICT OF INTEREST STATEMENT

The authors declare no conflicts of interest.

## Supporting information


Appendix S1.

Appendix S2.

Appendix S3.

Appendix S4.

Appendix S5.

Appendix S6.

Appendix S7.

Appendix S8.

Appendix S9.

Appendix S10.

## Data Availability

The code used to generate the results and analyses in this study (Greiner & Andrello, 2025) is available in Zenodo at https://doi.org/10.5281/zenodo.17340984. The hydrodynamic data used to run the Lagrangian larval dispersal simulations are freely available from the E.U. Copernicus Marine Service Information at https://doi.org/10.48670/moi-00021. The fish and benthic data supporting this research are sensitive and not publicly available. The majority of Fiji coral reef monitoring (fish and benthic data) used in this study are owned by the Wildlife Conservation Society (WCS); these data are available to qualified researchers by contacting the WCS Fiji Country Director at infofiji@wcs.org and requesting a data sharing agreement for the following datasets: 2018_Ovalau Island rapid assessment surveys; 2019_OvalauIsland_BAF_Macmon_surveys; 2019_Macmon_Bua_Ra_Kubulau; 2019_Dama Bureta Waibula and Dawasamu‐WISH ecological survey; 2017_Koro Island rapid assessment surveys; 2020_NamenaAndVatuira coral reef surveys; 2018_Vatu‐i‐Ra reef surveys. The 2017_Northern Lau dataset is owned by the Vatuvara Foundation; these data are available to qualified researchers by contacting katy@vatuvara.org.
