# Supplementary material for: Dispersal can spread management benefits: Insights from a modeled Fijian coral reef network
Source: Ecol Appl. 2025 Dec 8;35(8):e70156. doi: 10.1002/eap.70156 (PMC12683702; doi:10.1002/eap.70156)
Supplement: Supplementary file 1 — Appendix S1. [file EAP-35-e70156-s008.pdf]

Title: Dispersal can spread management benefits: Insights from a modeled Fijian coral reef network

Journal Name: Ecological Applications

Authors: Ariel Greiner, Marco Andrello, Martin Krkošek, Marie-Josée Fortin, Yashika Nand, Stacy D. Jupiter, Sangeeta Mangubhai, Amelia Wenger, Emily S. Darling

Appendix S1: 75 Reef Subset

The Allen coral atlas shows coral reefs all around Fiji (screenshot of Allen coral atlas below). We had access to the coordinates of 551 reef sites from data uploaded to MERMAID from various sources (WCS, WWF, Amanda Ford, Helen Sykes). From that set of 551 reef sites, we only had permission to use the WCS data. Then from the WCS data we could only use data from sites that had both fish and benthic data from the same year from sometime in the 2017-2020 range. We also removed one reef that had incorrect coordinates in our database, this left us with 75 reef sites. We kept all 75 of those reef sites as they were all in the same (larger) network. All 551 reef coordinates were in this same reef network as well.

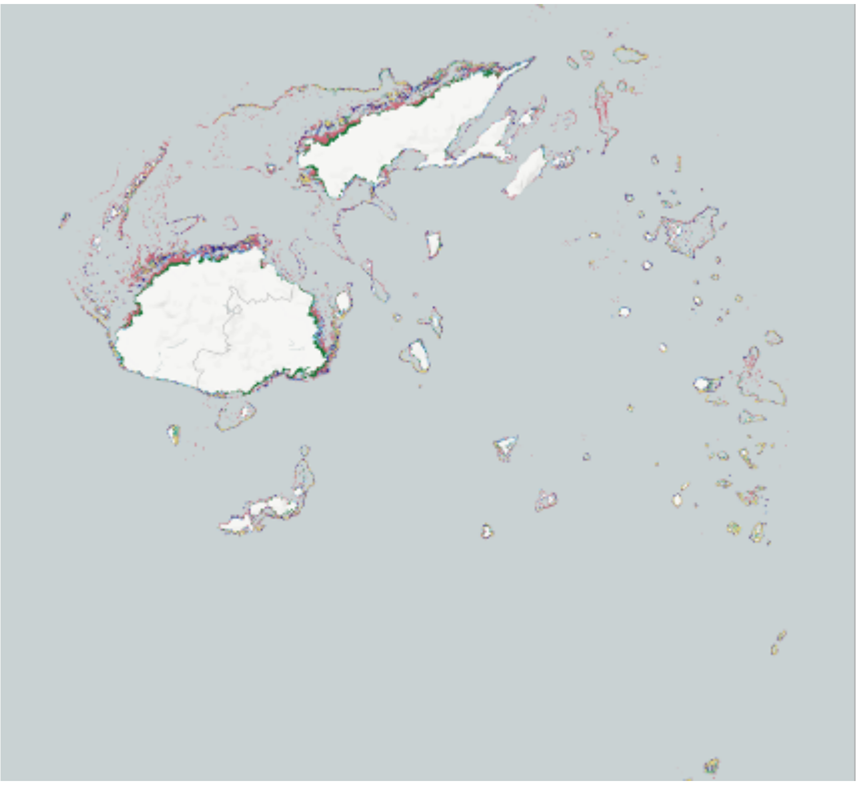

Figure S1: Allen Coral Reef Atlas Fiji Screenshot (2021-09) – Image credit: Allen Coral Atlas

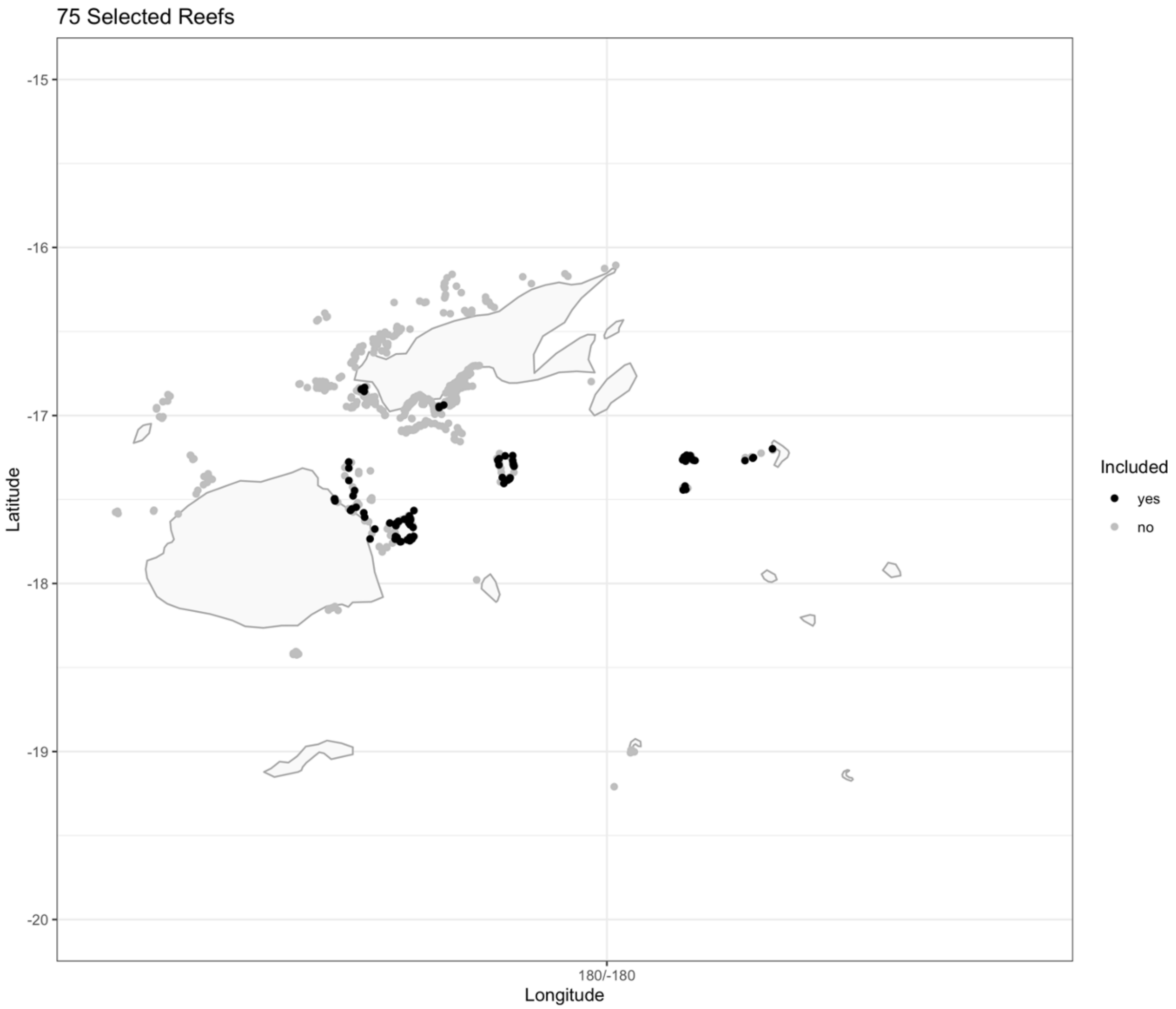

Figure S2: 551 Reef Sites with 75 Reef Subset - Reefs included in the 75-Reef Fiji model labelled in black, all other reefs (551-75) labelled in grey.
