# Supplementary material for: Dispersal can spread management benefits: Insights from a modeled Fijian coral reef network
Source: Ecol Appl. 2025 Dec 8;35(8):e70156. doi: 10.1002/eap.70156 (PMC12683702; doi:10.1002/eap.70156)
Supplement: Supplementary file 2 — Appendix S2. [file EAP-35-e70156-s007.pdf]

Title: Dispersal can spread management benefits: Insights from a modeled Fijian coral reef network

Journal Name: Ecological Applications

Authors: Ariel Greiner, Marco Andrello, Martin Krkošek, Marie-Josée Fortin, Yashika Nand, Stacy D. Jupiter, Sangeeta Mangubhai, Amelia Wenger, Emily S. Darling

Appendix S2: Generating the Larval Dispersal Data to Parameterize the Connectivity Matrices

Lagrangian larval dispersal simulations

We simulated coral larval dispersal from 551 points (hereafter, sites) with known coral reef cover around Fiji using the Lagrangian dispersal simulator Ichthyop v. 3.3.11 (Lett et al., 2008). Daily sea current velocities were taken from GLORYS12V1, a global ocean eddy-resolving reanalysis at 1/12° horizontal resolution with 50 vertical levels (https://doi.org/10.48670/moi-00021). Although the horizontal resolution of GLORYS12V1 is likely too coarse for studying particle dispersal nearshore, it was the finest resolution available for this study region. Also note that Ichthyop interpolates the velocity vectors between the 1/12° x 1/12° grid points using a Runge-Kutta 4th order scheme and thus velocities between the grid points may be approximated.

In Ichthyop, virtual larvae were released at the coordinates of each of the 551 sites. Due to the coarse resolution of the GLORYS12V1 grid, 55 of the 551 sites fell onto land masses. For these sites, virtual larvae were released from the nearest grid point located in water, which was found

using a nearest-neighbour search implemented in the 'nn2' function in the R package 'RANN' v. 2.6.1 (Arya et al., 2019). Therefore, the final list of release sites was made of 496 original site coordinates and 55 grid points. We released 100 virtual larvae in each of the 551 release sites. See the R script Create_initial_positions.R in https://doi.org/10.5281/zenodo.17340984 for more details on release locations.

Generic coral larvae were released on the first day of each month, at noon, in each year between 2009 and 2018 (100 larvae * 12 months * 10 years = 12,000 larvae per site). The time step of computation was 3600 seconds, which was sufficiently small to satisfy the Courant–Friedrichs–Lewy (CFL) condition (i.e. the space travelled by a larva during one computation step must be shorter than the size of the grid cells; van Sebille et al., 2018). The advection process followed a Runge-Kutta 4th order scheme. Larvae hitting the coastline were sent back to water ("bouncing" coastline behaviour in Ichthyop). Horizontal diffusion was applied via a random walk for individual larvae to account for sub-grid-scale hydrodynamics associated with coastal features (reefs, bays, gulfs, etc.) following Peliz et al. (2007), with a horizontal diffusion coefficient $K = \varepsilon^{1/3} l^{4/3}$, where $\varepsilon = 10^{-9}$ $m^2 s^{-3}$ is the constant turbulent dissipation rate and l is the distance between grid points. Larvae were kept drifting for 130 days and the positions of larvae were recorded every 120 time steps, i.e., every 5 days. This allowed us to simulate pelagic larval durations (PLDs) at 5 day increments over the plausible range of PLD reported in the literature [5-130 days; stemming from Treml et al., 2008 (15-60 days PLD); Wood et al., 2014 (1-120 days PLD); Schill et al., 2015 (30 days PLD); Treml et al., 2015 (10-60 days PLD); Hock et al., 2017 (7-14 days PLD); Romero-Torres et al., 2018 (30-150 days PLD); Gamoyo et al., 2019 (15-60 days PLD)] as we lacked specific data on reasonable PLDs for Fijian coral. No active swimming was implemented. See the Ichthyop configuration files in https://doi.org/10.5281/zenodo.17340984 (e.g., Fiji_2009_12months.xml; one file per simulation year) for full parametrization details.

We created a circular buffer with a radius = 0.001 degree (approximately 110 metres) around each release site. At each PLD, larvae whose coordinates were located inside the buffer were considered recruited in that site for the calculation of the connectivity matrices. The positions of larvae relative to the circular buffers was assessed using the function 'gContains' in the R package 'rgeos' v. 0.5-8 (Bivand & Rundel, 2021). For each simulated PLD (PLDs of {5, 10, 15, ..., 120, 125, 130} days), we calculated a connectivity matrix (CM_{PLD}) containing the pairwise probabilities of larval dispersal between the 551 sites. To calculate the CM_{PLD}, for each reef we divided the number of larvae that landed in said reef each month by 100 and then added all those /month values together. See the R script Create.dispersal.matrix.R in https://doi.org/10.5281/zenodo.17340984 for more details on calculation of dispersal matrices.

Weighted connectivity matrices

We then took a weighted average across all the PLD connectivity matrices to generate a final connectivity matrix (CM_{Final}) reflecting the effect of larval mortality on dispersal probabilities

following Wood et al. (2014). In Wood et al. (2014)'s model, all larvae are competent to settle by day 10 (10% of the cohort become capable of settling per day, from day 1 to day 10). To reflect that without needing to explicitly include a competency model, we did not include the PLD = 5 connectivity matrix in the weighted average (i.e., gave it a weighted average of 0). Wood et al. (2014) calculated the proportion of larvae surviving at time t+1 (where t indicates day), S_{t+1}, as:

$$S_{t+1} = (S_t)e^{-\lambda t}\ , \lambda = ln(2)/(35)$$

where λ is the mortality rate/decay constant and 35 days is the half-life for decay. To mimic that, we set S_{10} = 1 and then $S_{15} = (S_{10})e^{-10\lambda}$ and then define the proportion of larvae that settle at PLD=10 as $S_{15} - S_{10}$. We then multiplied CM_{10} by $S_{15} - S_{10}$. We then repeated this for all PLDs until PLD=130 and then calculated CM_{Final} as a weighted average using the equation below.

$$CM_{Final} = \frac{\sum_{t=\{10,15,\ldots,125,130\}}(S_{t+1} - S_t) * CM_t}{\sum_{t=\{10,15,\ldots,125,130\}}(S_{t+1} - S_t)}$$

References
Arya S., D. Mount, S. E. Kemp and G. Jefferis. 2019. "RANN: Fast Nearest Neighbour Search (Wraps ANN Library) Using L2 Metric." https://CRAN.R-project.org/package=RANN
Bivand R. and C. Rundel. 2021. "rgeos: Interface to Geometry Engine - Open Source ('GEOS')." https://CRAN.R-project.org/package=rgeos
Gamoyo, M., D. Obura, and C. J. C. Reason. 2019. "Estimating connectivity through larval dispersal in the Western Indian Ocean." *Journal of Geophysical Research: Biogeosciences* **124**:2446-2459.
Greiner, A., Andrello, M. 2025. ArielGreiner/Fiji_StabilityConnectivitySimulation: Associated with Ecological Applications Publication (v.1). Zenodo. https://doi.org/10.5281/zenodo.17340984
Hock, K., N. H. Wolff, J. C. Ortiz, S. A. Condie, K. R. Anthony, P. G. Blackwell and P. J. Mumby. 2017. Connectivity and systemic resilience of the Great Barrier Reef. *PLoS biology* **15**:e2003355.
Lett, C., P. Verley, C. Mullon, C. Parada, T. Brochier, P. Penven, B. Blanke. 2008. "A Lagrangian tool for modelling ichthyoplankton dynamics." *Environmental Modelling & Software* **23**:1210–1214.
Peliz, A., P. Marchesiello, J. Dubert, M. Marta-Almeida, C. Roy, and H. Queiroga. 2007. "A study of crab larvae dispersal on the Western Iberian Shelf: Physical processes." *Journal of Marine Systems* **68**:215-236.
Romero-Torres, M., E. A. Treml, A. Acosta, and D. A. Paz-García. 2018. "The Eastern Tropical Pacific coral population connectivity and the role of the Eastern Pacific Barrier." *Scientific Reports* **8**:9354.
Schill, S. R., G. T. Raber, J. J. Roberts, E. A. Treml, J. Brenner, and P. N. Halpin. 2015. "No reef is an island: integrating coral reef connectivity data into the design of regional-scale marine protected area networks." *PLoS One* **10**:e0144199.

Treml, E. A., P. N. Halpin, D. L. Urban, and L. F. Pratson. 2008. "Modeling population connectivity by ocean currents, a graph- theoretic approach for marine conservation." *Landscape Ecology* **23**:19–36

Treml, E. A., J. Roberts, P. N. Halpin, H. P. Possingham, and C. Riginos. 2015. "The emergent geography of biophysical dispersal barriers across the Indo-West Pacific." *Diversity and Distributions* **21**:465-476.

Van Sebille, E., S. M. Griffies, R. Abernathey, T. P. Adams, P. Berloff, A. Biastoch, B. Blanke et al. 2018. "Lagrangian ocean analysis: Fundamentals and practices." *Ocean modelling* **121**:49-75.

Wood, S., C. B. Paris, A. Ridgwell, and E. J. Hendy. 2014. "Modelling dispersal and connectivity of broadcast spawning corals at the global scale." *Global Ecology and Biogeography* **23**:1-11.
