# Supplementary material for: Dispersal can spread management benefits: Insights from a modeled Fijian coral reef network
Source: Ecol Appl. 2025 Dec 8;35(8):e70156. doi: 10.1002/eap.70156 (PMC12683702; doi:10.1002/eap.70156)
Supplement: Supplementary file 3 — Appendix S3. [file EAP-35-e70156-s009.pdf]

Title: Dispersal can spread management benefits: Insights from a modeled Fijian coral reef network

Journal Name: Ecological Applications

Authors: Ariel Greiner, Marco Andrello, Martin Krkošek, Marie-Josée Fortin, Yashika Nand, Stacy D. Jupiter, Sangeeta Mangubhai, Amelia Wenger, Emily S. Darling

Appendix S3: 75-Reef Fiji Model Parameterization

The coral and macroalgae cover data used to set the initial values of C_i and M_i came from WCS Fiji survey data accessed via the MERMAID dashboard (https://dashboard.datamermaid.org/) and the initial values for F_i were calculated from the initial values of C_i and M_i using equation (1.3). We define 'a' as the overgrowth rate of mature coral by mature macroalgae. As no information on this parameter was available for Fiji specifically, we used the value from Mumby et al., (2007), Elmhirst et al., (2009), and Greiner et al., (2022) (a = 0.1). The grazing rate for each reef i (g_i) was obtained from the total herbivorous fish density recorded on each reef ('macroalgal herbivorous fish' and 'detritivore herbivorous fish' categories in MERMAID). Since we did not have per-capita herbivorous fish consumption rates on these reefs, we used herbivorous fish density as a proxy for g_i. Since past modeling studies (Elmhirst et al., 2009; Greiner et al., 2022; Mumby et al., 2007) only explored grazing rates (g_i) between 0 and 1, we set the highest herbivorous fish density recorded for Fiji in the MERMAID database (1573.33kg/ha for 'macroalgal herbivorous fish' and 5172.61kg/ha for 'detritivore herbivorous fish', retrieved on November 25, 2021) as a density of 1 and scaled the rest accordingly (i.e. by dividing all of the macroalgal herbivorous fish densities by the highest macroalgal herbivorous fish density and dividing all of the detritivore herbivorous fish densities by the highest detritivore

herbivorous fish density). Given that we had herbivore density estimates but not grazing rates, we explored three grazing scenarios for the 75 reefs to roughly account for the missing consumption rate (as $g_i = \sum_{species\ k}(c_k * n_k)$, where $c_k =$ per-capita herbivorous fish consumption rate for species k and n_k = density of species k). We chose one non-species specific consumption rate value for each scenario so that the median grazing rate (herbivore density estimate obtained from the WCS data for each reef*consumption rate value chosen for each scenario) for all 75 reefs was 0.1, 0.3, and 0.5 (respectively), as these three grazing values characterize different stability regimes in one-reef (Elmhirst et al., 2009; Mumby et al., 2007) and two-reef (Greiner et al., 2022) models of coral reef dynamics. In single-reef models grazing rates of 0.1, 0.3, and 0.5 characterize a macroalgal-dominated stable state, bistability (both a macroalgal-dominated stable state and a coral-dominated stable state) and a coral-dominated stable state respectively. These grazing scenarios will be referred to as 'low' (median of 0.1), 'medium' (median of 0.3) and 'high' (median of 0.5).

To estimate the proportion of coral larvae and macroalgal gametes dispersing among the reefs, we calculated coral and macroalgal connectivity matrices which depict the probability that a coral larva (or a macroalgal gamete, respectively) will disperse from any one reef to each other reef. To do so, we first simulated propagule dispersal from 551 reef coordinates around Fiji (from WCS MERMAID data from Fiji, https://dashboard.datamermaid.org/) from 2009-2018 via Lagrangian simulations in Ichthyop v. 3.3.11 (Lett et al., 2008). To calculate macroalgal gamete dispersal, we used a PLD = 5 days, since most macroalgae dispersal is thought to only occur over short distances (Deysher & Norton, 1981; Mumby, 2006; Stiger & Payri, 1999), though little is known about macroalgae dispersal distances in general. To calculate coral larval dispersal, we simulated a range of PLD values (5-130 days; stemming from Gamoyo et al., 2019; Hock et al., 2017; Romero-Torres et al., 2018; Schill et al., 2015; Treml et al., 2008; Treml et al., 2015; Wood et al., 2014) then took a weighted average of larval dispersal probabilities over PLDs following Wood et al. (2014) to reflect the decay in larval abundance due to mortality as PLD increases. We then subset this connectivity matrix to just include the dispersal among the 75 reefs in the model; any larvae that did not disperse to one of those 75 reefs was considered lost (>99% of the larvae were lost in the final connectivity matrix). Note that all 551 reef coordinates were determined to be in the same network as each other. We focus on a net migration of coral larvae parameter (the amount of larvae that enter reef i - the amount of larvae that leave reef i, for each reef i (i:{1-75})) to capture the amount of coral larval dispersal each reef in the network experiences.

As we only had dispersal probability values, we ran simulations to calculate a larval production rate for coral (r) and a gamete production rate for macroalgae (γ) that would ensure coral and/or macroalgal persistence under system-wide grazing rate parameterizations known to lead to coral and/or macroalgal persistence. We calculated the values for r and γ by determining the smallest value of both terms (r = 125 larvae t^{-1}, and γ = 900 gametes t^{-1}) that ensured that when coral

mortality (d) was set to be constant across reefs (d_i = 0.24, following from Elmhirst et al. (2009)) and grazing was set to be high (g_i = 0.5) or medium (g_i = 0.3) in all reefs, at least one reef had >30% final coral cover (in the model) and when grazing was set to be medium (g_i = 0.3) or low (g_i = 0.1) in all reefs, at least one reef had >30% final macroalgal cover (in the model). This method is informed by empirical results (Done, 1992; Graham et al., 2015; Hughes, 1994; Schmitt et al., 2019) and modeling studies (Elmhirst et al., 2009; Greiner et al., 2022; Mumby et al., 2007) of this sort of behaviour on reef systems (high grazing rates leading to high coral cover, low grazing rates leading to high macroalgal cover. The >30% cut-off was chosen as reefs with >30% coral cover are considered to be 'healthy' enough to maintain biodiversity and fisheries over time (Birrell et al., 2020; WCS 2022). Note that even at very high (>10,000) values of r and/or γ and at a constant grazing rate (g_i), it was not possible to ensure that at high grazing (g_i = 0.5, for all i) all reefs would have >30% coral cover and at low grazing (g_i = 0.1, for all i), all reefs would have >30% macroalgae cover.

We derived the coral mortality parameter values for each reef (d_i) using the sedimentation pressures in the waters around Fiji calculated by Andrello et al. (2022). The exact relationship between sedimentation and coral mortality is not known for Fijian reefs, so we drew on information from other areas. Increasing sedimentation levels on reefs has been shown to increase coral mortality by reducing the amount of light that reaches the coral, facilitating transmission of disease vectors, and physically obstructing coral surfaces (Bainbridge et al., 2018; Nugues & Roberts, 2003). The effect of sedimentation on coral in the Indo-Pacific has been modeled as increasing the coral mortality rate (Fung et al. 2011) and has been found to do so in empirical studies (Bainbridge et al., 2018; Erftemeijer et al., 2012; Fabricius, 2005). Andrello et al. (2022) delineated the world's coral reef habitat into square grid cells of 0.05° (~5 km) side and calculated a sedimentation pressure value for each grid cell. Thus, to assign each reef a sedimentation pressure value, we first mapped each of our 75 reef coordinates to one of Andrello et al. (2022)'s grid cells. As two of the reefs did not fall within a grid cell, we assigned those the value of the nearest sedimentation grid cell. The sediment values reported by Andrello et al. (2022) are scaled between 0 and 1. To transform these into coral mortality rates, we first scaled the sedimentation pressure values for each reef so the median value was 0.24. We did not have any data on what the median coral mortality value might be in Fiji (or the coral mortality of any particular reef in Fiji). Hence, we used 0.24 as our median value so that our results would be comparable with the one-reef (Elmhirst et al., 2009) and two-reef models (Greiner et al., 2022) that used 0.24 as the coral mortality value. Since the coral mortality value of any reef is greater than 0 (Bythell et al., 1993), we assigned all reefs in those cells coral mortality values of 0.02 (much lower than the non-zero mortality values used in the model (range = 0.158 - 0.313; Figure 1e). This ensured that the values for coral mortality used in this study approximated the ranges used in Fung et al. (2011) (0.02, 0.3), Fabina et al. (2015) (0.002-0.4) and McManus et al. (2019) (0.1-0.5) and mimics coral mortality values used in Baskett et al. (2014) (0.25).

<u>References</u>

Andrello, M., E. S. Darling, A. Wenger, A. F. Suárez-Castro, S. Gelfand, and G. N. Ahmadia. 2022. "A global map of human pressures on tropical coral reefs." *Conservation Letters* **15**:e12858.

Bainbridge, Z., S. Lewis, R. Bartley, K. Fabricius, C. Collier, J. Waterhouse, A. Garzon-Garcia, et al. 2018. "Fine sediment and particulate organic matter: A review and case study on ridge-to-reef transport, transformations, fates, and impacts on marine ecosystems." *Marine Pollution Bulletin* **135**: 1205-1220.

Baskett, M. L., N. S. Fabina, and K. Gross. 2014. "Response diversity can increase ecological resilience to disturbance in coral reefs." *The American Naturalist* **184**:E16-E31.

Birrell, C. L., E. Sola, R. H. Bennett, D. van Beuningen, H. M. Costa, J. J. Sitoe, N. Sidat, S. Fernando, E.S. Darling, N.A. Muthiga and T. R. McClanahan. 2020. "A summary of WCS knowledge of the state of coral reefs in Mozambique." Wildlife Conservation Society, Maputo, Mozambique. https://biblioteca.biofund.org.mz/wp-content/uploads/2021/03/1616752045-2020_WCS_Coral_Reefs_in_Mozambique.pdf

Bythell, J. C., E. H. Gladfelter, and M. Bythell. 1993. "Chronic and catastrophic natural mortality of three common Caribbean reef corals." *Coral Reefs* **12**:143-152.

Deysher, L., and T. A. Norton. 1981. "Dispersal and colonization in Sargassum muticum (Yendo) Fensholt." *Journal of experimental marine biology and ecology* **56**:179-195.

Done, T. 1992. "Phase shifts in coral reef communities and their ecological significance." *Hydrobiologia* **247**:121–132.

Elmhirst, T., S. R. Connolly, and T. P. Hughes. 2009. Connectivity, regime shifts and the resilience of coral reefs. *Coral Reefs* **28**:949-957.

Erftemeijer, P. L., B. Riegl, B. W. Hoeksema, and P. A. Todd. 2012. "Environmental impacts of dredging and other sediment disturbances on corals: a review." *Marine pollution bulletin* **64**:1737-1765.

Fabina, N. S., M. L. Baskett, and K. Gross. 2015. "The differential effects of increasing frequency and magnitude of extreme events on coral populations." *Ecological Applications* **25**:1534-1545.

Fabricius, K. E. 2005. "Effects of terrestrial runoff on the ecology of corals and coral reefs: review and synthesis." *Marine pollution bulletin* **50**:125-146.Fung et al. 2011

Gamoyo, M., D. Obura, and C. J. C. Reason. 2019. "Estimating connectivity through larval dispersal in the Western Indian Ocean." *Journal of Geophysical Research: Biogeosciences* **124**:2446-2459.

Graham, N. A., S. Jennings, M. A. MacNeil, D. Mouillot, and S. K. Wilson. 2015. "Predicting climate-driven regime shifts versus rebound potential in coral reefs." *Nature* **518**:94-97.

Greiner, A., E. S. Darling, M. -J. Fortin, and M. Krkošek. 2022. "The combined effects of dispersal and herbivores on stable states in coral reefs." *Theoretical Ecology* **15**:321-335.

Hock, K., N. H. Wolff, J. C. Ortiz, S. A. Condie, K. R. Anthony, P. G. Blackwell and P. J. Mumby. 2017. Connectivity and systemic resilience of the Great Barrier Reef. *PLoS biology* **15**:e2003355.

Hughes, T. P. 1994. "Catastrophes, phase shifts, and large-scale degradation of a Caribbean coral reef." *Science* **265**:1547-1551.

McManus, L. C., J. R. Watson, V. V. Vasconcelos, and S. A. Levin. 2019. "Stability and recovery of coral-algae systems: the importance of recruitment seasonality and grazing influence." *Theoretical Ecology* **12**:61-72.

Mumby, P. J. 2006. "The impact of exploiting grazers (Scaridae) on the dynamics of Caribbean coral reefs." *Ecological applications* **16**:747-769.

Mumby, P. J., A. Hastings, and H. J. Edwards. 2007. "Thresholds and the resilience of Caribbean coral reefs." *Nature* **450**:98.

Nugues, M. M., and C. M. Roberts. 2003. "Coral mortality and interaction with algae in relation to sedimentation." *Coral reefs* **22**:507-516.

Romero-Torres, M., E. A. Treml, A. Acosta, and D. A. Paz-García. 2018. "The Eastern Tropical Pacific coral population connectivity and the role of the Eastern Pacific Barrier." *Scientific Reports* **8**:9354.

Schill, S. R., G. T. Raber, J. J. Roberts, E. A. Treml, J. Brenner, and P. N. Halpin. 2015. "No reef is an island: integrating coral reef connectivity data into the design of regional-scale marine protected area networks." *PLoS One* **10**:e0144199.

Schmitt, R. J., S. J. Holbrook, S. L. Davis, A. J. Brooks, and T. C. Adam. 2019. "Experimental support for alternative attractors on coral reefs." *Proceedings of the National Academy of Sciences* **116**:4372-4381.

Stiger, V., and C. E. Payri. 1999. "Spatial and temporal patterns of settlement of the brown macroalgae Turbinaria ornata and Sargassum mangarevense in a coral reef on Tahiti." *Marine Ecology Progress Series* **191**:91-100.

Treml, E. A., P. N. Halpin, D. L. Urban, and L. F. Pratson. 2008. "Modeling population connectivity by ocean currents, a graph- theoretic approach for marine conservation." *Landscape Ecology* **23**:19–36

Treml, E. A., J. Roberts, P. N. Halpin, H. P. Possingham, and C. Riginos. 2015. "The emergent geography of biophysical dispersal barriers across the Indo-West Pacific." *Diversity and Distributions* **21**:465-476.

Wildlife Conservation Society (WCS). 2022. "Launching a Decade of Action for Coral Reefs." https://cdn.wcs.org/2021/04/21/99xudme990_4.16.21_English_CBD_Rec_2_Pager.pdf?_gl=1*1f71558*_ga*MTk4MTYyMzY4Ni4xNjc0ODM0MjI1*_ga_BTX9HXMYSX*MTY4MDE5Nzk2MC4xNi4wLjE2ODAxOTc5NjAuNjAuMC4w

Wood, S., C. B. Paris, A. Ridgwell, and E. J. Hendy. 2014. "Modelling dispersal and connectivity of broadcast spawning corals at the global scale." *Global Ecology and Biogeography* **23**:1-11.
