# Supplementary material for: Dispersal can spread management benefits: Insights from a modeled Fijian coral reef network
Source: Ecol Appl. 2025 Dec 8;35(8):e70156. doi: 10.1002/eap.70156 (PMC12683702; doi:10.1002/eap.70156)
Supplement: Supplementary file 4 — Appendix S4. [file EAP-35-e70156-s010.pdf]

Title: Dispersal can spread management benefits: Insights from a modeled Fijian coral reef network

Journal Name: Ecological Applications

Authors: Ariel Greiner, Marco Andrello, Martin Krkošek, Marie-Josée Fortin, Yashika Nand, Stacy D. Jupiter, Sangeeta Mangubhai, Amelia Wenger, Emily S. Darling

Appendix S4: Results of the Parameter Sensitivity Analyses

Median Coral Mortality Rate Sensitivity Analysis

As discussed in Appendix S3, the coral mortality rates for the 75 reefs were derived from sedimentation levels from Andrello et al. (2022), scaled around a median coral mortality rate (d_{med}) that came from single-reef and two-reef versions (Elmhirst et al., 2009; Greiner et al., 2022) of the 75-Reef Fiji Model (i.e. $d_{med} = 0.24$). To assess the sensitivity of the management results to this choice of median coral mortality rate value, we ran the 75-Reef Fiji Model under various values of d_{med} (0.12, 0.16, 0.36, 0.48) chosen to span a similar range of values used in the literature for the coral mortality parameter in coral reef benthic cover models (Mumby et al., 2007; Blackwood et al., 2012; Fung et al., 2011; Fabina et al., 2015; McManus et al., 2019) while also using reasonable increments (multiplying $d_{med} = 0.24$ by 1/2, 2/3, 3/2, 2 respectively; same as those used for the sensitivity analysis of a below).

Overall, varying the median coral mortality rate did not alter the relative effectiveness of the different management interventions but it did change the final benthic cover of the reefs (Table S2, Figures S1-4). For the values of the median coral mortality rate that we assessed, the trends that we observed in final benthic cover under each management intervention remained the same (e.g. M1 interventions more effective at decreasing final macroalgal cover but less effective at

increasing final coral cover than the M2 interventions; increasing the magnitude of the intervention led to a larger increase in final coral cover) (Figures S1-4a-c,). We also found that the M1 and M3 interventions could still increase the final coral cover of reefs and decrease the final macroalgal cover outside and inside of fishery closures (Figures S1-4d-i). The results of the expanded EVPI analysis, which included all the grazing scenarios in conjunction with the the median coral mortality rates, were also unchanged (EVPI value of 0; Table S2).

Table S1: *Overall Scenarios* - This table describes all the overall scenarios (Grazing* d_{med} Scenarios) shown in Table S2 below.

Overall Scenario	Grazing Scenario	d_{med} value
1	Low	0.12
2	Medium	0.12
3	High	0.12
4	Low	0.16
5	Medium	0.16
6	High	0.16
7	Low	0.24
8	Medium	0.24
9	High	0.24
10	Low	0.36
11	Medium	0.36
12	High	0.36
13	Low	0.48
14	Medium	0.48
15	High	0.48

Table S2: *Expected Value of Perfect Information* - The numbers in the table below represent the number of reefs under each management intervention and grazing scenario whose final percent coral cover is >30%.

Overall Scenario/Management Intervention	1	2	3	4	5	6	7	8	9	10	11	12	13	14	15	Avg across scenarios
1. M1-2km	5	24	24	3	18	18	**1**	11	12	**0**	5	9	**0**	2	4	9.33
2. M1-5km	6	24	24	3	18	18	**1**	12	12	**0**	6	9	**0**	2	4	9.5

3. M2-10%	5	27	27	2	20	20	0	12	14	**0**	4	10	**0**	1	5	10.08
4. M2-25%	10	**32**	**32**	4	**24**	**24**	**1**	13	**16**	**0**	**9**	**12**	**0**	3	**9**	13.25
5. M3-2km+10%	7	27	27	3	20	20	**1**	12	14	**0**	7	10	**0**	2	5	10.67
6. M3-2km+25%	**13**	**32**	**32**	**5**	**24**	**24**	**1**	**15**	**16**	**0**	**9**	**12**	**0**	**5**	**9**	*13.75*
Avg across management interventions	7.14	27.14	27.14	3	20.29	20.29	0.83	12.5	14	0	6.14	10.14	0	2.29	5.43	

Note: The numbers in **bold** represent the highest values in each column (i.e. the management intervention that results in the highest number of reefs with a percent coral cover >30% under that grazing scenario) and the value in *italics* (13.75) is the highest average number of reefs with percent coral cover >30% across all management interventions (averaged across the scenarios). Taking the average of the highest value in each of the three middle columns (i.e. the numbers in **bold** (i.e. (13+32+32+5+24+24+1+15+16+0+9+12+0+5+9)/15 = 13.75)) and then subtracting the highest value in the final column (i.e. the number in *italics*: 13.75) gives you the EVPI value for this analysis (0).

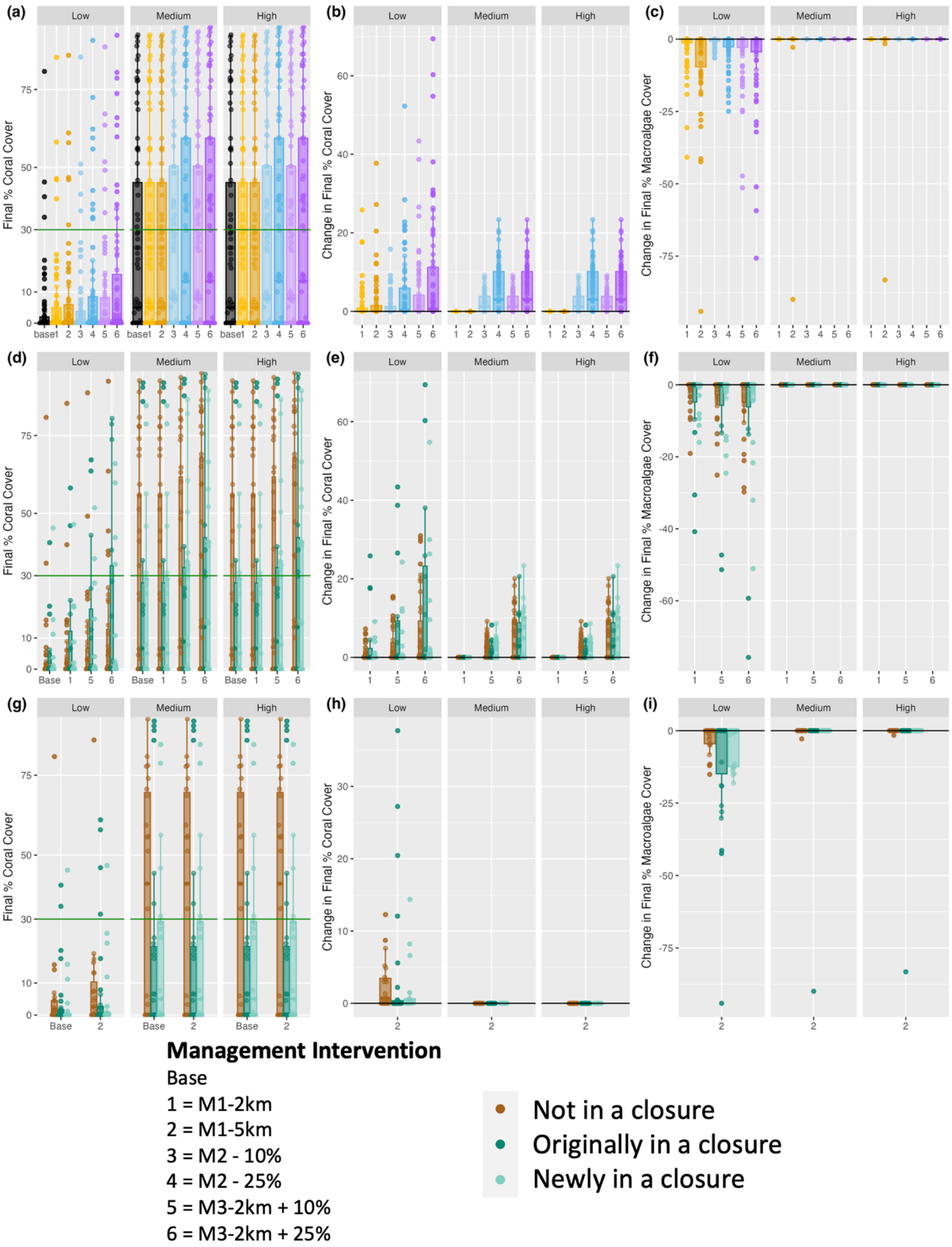

(a)
(b)
(c)
(d)
(e)
(f)
(g)
(h)
(i)
Low
Medium
High
Final % Coral Cover
Change in Final % Coral Cover
Change in Final % Macroalgae Cover
Management Intervention
Base
1 = M1-2km
2 = M1-5km
3 = M2 - 10%
4 = M2 - 25%
5 = M3-2km + 10%
6 = M3-2km + 25%
Not in a closure
Originally in a closure
Newly in a closure

Figure S1. *Effects of the Management Interventions When* d_{med} *= 0.12* - Each panel shows the effect of the management intervention on the final coral cover of each reef, while each panel shows the effect of the management under each grazing scenario. (d, e, f) Effects of fishery closure management interventions bound by fishing ground restrictions; the reefs are separated by management status under the M1, M3 management interventions, with the teal dots representing reefs that are only under fishery closure protection when the fishery closure is extended and the light blue dots representing reefs that were originally and remain under fishery closure protection under all the interventions and the baseline simulation. (g, h, i) Effects of fishery closure management intervention not bound by fishing ground restrictions; the reefs are separated by management status under the 5km fishery closure increase management intervention (i.e., M1-5km), with the teal dots representing reefs that are only under fishery closure protection when the fishery closure is extended to ~5km (i.e., M1- 5km) and the light blue dots representing reefs that were originally and remain under fishery closure protection under all the interventions and the baseline simulation. (a, d, g) Final percent coral cover in each reef, with a green line at 30% indicating a healthy reef (Birrell et al., 2020; WCS 2022). (b, e, h) Difference in the percent coral cover in each reef between each management intervention and the baseline simulation; the black line at 0 indicates the reefs that went through no change in percent coral cover. (c, f, i) Difference in the percent macroalgal cover in each reef between each management intervention and the baseline simulation; the black line at 0 indicates the reefs that went through no change in percent macroalgal cover. 'Base' represents the baseline simulations with no modeled management intervention. Each point represents the final % coral cover of a particular reef and box plots showing the inter-quartile range of the values are placed behind the points to indicate spread; in (a, d, g) the points are jittered along the *x*-axis to make it easier to distinguish individual points.

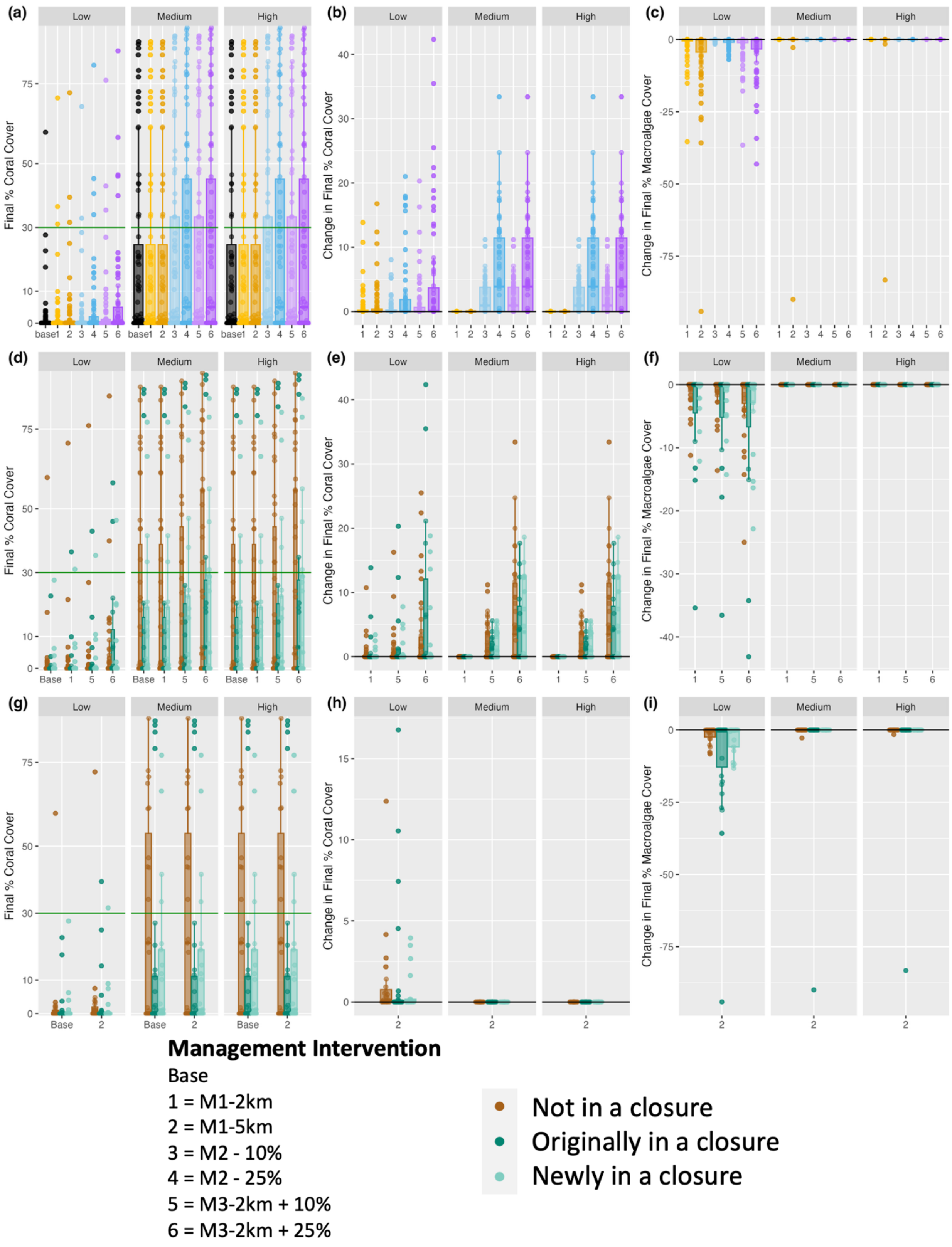

Figure S2. *Effects of the Management Interventions When d_{med} = 0.16* - Each panel shows the effect of the management intervention on the final coral cover of each reef, while each panel

shows the effect of the management under each grazing scenario. (d, e, f) Effects of fishery closure management interventions bound by fishing ground restrictions; the reefs are separated by management status under the M1, M3 management interventions, with the teal dots representing reefs that are only under fishery closure protection when the fishery closure is extended and the light blue dots representing reefs that were originally and remain under fishery closure protection under all the interventions and the baseline simulation. (g, h, i) Effects of fishery closure management intervention not bound by fishing ground restrictions; the reefs are separated by management status under the 5km fishery closure increase management intervention (i.e., M1-5km), with the teal dots representing reefs that are only under fishery closure protection when the fishery closure is extended to ~5km (i.e., M1- 5km) and the light blue dots representing reefs that were originally and remain under fishery closure protection under all the interventions and the baseline simulation. (a, d, g) Final percent coral cover in each reef, with a green line at 30% indicating a healthy reef (Birrell et al., 2020; WCS 2022). (b, e, h) Difference in the percent coral cover in each reef between each management intervention and the baseline simulation; the black line at 0 indicates the reefs that went through no change in percent coral cover. (c, f, i) Difference in the percent macroalgal cover in each reef between each management intervention and the baseline simulation; the black line at 0 indicates the reefs that went through no change in percent macroalgal cover. 'Base' represents the baseline simulations with no modeled management intervention. Each point represents the final % coral cover of a particular reef and box plots showing the inter-quartile range of the values are placed behind the points to indicate spread; in (a, d, g) the points are jittered along the *x*-axis to make it easier to distinguish individual points.

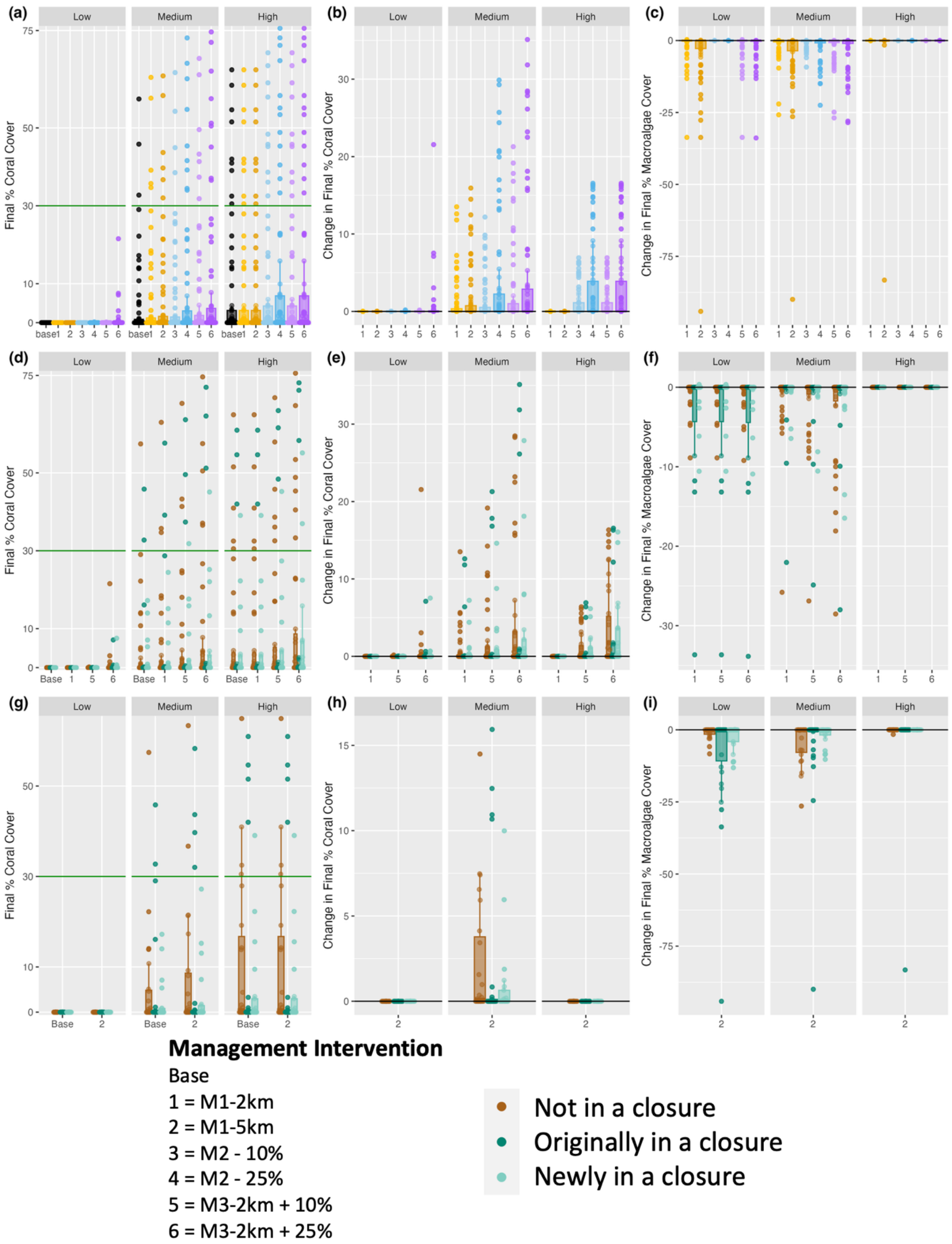

Figure S3. *Effects of the Management Interventions When d_{med} = 0.36* - Each panel shows the effect of the management intervention on the final coral cover of each reef, while each panel

shows the effect of the management under each grazing scenario. (d, e, f) Effects of fishery closure management interventions bound by fishing ground restrictions; the reefs are separated by management status under the M1, M3 management interventions, with the teal dots representing reefs that are only under fishery closure protection when the fishery closure is extended and the light blue dots representing reefs that were originally and remain under fishery closure protection under all the interventions and the baseline simulation. (g, h, i) Effects of fishery closure management intervention not bound by fishing ground restrictions; the reefs are separated by management status under the 5km fishery closure increase management intervention (i.e., M1-5km), with the teal dots representing reefs that are only under fishery closure protection when the fishery closure is extended to ~5km (i.e., M1- 5km) and the light blue dots representing reefs that were originally and remain under fishery closure protection under all the interventions and the baseline simulation. (a, d, g) Final percent coral cover in each reef, with a green line at 30% indicating a healthy reef (Birrell et al., 2020; WCS 2022). (b, e, h) Difference in the percent coral cover in each reef between each management intervention and the baseline simulation; the black line at 0 indicates the reefs that went through no change in percent coral cover. (c, f, i) Difference in the percent macroalgal cover in each reef between each management intervention and the baseline simulation; the black line at 0 indicates the reefs that went through no change in percent macroalgal cover. 'Base' represents the baseline simulations with no modeled management intervention. Each point represents the final % coral cover of a particular reef and box plots showing the inter-quartile range of the values are placed behind the points to indicate spread; in (a, d, g) the points are jittered along the *x*-axis to make it easier to distinguish individual points.

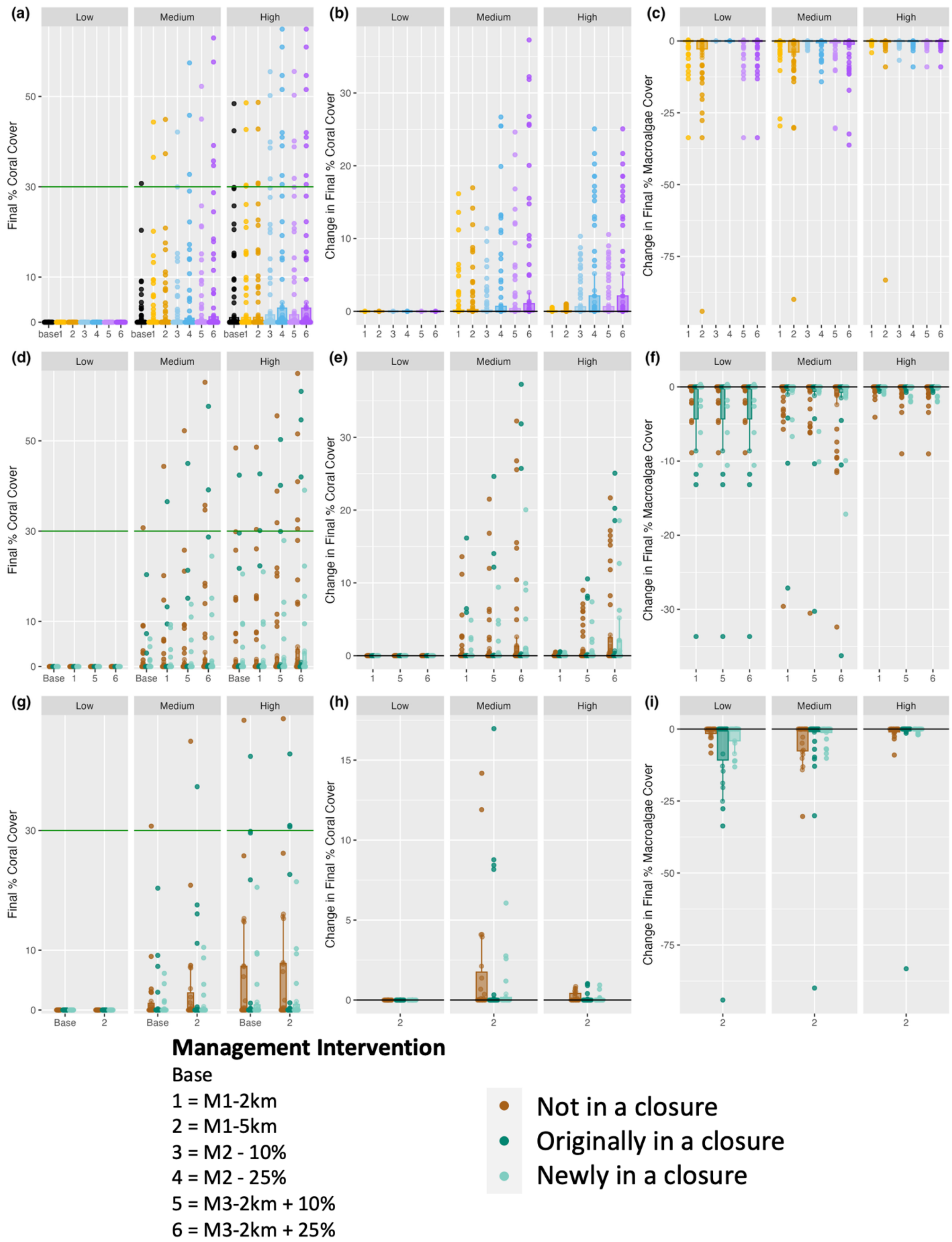
(a)
(b)
(c)
(d)
(e)
(f)
(g)
(h)
(i)
Low
Medium
High
Final % Coral Cover
Change in Final % Coral Cover
Change in Final % Macroalgae Cover
Management Intervention
Base
1 = M1-2km
2 = M1-5km
3 = M2 - 10%
4 = M2 - 25%
5 = M3-2km + 10%
6 = M3-2km + 25%
Not in a closure
Originally in a closure
Newly in a closure

Figure S4. *Effects of the Management Interventions When d_{med} = 0.48* - Each panel shows the effect of the management intervention on the final coral cover of each reef, while each panel shows the effect of the management under each grazing scenario. (d, e, f) Effects of fishery closure management interventions bound by fishing ground restrictions; the reefs are separated by management status under the M1, M3 management interventions, with the teal dots representing reefs that are only under fishery closure protection when the fishery closure is extended and the light blue dots representing reefs that were originally and remain under fishery closure protection under all the interventions and the baseline simulation. (g, h, i) Effects of fishery closure management intervention not bound by fishing ground restrictions; the reefs are separated by management status under the 5km fishery closure increase management intervention (i.e., M1-5km), with the teal dots representing reefs that are only under fishery closure protection when the fishery closure is extended to ~5km (i.e., M1- 5km) and the light blue dots representing reefs that were originally and remain under fishery closure protection under all the interventions and the baseline simulation. (a, d, g) Final percent coral cover in each reef, with a green line at 30% indicating a healthy reef (Birrell et al., 2020; WCS 2022). (b, e, h) Difference in the percent coral cover in each reef between each management intervention and the baseline simulation; the black line at 0 indicates the reefs that went through no change in percent coral cover. (c, f, i) Difference in the percent macroalgal cover in each reef between each management intervention and the baseline simulation; the black line at 0 indicates the reefs that went through no change in percent macroalgal cover. 'Base' represents the baseline simulations with no modeled management intervention. Each point represents the final % coral cover of a particular reef and box plots showing the inter-quartile range of the values are placed behind the points to indicate spread; in (a, d, g) the points are jittered along the *x*-axis to make it easier to distinguish individual points.

Rate of Overgrowth of Mature Macroalgae over Mature Coral Sensitivity Analysis

As discussed in Appendix S3, the rate of overgrowth of mature macroalgae over mature coral was set to be the same value for all 75 reefs (a = 0.1), which came from single-reef and two-reef versions (Elmhirst et al., 2009; Mumby et al., 2007; Greiner et al., 2022) of the 75-Reef Fiji Model in the absence of any knowledge of the empirical value of this parameter in any of the reefs. To assess the sensitivity of the management results to this choice of a, we ran the 75-Reef Fiji Model under various values of a (0.05, 0.07, 0.15, 0.2) chosen to span a similar range of values used in the literature for the coral mortality parameter in coral reef benthic cover models (Blackwood et al., 2012; Fung et al., 2011; Fabina et al., 2015) while also using reasonable increments (multiplying a = 0.1 by 1/2, 2/3, 3/2, 2 respectively; same as those used for the sensitivity analysis of d_{med} above).

Overall, varying the rate of overgrowth of mature macroalgae over mature coral did not alter the relative effectiveness of the different management interventions but it did change the final benthic cover of the reefs (Table S4, Figures S5-8). For the values of the rate of overgrowth of mature macroalgae over mature coral that we assessed, the trends that we observed in final benthic cover under each management intervention remained the same (e.g. M1 interventions

more effective at decreasing final macroalgal cover but less effective at increasing final coral cover than the M2 interventions; increasing the magnitude of the intervention led to a larger increase in final coral cover) (Figures S5-8a-c,). We also found that the M1 and M3 interventions could still increase the final coral cover of reefs and decrease the final macroalgal cover outside and inside of fishery closures (Figures S5-8d-i). The results of the expanded EVPI analysis, which included all the grazing scenarios in conjunction with the rates of overgrowth of mature macroalgae over mature coral, were also unchanged (EVPI value of 0; Table S4).

Table S3: *Overall Scenarios* - This table describes all of the overall scenarios (Grazing*a Scenarios) shown in Table S4 below.

Overall Scenario	Grazing Scenario	a value
1	Low	0.05
2	Medium	0.05
3	High	0.05
4	Low	0.07
5	Medium	0.07
6	High	0.07
7	Low	0.1
8	Medium	0.1
9	High	0.1
10	Low	0.15
11	Medium	0.15
12	High	0.15
13	Low	0.2
14	Medium	0.2
15	High	0.2

Table S4: *Expected Value of Perfect Information* - The numbers in the table below represent the number of reefs under each management intervention and grazing scenario whose final percent coral cover is >30%.

Overall Scenario/ Management Intervention	1	2	3	4	5	6	7	8	9	10	11	12	13	14	15	Avg across scenarios
1. M1-2km	1	12	12	1	12	12	**1**	11	12	**1**	9	12	0	9	12	7.75

2. M1-5km	1	12	12	1	12	12	**1**	12	12	**1**	11	12	0	11	12	8.08
3. M2-10%	1	12	14	1	12	14	0	12	14	**1**	12	14	0	9	14	8.58
4. M2-25%	1	**16**	**16**	1	**16**	**16**	**1**	13	**16**	**1**	**13**	**16**	**1**	**13**	**16**	10.5
5. M3-2km+10%	1	12	14	1	12	14	**1**	12	14	**1**	12	14	**1**	11	14	8.92
6. M3-2km+25%	**2**	**16**	**16**	**2**	**16**	**16**	**1**	**15**	**16**	**1**	**13**	**16**	**1**	**13**	**16**	*10.67*
Avg across management interventions	1	13	13.71	1	13	13.71	0.83	12.5	14	0.71	11.29	13.71	0.43	10.57	13.71	

Note: The numbers in **bold** represent the highest values in each column (i.e. the management intervention that results in the highest number of reefs with a percent coral cover >30% under that grazing scenario) and the value in *italics* (10.67) is the highest average number of reefs with percent coral cover >30% across all management interventions (averaged across the scenarios). Taking the average of the highest value in each of the three middle columns (i.e. the numbers in **bold** (i.e. 2+16+16+2+16+16+1+15+16+1+13+16+1+13+16)/15 = 10.67)) and then subtracting the highest value in the final column (i.e. the number in *italics*: 10.67) gives you the EVPI value for this analysis (0).

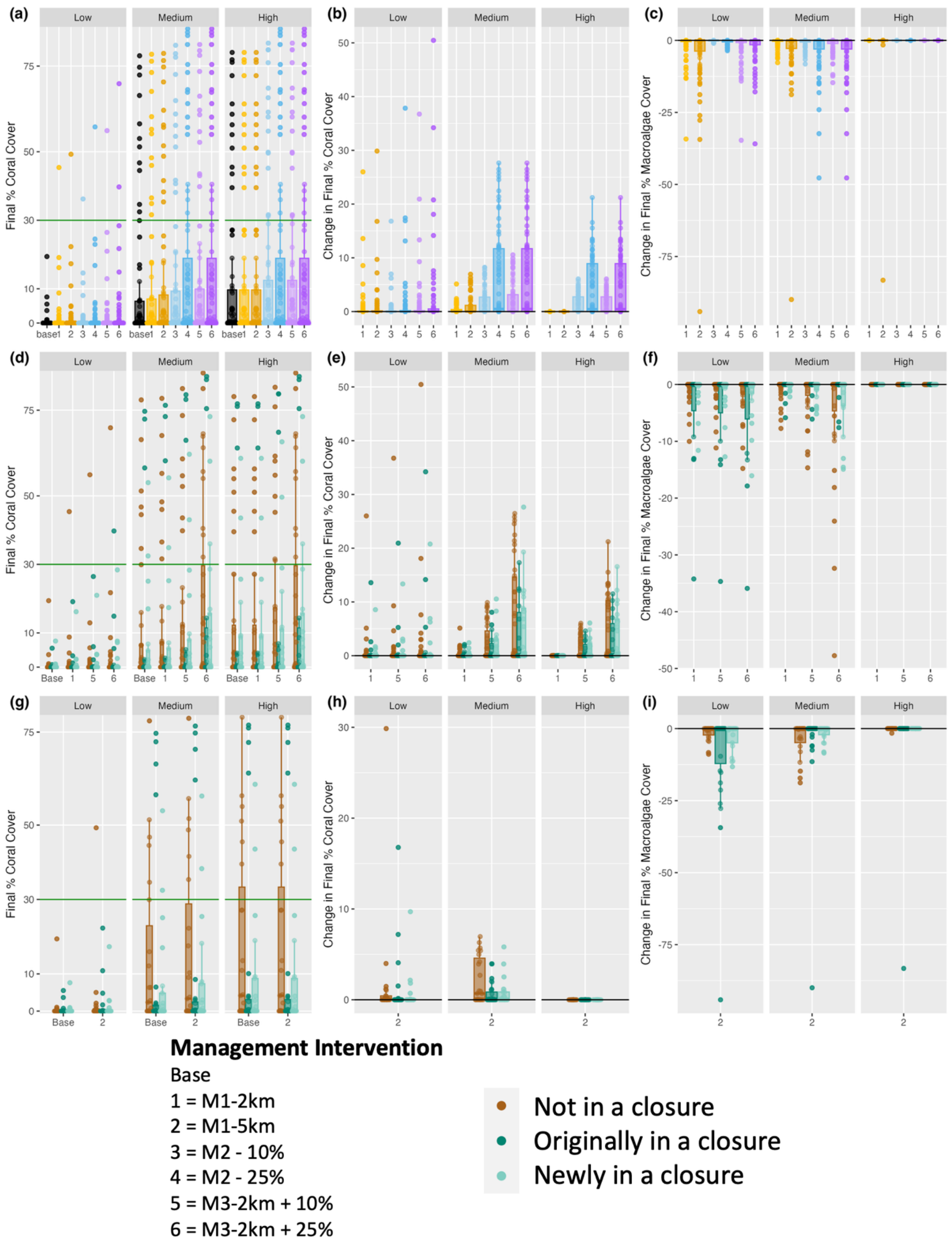

(a)
(b)
(c)
(d)
(e)
(f)
(g)
(h)
(i)
Low
Medium
High
Final % Coral Cover
Change in Final % Coral Cover
Change in Final % Macroalgae Cover
Management Intervention
Base
1 = M1-2km
2 = M1-5km
3 = M2 - 10%
4 = M2 - 25%
5 = M3-2km + 10%
6 = M3-2km + 25%
Not in a closure
Originally in a closure
Newly in a closure

Figure S5. *Effects of the Management Interventions When a = 0.05* - Each panel shows the effect of the management intervention on the final coral cover of each reef, while each panel shows the effect of the management under each grazing scenario. (d, e, f) Effects of fishery closure management interventions bound by fishing ground restrictions; the reefs are separated by management status under the M1, M3 management interventions, with the teal dots representing reefs that are only under fishery closure protection when the fishery closure is extended and the light blue dots representing reefs that were originally and remain under fishery closure protection under all the interventions and the baseline simulation. (g, h, i) Effects of fishery closure management intervention not bound by fishing ground restrictions; the reefs are separated by management status under the 5km fishery closure increase management intervention (i.e., M1-5km), with the teal dots representing reefs that are only under fishery closure protection when the fishery closure is extended to ~5km (i.e., M1- 5km) and the light blue dots representing reefs that were originally and remain under fishery closure protection under all the interventions and the baseline simulation. (a, d, g) Final percent coral cover in each reef, with a green line at 30% indicating a healthy reef (Birrell et al., 2020; WCS 2022). (b, e, h) Difference in the percent coral cover in each reef between each management intervention and the baseline simulation; the black line at 0 indicates the reefs that went through no change in percent coral cover. (c, f, i) Difference in the percent macroalgal cover in each reef between each management intervention and the baseline simulation; the black line at 0 indicates the reefs that went through no change in percent macroalgal cover. 'Base' represents the baseline simulations with no modeled management intervention. Each point represents the final % coral cover of a particular reef and box plots showing the inter-quartile range of the values are placed behind the points to indicate spread; in (a, d, g) the points are jittered along the *x*-axis to make it easier to distinguish individual points.

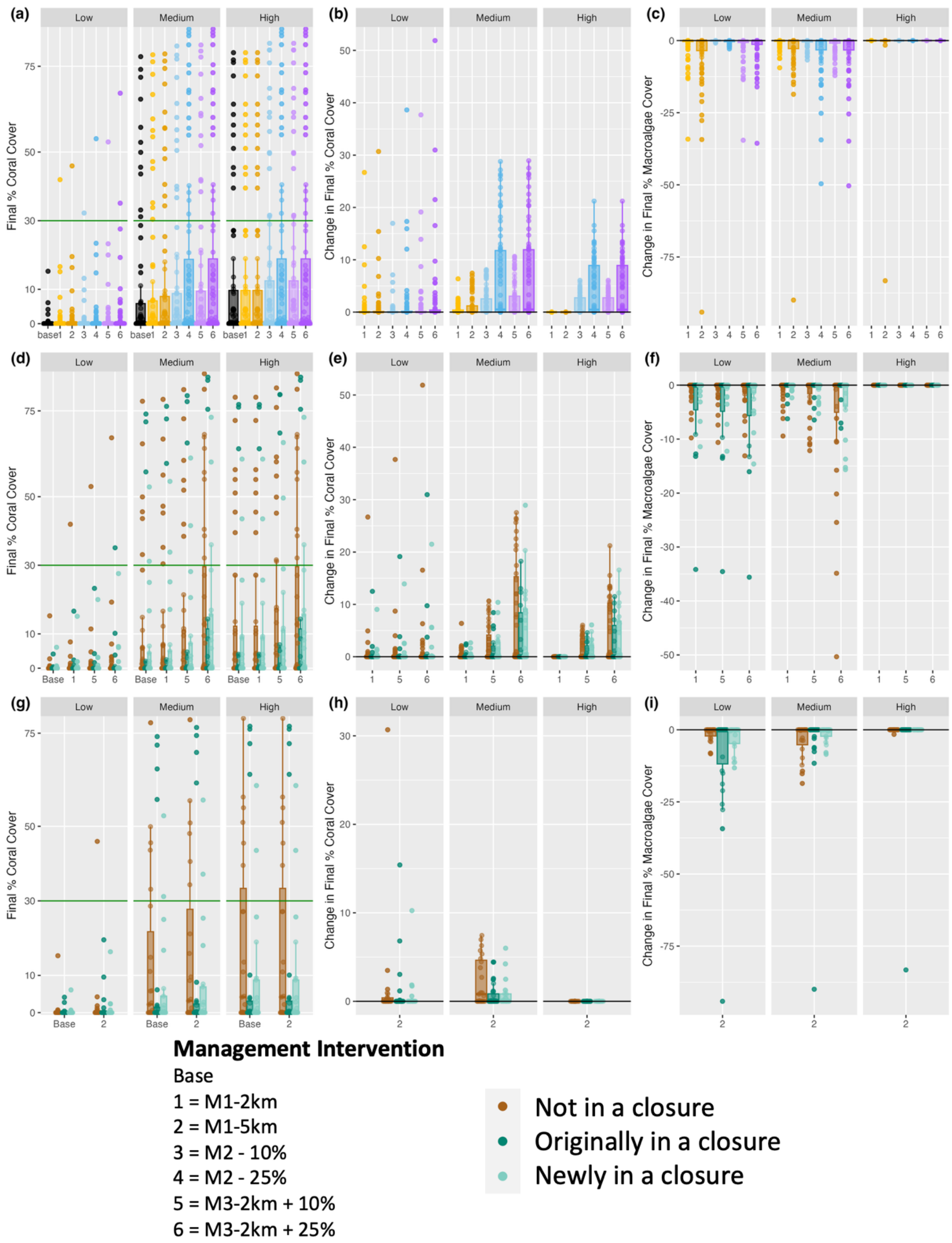

Figure S6. *Effects of the Management Interventions When a = 0.07* - Each panel shows the effect of the management intervention on the final coral cover of each reef, while each panel shows the

effect of the management under each grazing scenario. (d, e, f) Effects of fishery closure management interventions bound by fishing ground restrictions; the reefs are separated by management status under the M1, M3 management interventions, with the teal dots representing reefs that are only under fishery closure protection when the fishery closure is extended and the light blue dots representing reefs that were originally and remain under fishery closure protection under all the interventions and the baseline simulation. (g, h, i) Effects of fishery closure management intervention not bound by fishing ground restrictions; the reefs are separated by management status under the 5km fishery closure increase management intervention (i.e., M1-5km), with the teal dots representing reefs that are only under fishery closure protection when the fishery closure is extended to ~5km (i.e., M1- 5km) and the light blue dots representing reefs that were originally and remain under fishery closure protection under all the interventions and the baseline simulation. (a, d, g) Final percent coral cover in each reef, with a green line at 30% indicating a healthy reef (Birrell et al., 2020; WCS 2022). (b, e, h) Difference in the percent coral cover in each reef between each management intervention and the baseline simulation; the black line at 0 indicates the reefs that went through no change in percent coral cover. (c, f, i) Difference in the percent macroalgal cover in each reef between each management intervention and the baseline simulation; the black line at 0 indicates the reefs that went through no change in percent macroalgal cover. 'Base' represents the baseline simulations with no modeled management intervention. Each point represents the final % coral cover of a particular reef and box plots showing the inter-quartile range of the values are placed behind the points to indicate spread; in (a, d, g) the points are jittered along the *x*-axis to make it easier to distinguish individual points.

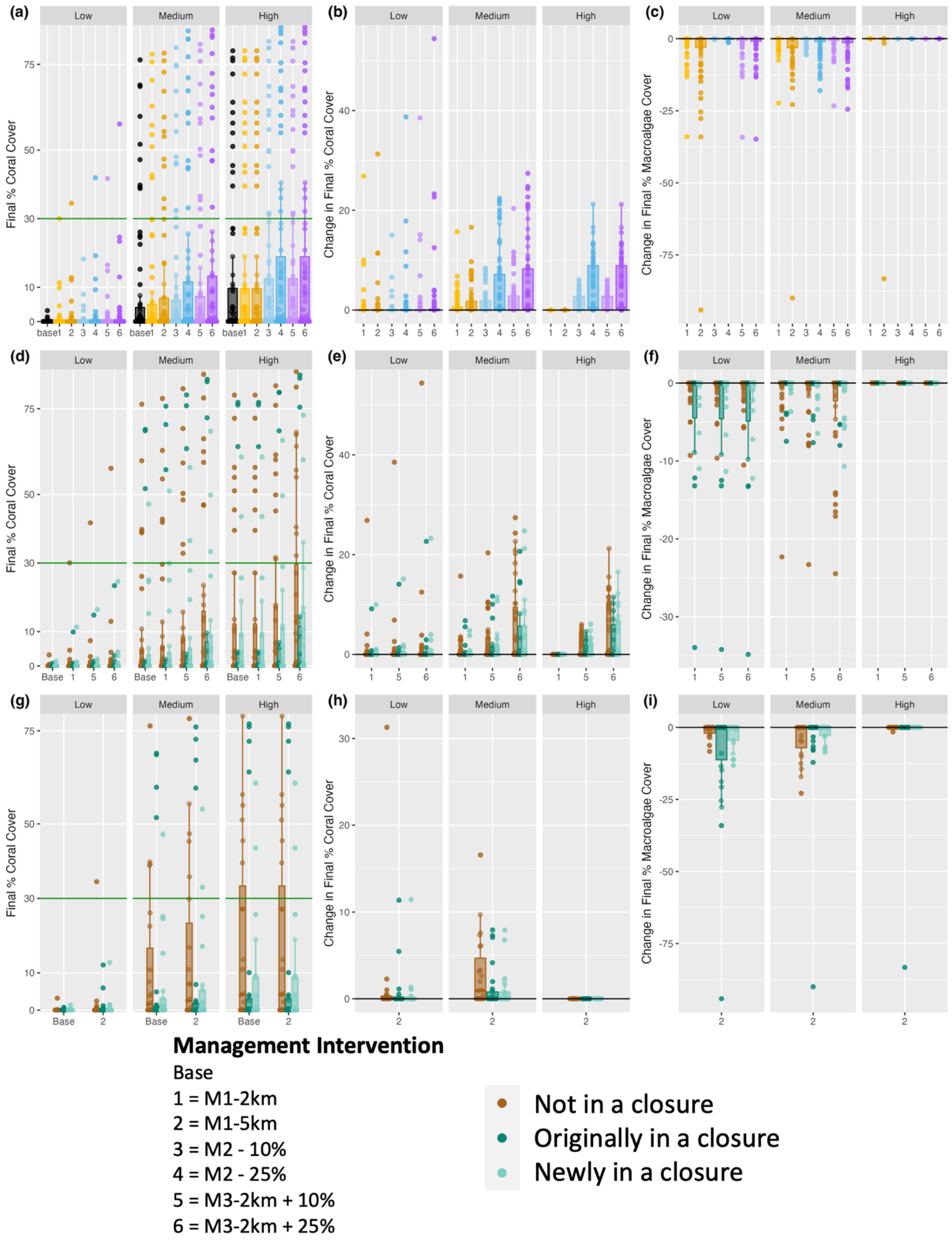

Figure S7. *Effects of the Management Interventions When a = 0.15* - Each panel shows the effect of the management intervention on the final coral cover of each reef, while each panel shows the

effect of the management under each grazing scenario. (d, e, f) Effects of fishery closure management interventions bound by fishing ground restrictions; the reefs are separated by management status under the M1, M3 management interventions, with the teal dots representing reefs that are only under fishery closure protection when the fishery closure is extended and the light blue dots representing reefs that were originally and remain under fishery closure protection under all the interventions and the baseline simulation. (g, h, i) Effects of fishery closure management intervention not bound by fishing ground restrictions; the reefs are separated by management status under the 5km fishery closure increase management intervention (i.e., M1-5km), with the teal dots representing reefs that are only under fishery closure protection when the fishery closure is extended to ~5km (i.e., M1- 5km) and the light blue dots representing reefs that were originally and remain under fishery closure protection under all the interventions and the baseline simulation. (a, d, g) Final percent coral cover in each reef, with a green line at 30% indicating a healthy reef (Birrell et al., 2020; WCS 2022). (b, e, h) Difference in the percent coral cover in each reef between each management intervention and the baseline simulation; the black line at 0 indicates the reefs that went through no change in percent coral cover. (c, f, i) Difference in the percent macroalgal cover in each reef between each management intervention and the baseline simulation; the black line at 0 indicates the reefs that went through no change in percent macroalgal cover. 'Base' represents the baseline simulations with no modeled management intervention. Each point represents the final % coral cover of a particular reef and box plots showing the inter-quartile range of the values are placed behind the points to indicate spread; in (a, d, g) the points are jittered along the *x*-axis to make it easier to distinguish individual points.

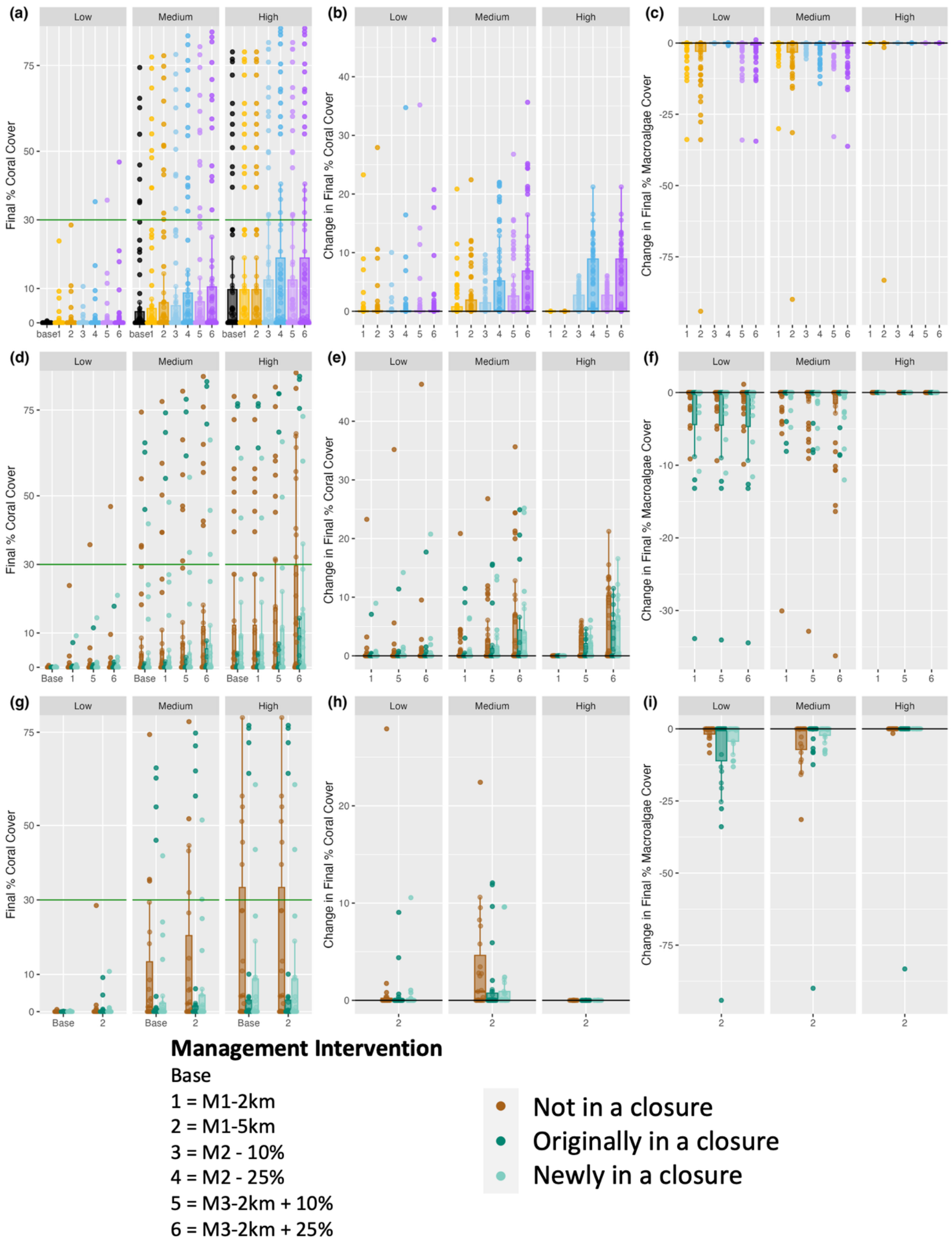

Figure S8. *Effects of the Management Interventions When a = 0.2* - Each panel shows the effect of the management intervention on the final coral cover of each reef, while each panel shows the

effect of the management under each grazing scenario. (d, e, f) Effects of fishery closure management interventions bound by fishing ground restrictions; the reefs are separated by management status under the M1, M3 management interventions, with the teal dots representing reefs that are only under fishery closure protection when the fishery closure is extended and the light blue dots representing reefs that were originally and remain under fishery closure protection under all the interventions and the baseline simulation. (g, h, i) Effects of fishery closure management intervention not bound by fishing ground restrictions; the reefs are separated by management status under the 5km fishery closure increase management intervention (i.e., M1-5km), with the teal dots representing reefs that are only under fishery closure protection when the fishery closure is extended to ~5km (i.e., M1- 5km) and the light blue dots representing reefs that were originally and remain under fishery closure protection under all the interventions and the baseline simulation. (a, d, g) Final percent coral cover in each reef, with a green line at 30% indicating a healthy reef (Birrell et al., 2020; WCS 2022). (b, e, h) Difference in the percent coral cover in each reef between each management intervention and the baseline simulation; the black line at 0 indicates the reefs that went through no change in percent coral cover. (c, f, i) Difference in the percent macroalgal cover in each reef between each management intervention and the baseline simulation; the black line at 0 indicates the reefs that went through no change in percent macroalgal cover. 'Base' represents the baseline simulations with no modeled management intervention. Each point represents the final % coral cover of a particular reef; in (a, d, g) the points are jittered along the *x*-axis to make it easier to distinguish individual points.

Coral Larval Dispersal Sensitivity Analysis

We performed a sensitivity analysis to assess how sensitive our findings were to the modeled coral larval dispersal probabilities, as (1) we anticipate that these may change into the future (Figueiredo et al., 2014; O'Connor et al., 2007), (2) they are understudied in this region, (3) we had a known range of coral pelagic larval durations (PLD) to assess, from past studies that used empirically-derived coral larval dispersal values (Wood et al., 2014 (1-120 days PLD); Treml et al., 2015 (10-60 days PLD); Hock et al., 2017 (7-14 days PLD); Gamoyo et al., 2019 (15-60 days PLD)), and (4) little is known about how varying the coral larval dispersal probability in a complex network will alter the dynamics of the system (as previous studies such as Fabina et al., 2015 and Fung et al., 2011 that used similar equations each modeled a single reef patch).

To determine the sensitivity of our results to varying the coral larval dispersal rate (k_{ij}), we varied the pelagic larval duration (PLD) used to generate the coral larval connectivity matrix in the 75-Reef Fiji Model and assessed whether it impacted the effectiveness of the different management methods that we modeled. We ran the 75-Reef Fiji Model with coral larval connectivity matrices obtained using pelagic larval durations (PLD) ranging from 10 to 130 days in steps of 5 days. When we ran the simulation under some coral larval PLDs (PLD 20,25,35,40,45,50,60,70,75,80,85,90,95,100,105,110,115,120,125,130), the final coral cover of all of the reefs was 0% under all three grazing scenarios. For the sake of space and clarity, we only report the simulation results of the PLDs that resulted in at least one reef with >30% final

coral cover (Birrell et al. 2020) in at least one grazing scenario under at least one management intervention or under baseline conditions (i.e. PLD = 10,15,30,55,65; see Table S1 below for a full list of all of the scenarios presented in the figures and tables below). We ignore the cases of 0 coral persistence because they do not tell us anything about management effectiveness and are likely the result of a model artifact, resulting from the majority of the larvae leaving our artificially constrained system (only 75 reefs of a much bigger reef network; connectivity matrices with PLD >65 all had this problem)*. Note that under PLD 30, 55, and 65, we only see one reef that has a positive final coral cover. It is therefore difficult to assess whether or not the fishery closure management methods (M1, see Table 1 in the main text) really do increase the coral cover of all of the reefs, not just those newly in closures. However, under each of these PLDs (see Table S2 and Figure S3-5), the single reef with a positive final coral cover under the baseline conditions is the same single reef that increases in final coral cover under M1 managements (Table 1). This indicates that said single reef under each of those PLDs has a positive coral cover for reasons independent of the management method chosen, and thus the fact that said single reef under PLD 30 and PLD 55 is a 'Newly in a Closure' reef and a 'Not in a Closure' reef under PLD 65 is due to the amount of coral larval dispersal those reefs experience.

Overall, we find that varying the coral PLD did not alter the relative effectiveness of the different management interventions but it did change the final benthic cover of the reefs (Table S6, Figures S9-13). For the relevant connectivity matrices (i.e. the connectivity matrices that resulted in non-zero final coral cover values) that we assessed, the trends that we observed in final benthic cover under each management intervention remained the same (e.g. M1 interventions more effective at decreasing final macroalgal cover but less effective at increasing final coral cover than the M2 interventions; increasing the magnitude of the intervention led to a larger increase in final coral cover) (Figures S9-13a-c,). We also found that the M1 and M3 interventions could still increase the final coral cover of reefs and decrease the final macroalgal cover outside and inside of fishery closures (Figures S9-13d-i). The results of the expanded EVPI analysis, which included all of the grazing scenarios in conjunction with the relevant connectivity matrices, were also unchanged (EVPI value of 0; Table S6).

Table S5: *Overall Scenarios* - This table describes all of the overall scenarios (Grazing*Connectivity Matrix Scenarios) shown in Table S6 below.

Overall Scenario	Grazing Scenario	Coral Larval Connectivity Matrix
1	Low	10
2	Medium	10
3	High	10
4	Low	15
5	Medium	15
6	High	15

7	Low	30
8	Medium	30
9	High	30
10	Low	55
11	Medium	55
12	High	55
13	Low	65
14	Medium	65
15	High	65
16	Low	Weighted Average
17	Medium	Weighted Average
18	High	Weighted Average

Table S6: *Expected Value of Perfect Information* - The numbers in the table below represent the number of reefs under each management intervention and grazing scenario whose final percent coral cover is >30%.

**Overall Scenario/ Management Intervention**	**1**	**2**	**3**	**4**	**5**	**6**	**7**	**8**	**9**	**10**	**11**	**12**	**13**	**14**	**15**	**16**	**17**	**18**	**Avg across scenarios**
1. M1-2km	1	**13**	**13**	2	**5**	**5**	**0**	0	**1**	**0**	0	**1**	**0**	**1**	**1**	**1**	11	12	3.72
2. M1-5km	1	**13**	**13**	2	**5**	**5**	**0**	0	**1**	**0**	0	**1**	**0**	**1**	**1**	**1**	12	12	3.78

3. M2-10%	1	**13**	**13**	2	**5**	**5**	**0**	0	**1**	**0**	0	**1**	**0**	**1**	**1**	0	12	14	3.83
4. M2-25%	1	**13**	**13**	**3**	**5**	**5**	**0**	**1**	**1**	**0**	**1**	**1**	**0**	**1**	**1**	**1**	13	**16**	4.22
5. M3-2km + 10%	1	**13**	**13**	**3**	**5**	**5**	**0**	0	**1**	**0**	**1**	**1**	**0**	**1**	**1**	**1**	12	14	4
6. M3-2km + 25%	**3**	**13**	**13**	**3**	**5**	**5**	**0**	**1**	**1**	**0**	**1**	**1**	**0**	**1**	**1**	**1**	**15**	**16**	*4.44*
Average across Management Interventions	1.33	13	13	2.5	5	5	0	0.33	1	0	0.5	1	0	1	1	0.83	12.5	14	

Note: The numbers in **bold** represent the highest values in each column (i.e. the management intervention that results in the highest number of reefs with a percent coral cover >30% under that grazing scenario) and the value in *italics* (4.44) is the highest average number of reefs with percent coral cover >30% across all management interventions (averaged across the scenarios). Taking the average of the highest value in each of the three middle columns (i.e. the numbers in **bold** (i.e. (3+13+13+1+15+16+3+5+5+0+1+1+0+1+1+0+1+1)/18 = 4.44)) and then subtracting the highest value in the final column (i.e. the number in *italics*: 4.44) gives you the EVPI value for this analysis (0).

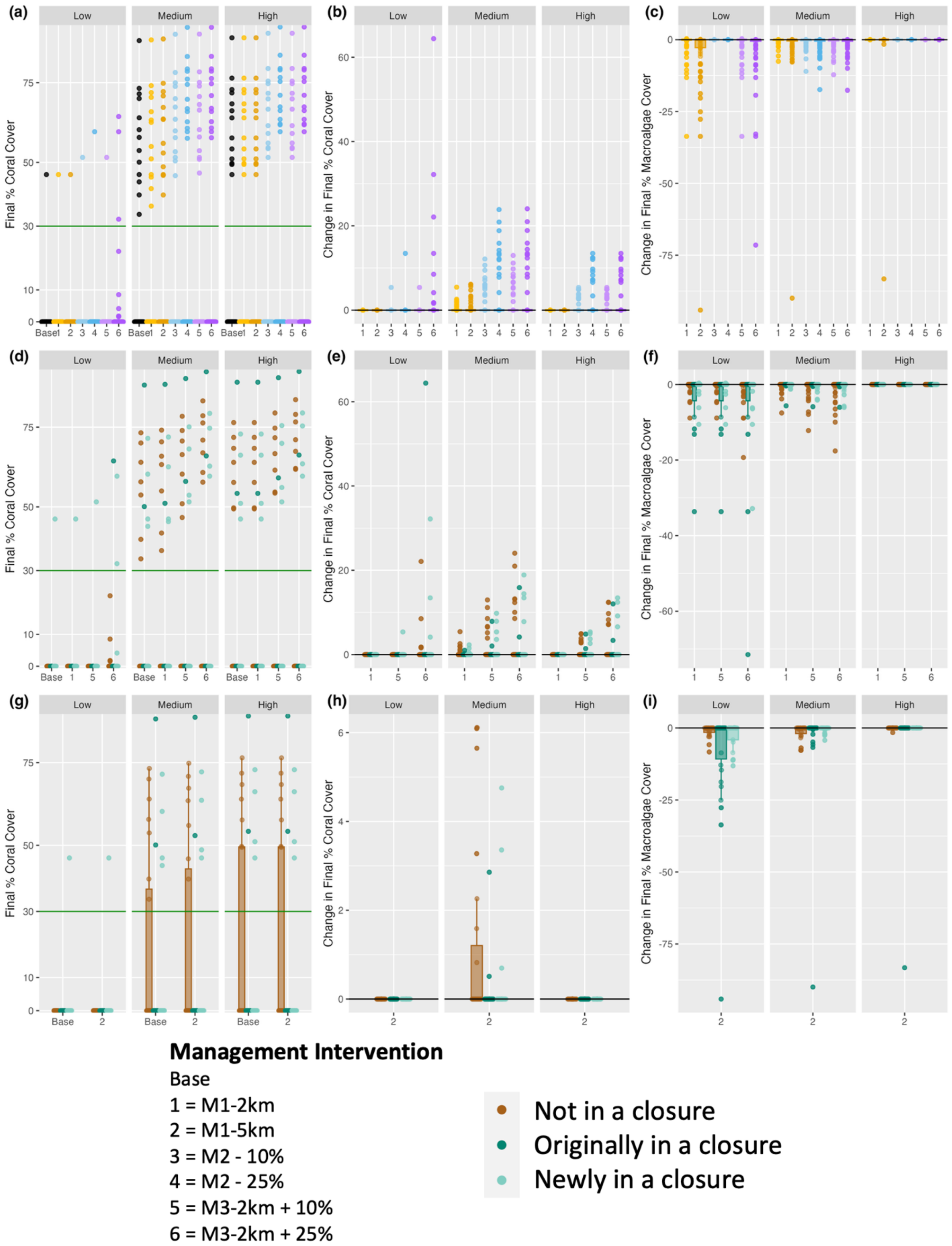

(a)
(b)
(c)
(d)
(e)
(f)
(g)
(h)
(i)
Low
Medium
High
Final % Coral Cover
Change in Final % Coral Cover
Change in Final % Macroalgae Cover
Base
Management Intervention
Base
1 = M1-2km
2 = M1-5km
3 = M2 - 10%
4 = M2 - 25%
5 = M3-2km + 10%
6 = M3-2km + 25%
Not in a closure
Originally in a closure
Newly in a closure

Figure S9. *Effects of the Management Interventions Under the PLD 10 Coral Larval Connectivity Matrix* - Each panel shows the effect of the management intervention on the final coral cover of each reef, while each panel shows the effect of the management under each grazing scenario. In panels (d)-(f), the reefs are separated by management status under the M1, M3 management interventions, with the teal dots representing reefs that are only under fishery closure protection when the fishery closure is extended and the light blue dots representing reefs that were originally and remain under fishery closure protection under all the interventions and the baseline simulation. In panels (g)-(i), the reefs are separated by management status under the 5km fishery closure increase management intervention (i.e., M1-5km), with the teal dots representing reefs that are only under fishery closure protection when the fishery closure is extended to ~5km (i.e., M1- 5km) and the light blue dots representing reefs that were originally and remain under fishery closure protection under all the interventions and the baseline simulation. (a, d, g) Final percent coral cover in each reef, with a green line at 30% indicating a healthy reef (Birrell et al., 2020; WCS 2022). (b, e, h) Difference in the percent coral cover in each reef between each management intervention and the baseline simulation; the black line at 0 indicates the reefs that went through no change in percent coral cover. (c, f, i) Difference in the percent macroalgal cover in each reef between each management intervention and the baseline simulation; the black line at 0 indicates the reefs that went through no change in percent macroalgal cover. 'Base' represents the baseline simulations with no modeled management intervention. Each point represents the final % coral cover of a particular reef and box plots showing the inter-quartile range of the values are placed behind the points to indicate spread; in (a, d, g) the points are jittered along the *x*-axis to make it easier to distinguish individual points.

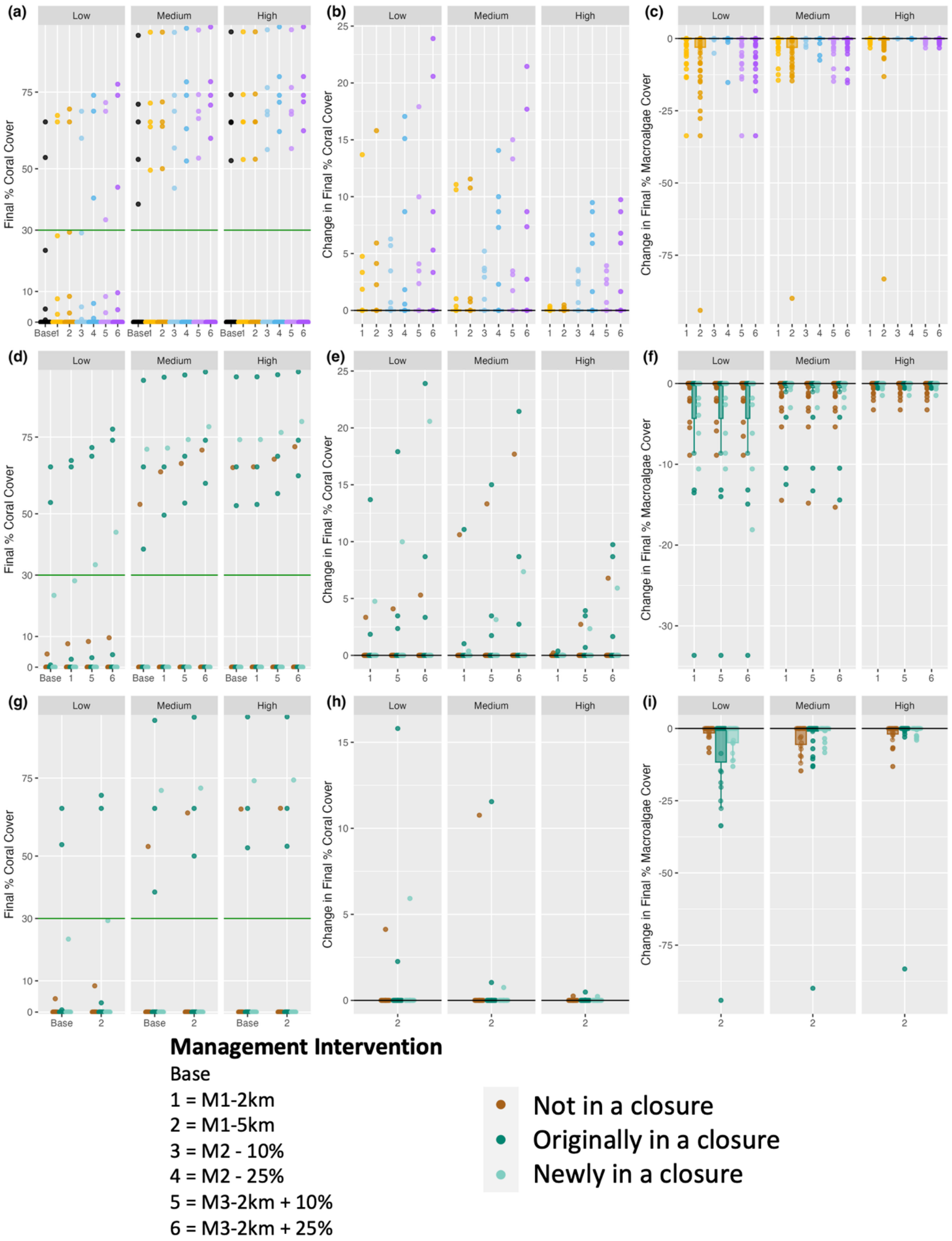

Figure S10. *Effects of the Management Interventions Under the PLD 15 Coral Larval Connectivity Matrix* - Each panel shows the effect of the management intervention on the final

coral cover of each reef, while each panel shows the effect of the management under each grazing scenario. In panels (d)-(f), the reefs are separated by management status under the M1, M3 management interventions, with the teal dots representing reefs that are only under fishery closure protection when the fishery closure is extended and the light blue dots representing reefs that were originally and remain under fishery closure protection under all the interventions and the baseline simulation. In panels (g)-(i), the reefs are separated by management status under the 5km fishery closure increase management intervention (i.e., M1-5km), with the teal dots representing reefs that are only under fishery closure protection when the fishery closure is extended to ~5km (i.e., M1- 5km) and the light blue dots representing reefs that were originally and remain under fishery closure protection under all the interventions and the baseline simulation. (a, d, g) Final percent coral cover in each reef, with a green line at 30% indicating a healthy reef (Birrell et al., 2020; WCS 2022). (b, e, h) Difference in the percent coral cover in each reef between each management intervention and the baseline simulation; the black line at 0 indicates the reefs that went through no change in percent coral cover. (c, f, i) Difference in the percent macroalgal cover in each reef between each management intervention and the baseline simulation; the black line at 0 indicates the reefs that went through no change in percent macroalgal cover. 'Base' represents the baseline simulations with no modeled management intervention. Each point represents the final % coral cover of a particular reef and box plots showing the inter-quartile range of the values are placed behind the points to indicate spread; in (a, d, g) the points are jittered along the *x*-axis to make it easier to distinguish individual points.

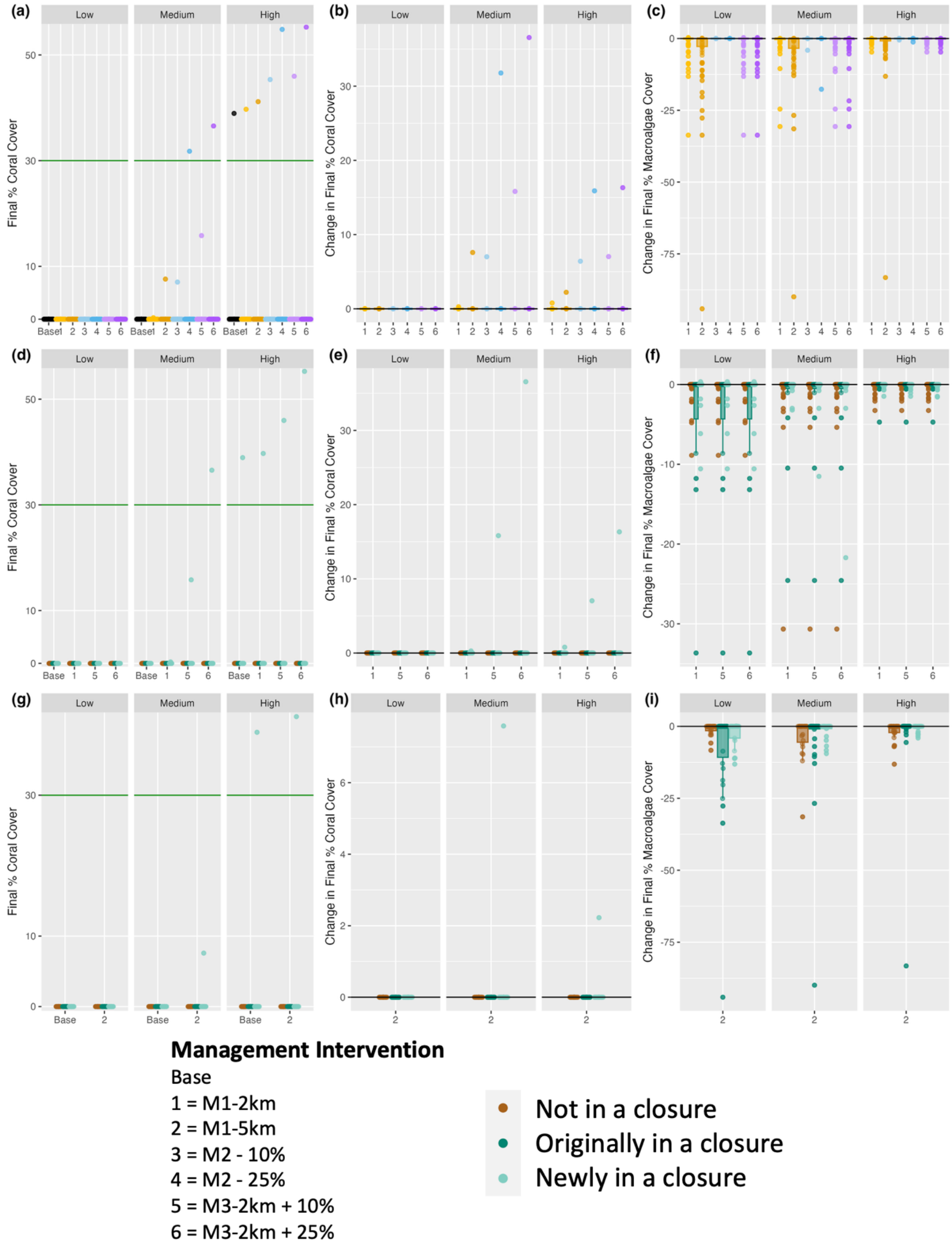

Figure S11. *Effects of the Management Interventions Under the PLD 30 Coral Larval Connectivity Matrix* - Each panel shows the effect of the management intervention on the final

coral cover of each reef, while each panel shows the effect of the management under each grazing scenario. In panels (d)-(f), the reefs are separated by management status under the M1, M3 management interventions, with the teal dots representing reefs that are only under fishery closure protection when the fishery closure is extended and the light blue dots representing reefs that were originally and remain under fishery closure protection under all the interventions and the baseline simulation. In panels (g)-(i), the reefs are separated by management status under the 5km fishery closure increase management intervention (i.e., M1-5km), with the teal dots representing reefs that are only under fishery closure protection when the fishery closure is extended to ~5km (i.e., M1- 5km) and the light blue dots representing reefs that were originally and remain under fishery closure protection under all the interventions and the baseline simulation. (a, d, g) Final percent coral cover in each reef, with a green line at 30% indicating a healthy reef (Birrell et al., 2020; WCS 2022). (b, e, h) Difference in the percent coral cover in each reef between each management intervention and the baseline simulation, the black line at 0 indicates the reefs that went through no change in percent coral cover. (c, f, i) Difference in the percent macroalgal cover in each reef between each management intervention and the baseline simulation, the black line at 0 indicates the reefs that went through no change in percent macroalgal cover. 'Base' represents the baseline simulations with no modeled management intervention. Each point represents the final % coral cover of a particular reef and box plots showing the inter-quartile range of the values are placed behind the points to indicate spread; in (a, d, g) the points are jittered along the *x*-axis to make it easier to distinguish individual points.

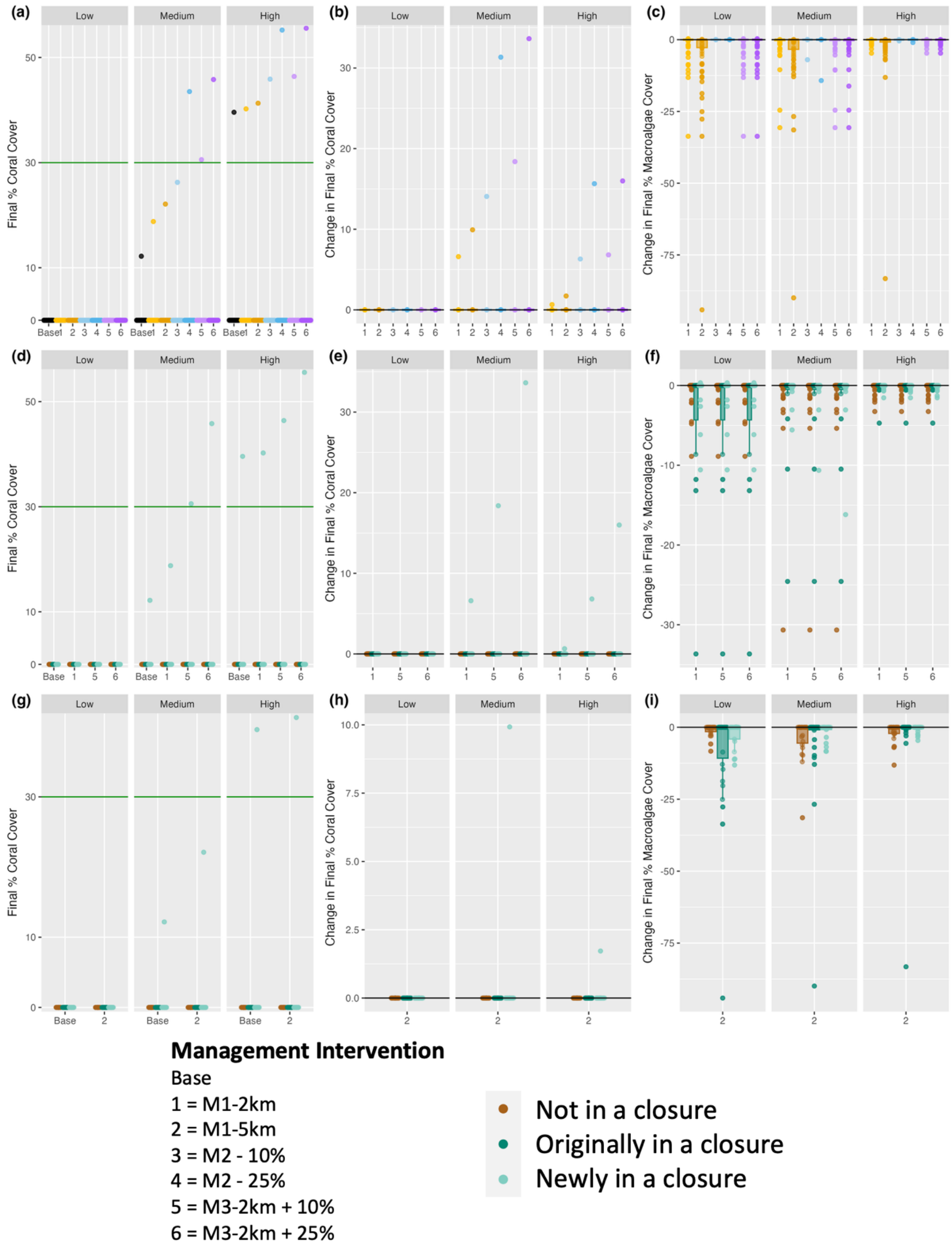

Figure S12. *Effects of the Management Interventions Under the PLD 55 Coral Larval Connectivity Matrix* - Each panel shows the effect of the management intervention on the final

coral cover of each reef, while each panel shows the effect of the management under each grazing scenario. In panels (d)-(f), the reefs are separated by management status under the M1, M3 management interventions, with the teal dots representing reefs that are only under fishery closure protection when the fishery closure is extended and the light blue dots representing reefs that were originally and remain under fishery closure protection under all the interventions and the baseline simulation. In panels (g)-(i), the reefs are separated by management status under the 5km fishery closure increase management intervention (i.e., M1-5km), with the teal dots representing reefs that are only under fishery closure protection when the fishery closure is extended to ~5km (i.e., M1- 5km) and the light blue dots representing reefs that were originally and remain under fishery closure protection under all the interventions and the baseline simulation. (a, d, g) Final percent coral cover in each reef, with a green line at 30% indicating a healthy reef (Birrell et al., 2020; WCS 2022). (b, e, h) Difference in the percent coral cover in each reef between each management intervention and the baseline simulation; the black line at 0 indicates the reefs that went through no change in percent coral cover. (c, f, i) Difference in the percent macroalgal cover in each reef between each management intervention and the baseline simulation; the black line at 0 indicates the reefs that went through no change in percent macroalgal cover. 'Base' represents the baseline simulations with no modeled management intervention. Each point represents the final % coral cover of a particular reef and box plots showing the inter-quartile range of the values are placed behind the points to indicate spread; in (a, d, g) the points are jittered along the *x*-axis to make it easier to distinguish individual points.

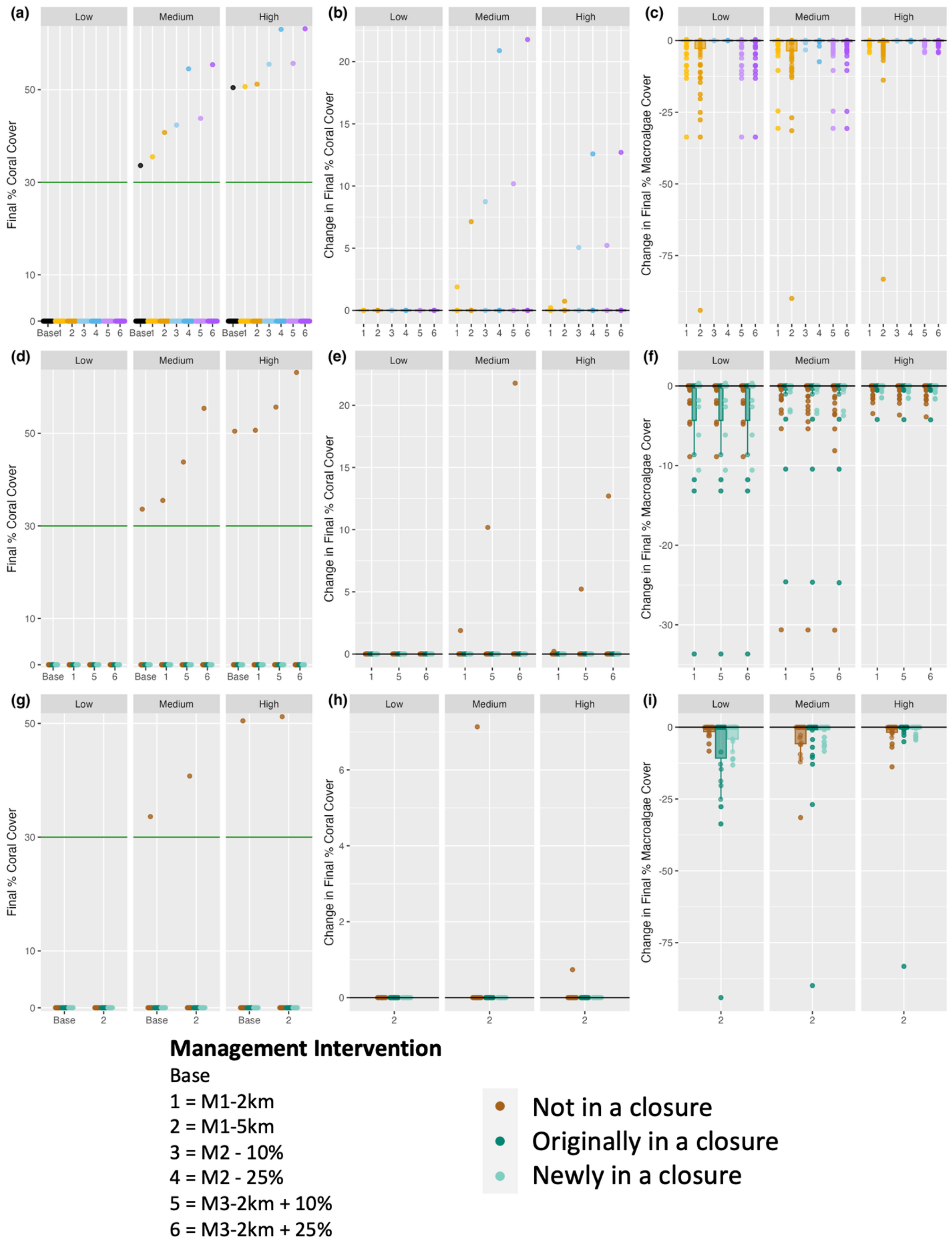

Figure S13. *Effects of the Management Interventions Under the PLD 65 Coral Larval Connectivity Matrix* - Each panel shows the effect of the management intervention on the final

coral cover of each reef, while each panel shows the effect of the management under each grazing scenario. In panels (d)-(f), the reefs are separated by management status under the M1, M3 management interventions, with the teal dots representing reefs that are only under fishery closure protection when the fishery closure is extended and the light blue dots representing reefs that were originally and remain under fishery closure protection under all the interventions and the baseline simulation. In panels (g)-(i), the reefs are separated by management status under the 5km fishery closure increase management intervention (i.e., M1-5km), with the teal dots representing reefs that are only under fishery closure protection when the fishery closure is extended to ~5km (i.e., M1- 5km) and the light blue dots representing reefs that were originally and remain under fishery closure protection under all the interventions and the baseline simulation. (a, d, g) Final percent coral cover in each reef, with a green line at 30% indicating a healthy reef (Birrell et al., 2020; WCS 2022). (b, e, h) Difference in the percent coral cover in each reef between each management intervention and the baseline simulation; the black line at 0 indicates the reefs that went through no change in percent coral cover. (c, f, i) Difference in the percent macroalgal cover in each reef between each management intervention and the baseline simulation; the black line at 0 indicates the reefs that went through no change in percent macroalgal cover. 'Base' represents the baseline simulations with no modeled management intervention. Each point represents the final % coral cover of a particular reef and box plots showing the inter-quartile range of the values are placed behind the points to indicate spread; in (a, d, g) the points are jittered along the *x*-axis to make it easier to distinguish individual points.

Table S7: *Network Structure of Coral Larval Connectivity Matrices* - This table compares the network structures that result from the different connectivity matrices used in this study. The networks described include all 551 reef sites. From the characteristics listed here, we can conclude that the structure of the PLD 10 Coral Larval Connectivity Matrix appears the closest to a 2-Regular network and the structure of the Weighted Average Coral Larval Connectivity Matrix (used to generate the results in the main text) appears the closest to a Scale-Free network (Lewis 2011).

**PLD**	**# Networks**	**# Networks (> 3 Reefs)**	**Avg Degree**	**Avg Shortest Path Length**	**Diameter**
Weighted Avg	1	1	129.29	1.70e-06	6.41e-05
10	31	2	25.49	3.20e-04	1.08e-03
15	43	4	22.56	2.91e-04	1.08e-03
30	47	1	16.159710	2.66e-04	6.67e-04
55	67	1	9.179673	3.01e-04	7.50e-04

65	86	1	8.348457	3.04e-04	7.50e-04

Note: Networks are defined as 'weak networks' (Csárdi et al., 2023) which means that each network contains all of the nodes (reef sites) that have an edge with any other node in the network. Average degree measures the mean number of nodes any node is connected to in the network. The average shortest path length is measured by recording the shortest path length between every pair of nodes in a network and then calculating the average of that. The diameter of a network is defined as the longest path length between any two nodes in a network.

*This was a problem we encountered with the mixed PLD connectivity matrix before we adjusted the gamete production rate (γ) and the larval production rate (r) to ensure coral and/or macroalgal persistence under network-wide grazing rate parameterizations known to lead to coral and/or macroalgal persistence (see Appendix S3 for more details on this). We anticipated this issue arising with some of the new connectivity matrices because we found that the model (under the mixed PLD connectivity matrix) was quite sensitive to the value of r and γ chosen, as most values that we tested of both of those variables led to a final network-wide coral cover of 0. We did not adjust r or γ because we wanted to understand the effects of shifting the coral larval dispersal rate (k_{ij}) alone, independent of the effects of also adjusting r and γ.

References

Andrello, M., E. S. Darling, A. Wenger, A. F. Suárez-Castro, S. Gelfand, and G. N. Ahmadia. 2022. "A global map of human pressures on tropical coral reefs." Conservation Letters 15:e12858.

Birrell, C. L., E. Sola, R. H. Bennett, D. van Beuningen, H. M. Costa, J. J. Sitoe, N. Sidat, S. Fernando, E.S. Darling, N.A. Muthiga and T. R. McClanahan. 2020. "A summary of WCS knowledge of the state of coral reefs in Mozambique." Wildlife Conservation Society, Maputo, Mozambique. https://biblioteca.biofund.org.mz/wp-content/uploads/2021/03/1616752045-2020_WCS_Coral_Reefs_in_Mozambique.pdf

Blackwood, J. C., A. Hastings, and P. J. Mumby. 2012. "The effect of fishing on hysteresis in Caribbean coral reefs." Theoretical Ecology 5:105–114.

Csárdi G., T. Nepusz, V. Traag, S. Horvát, F. Zanini, D. Noom, and K.Müller. 2023. "igraph: Network Analysis and Visualization in R." doi:10.5281/zenodo.7682609

Elmhirst, T., S. R. Connolly, and T. P. Hughes. 2009. Connectivity, regime shifts and the resilience of coral reefs. Coral Reefs 28:949-957.

Fabina, N. S., M. L. Baskett, and K. Gross. 2015. "The differential effects of increasing frequency and magnitude of extreme events on coral populations." Ecological Applications 25:1534-1545.

Fung, T., R. M. Seymour, and C. R. Johnson. 2011. "Alternative stable states and phase shifts in coral reefs under anthropogenic stress." Ecology 92:967-982.

Gamoyo, M., D. Obura, and C. J. C. Reason. 2019. "Estimating connectivity through larval dispersal in the Western Indian Ocean." Journal of Geophysical Research: Biogeosciences 124:2446-2459.

Greiner, A., E. S. Darling, M. -J. Fortin, and M. Krkošek. 2022. "The combined effects of dispersal and herbivores on stable states in coral reefs." Theoretical Ecology 15:321-335.

Hock, K., N. H. Wolff, J. C. Ortiz, S. A. Condie, K. R. Anthony, P. G. Blackwell and P. J. Mumby. 2017. Connectivity and systemic resilience of the Great Barrier Reef. PLoS biology 15:e2003355.

Lewis. T.G. 2011. *Network Science: Theory and Applications*. Hoboken: Wiley and Sons.

McManus, L. C., J. R. Watson, V. V. Vasconcelos, and S. A. Levin. 2019. "Stability and recovery of coral-algae systems: the importance of recruitment seasonality and grazing influence." Theoretical Ecology 12:61-72.

Mumby, P. J., A. Hastings, and H. J. Edwards. 2007. "Thresholds and the resilience of Caribbean coral reefs." Nature 450:98.

Treml, E. A., J. Roberts, P. N. Halpin, H. P. Possingham, and C. Riginos. 2015. "The emergent geography of biophysical dispersal barriers across the Indo-West Pacific." Diversity and Distributions 21:465-476.

Wood, S., C. B. Paris, A. Ridgwell, and E. J. Hendy. 2014. "Modelling dispersal and connectivity of broadcast spawning corals at the global scale." Global Ecology and Biogeography 23:1-11.

Wildlife Conservation Society (WCS). 2022. "Launching a Decade of Action for Coral Reefs."
