# Supplementary material for: Dispersal can spread management benefits: Insights from a modeled Fijian coral reef network
Source: Ecol Appl. 2025 Dec 8;35(8):e70156. doi: 10.1002/eap.70156 (PMC12683702; doi:10.1002/eap.70156)
Supplement: Supplementary file 5 — Appendix S5. [file EAP-35-e70156-s002.pdf]

Title: Dispersal can spread management benefits: Insights from a modeled Fijian coral reef network

Journal Name: Ecological Applications

Authors: Ariel Greiner, Marco Andrello, Martin Krkošek, Marie-Josée Fortin, Yashika Nand, Stacy D. Jupiter, Sangeeta Mangubhai, Amelia Wenger, Emily S. Darling

## Appendix S5: More Details on Modeling the Fishery Closure-Based Interventions

First, we calculated a 0.02 degree (~2.22km) radius and a 0.05 degree (~5.55km) radius around the reefs that are currently in fishery closures ('Originally in closure' reefs aka reefs originally in fishery closures, Fig. S1 below). We also considered a 0.01 degree radius but found that only two extra reefs (i.e., only two reefs not in fishery closures) would have been added to fishery closures under that management intervention so we did not include it. To determine which reefs would be within fishery closures if the radii of the fishery closures were expanded within fishing ground boundaries (M1-2km, M3-2km + 10%, M3-2km + 25%) we determined which reefs were within the 0.02 degree radius around each of the 'originally in closure' reefs and were also in the same fishing ground as the original reef in the fishery closure. All the reefs included in the 0.05 degree radius around each of the reefs originally in the fishery closures were included in the 5km fishery closure increase management intervention (i.e., M1-5km). All the reefs that were included in the expanded fishery closures were given the same grazing rate as the original reef in the fishery closure whose radius (or within the radius and fishing ground if under M1-2km or M3-2km + 10% or M3-2km + 25%) they are in.

What if a reef is within multiple expanded fishery closure radii?

- Note: this applies for reefs originally in fishery closures and reefs not initially in fishery closures
- 5km fishery closure management intervention:
    - The reef in question is assigned to the grazing rate of the reef originally in the fishery closure in the same fishing ground UNLESS
        - What if multiple of the reefs originally in the fishery closure are within the same fishing ground as the reef in question?
            - Calculate the average grazing rate of the reefs originally in the fishery closures
        - What if the reef in question is in a different fishing ground from all the reefs originally in the fishery closure whose radii it's contained within?
            - Take the average grazing rate of all the reefs originally in the fishery closure
- 2km fishing ground restricted fishery closure management interventions:
    - The reef in question is assigned to the grazing rate of the reef originally in the fishery closure in the same fishing ground UNLESS
        - What if multiple of the reefs originally in the fishery closure are within the same fishing ground as the reef in question?
            - Calculate the average grazing rate of those reefs originally in the fishery closure
        - What if the reef in question is in a different fishing ground from all of the reefs originally in the fishery closure whose radii it's contained within?
            - It's grazing rate is unchanged (keeps it's initial grazing rate)

Note: For the grazing scenarios under management interventions 1, 2, 5 and 6, we used the multipliers assigned from the baseline simulation run instead of recalculating what the multipliers would be under each of the fishery ground interventions. We did this to ensure consistency across the management interventions.

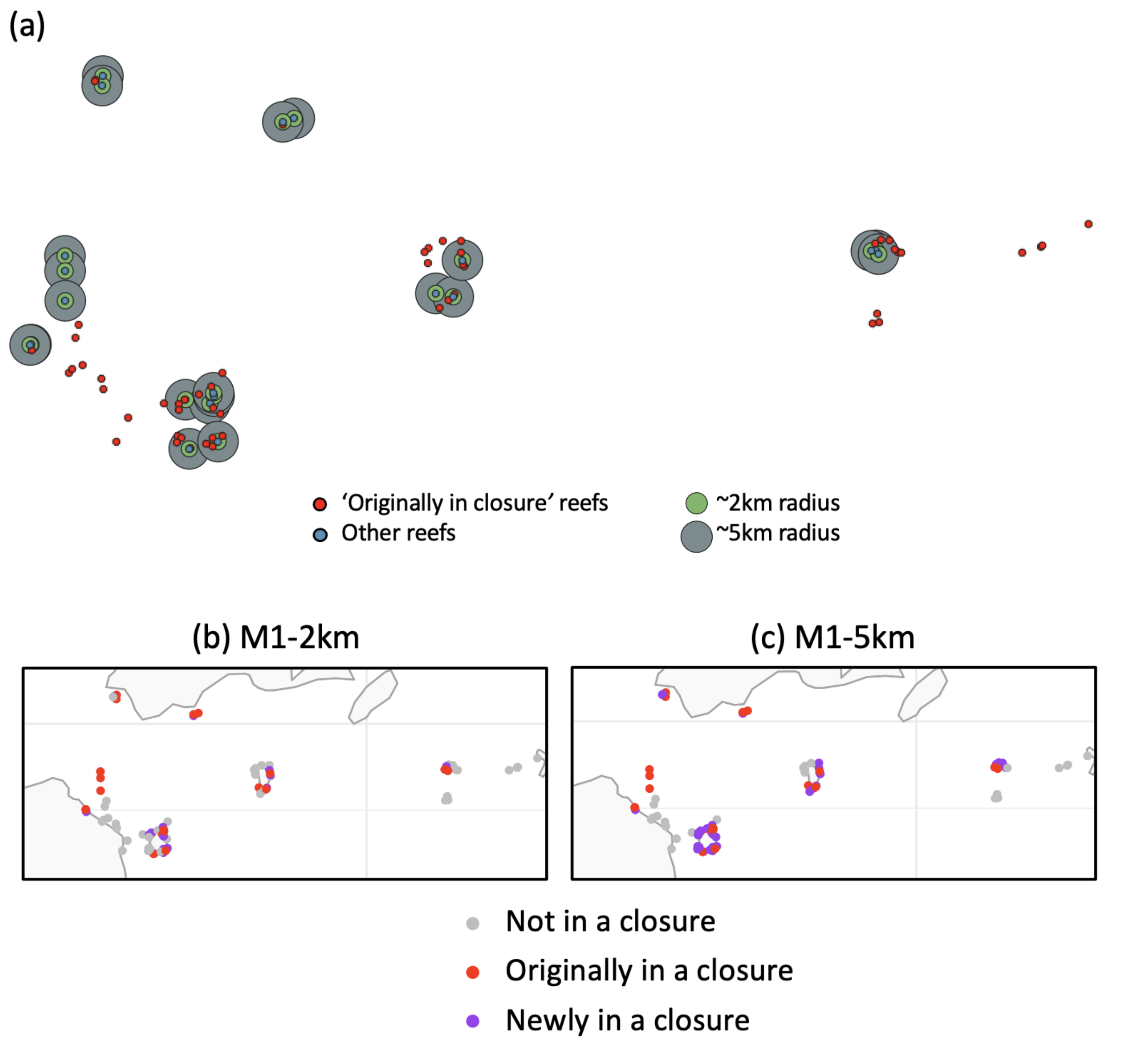


*Figure S1: Determining which Reefs Newly Included in Fishery Closures* - (a) Map showing where all of the reefs originally in the fishery closures are and the number of reefs encompassed by a ~2km radius and ~5km radius around each of them. (b) Map showing the reefs newly included in fishery closures under M1-2km and both M3's (i.e., when extend fishery closure radius to ~2km but restrict it to only include reefs in the same fishing ground as the reef originally in the fishery closure). (c) Map showing the reefs newly included in fishery closures under M1-5km (i.e., when extend fishery closure radius to ~5km). In (a), (b) and (c) the 'Originally in closure' reefs are all the same.
