# Supplementary material for: Dispersal can spread management benefits: Insights from a modeled Fijian coral reef network
Source: Ecol Appl. 2025 Dec 8;35(8):e70156. doi: 10.1002/eap.70156 (PMC12683702; doi:10.1002/eap.70156)
Supplement: Supplementary file 6 — Appendix S6. [file EAP-35-e70156-s001.pdf]

Title: Dispersal can spread management benefits: Insights from a modeled Fijian coral reef network

Journal Name: Ecological Applications

Authors: Ariel Greiner, Marco Andrello, Martin Krkošek, Marie-Josée Fortin, Yashika Nand, Stacy D. Jupiter, Sangeeta Mangubhai, Amelia Wenger, Emily S. Darling

Appendix S6: Plots of Management Intervention Improvements Showing Fishery Closure Designation of Each Reef

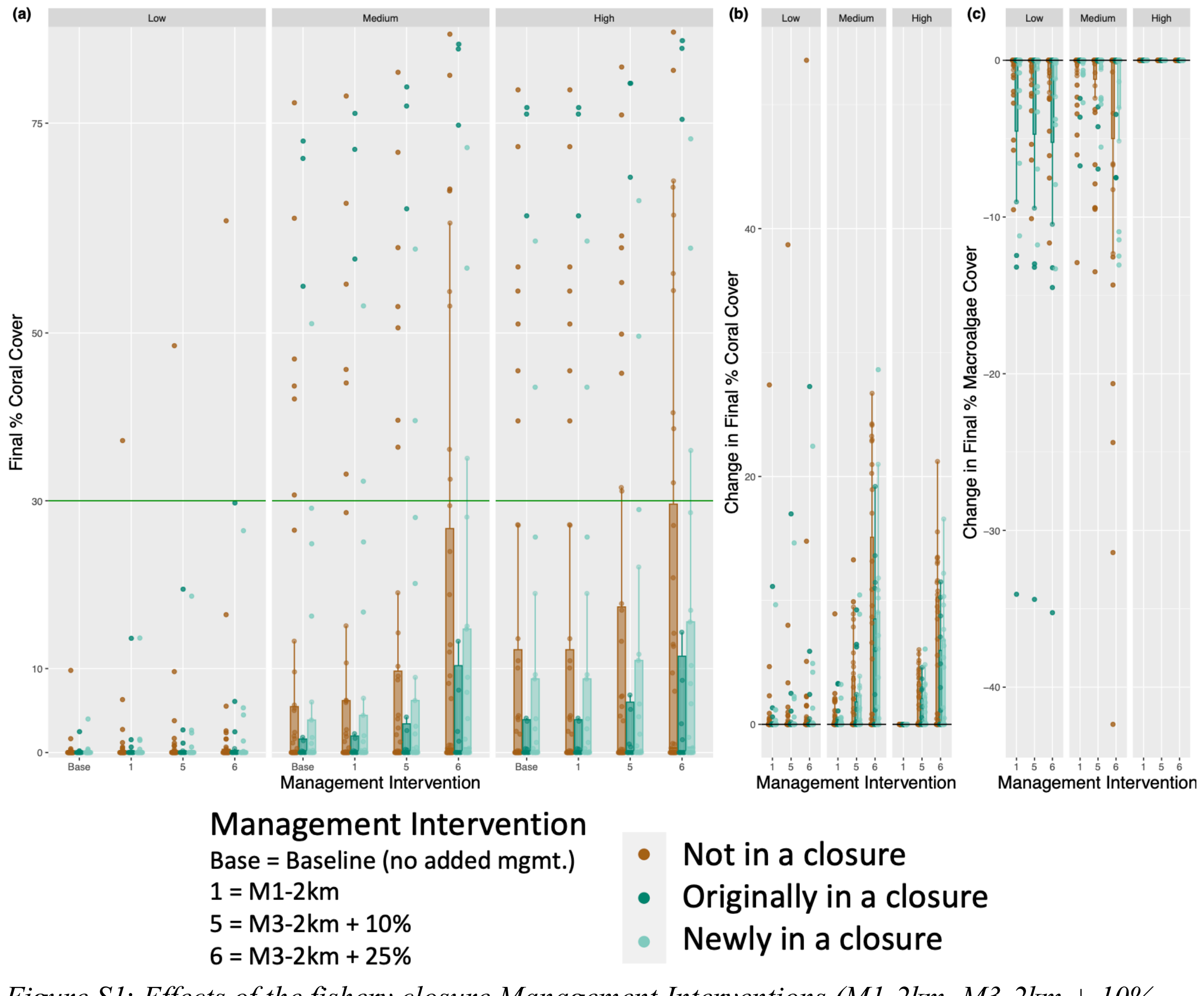

Figure S1: *Effects of the fishery closure Management Interventions (M1-2km, M3-2km + 10%, M3-2km + 25%) bound by fishing ground restrictions* - a-c show the effect of the management intervention on the final coral cover of each reef, while each panel shows the effect of the management under each grazing scenario. The reefs are separated by management status under the M1,M3 management interventions, with the teal dots representing reefs that are only under fishery closure protection when the fishery closure is extended and the light blue dots representing reefs that were originally and remain under fishery closure protection under all the interventions and the baseline simulation. (a) Final percent coral cover in each reef, with a green line at 30% indicating a healthy reef (Birrell et al., 2020; WCS 2022). (b) Difference in the percent coral cover in each reef between each management intervention and the baseline simulation, the black line at 0 indicates the reefs that went through no change in percent coral cover. (c) Difference in the percent macroalgal cover in each reef between each management intervention and the baseline simulation, the black line at 0 indicates the reefs that went through no change in percent macroalgal cover. ‘baseline’ represents the baseline simulations with no modeled management interventions. In (a-c), each point represents the final % coral cover of a particular reef and box plots showing the inter-quartile range of the values are placed behind the

points to indicate spread; in (a) the points are jittered along the *x*-axis to make it easier to distinguish individual points.

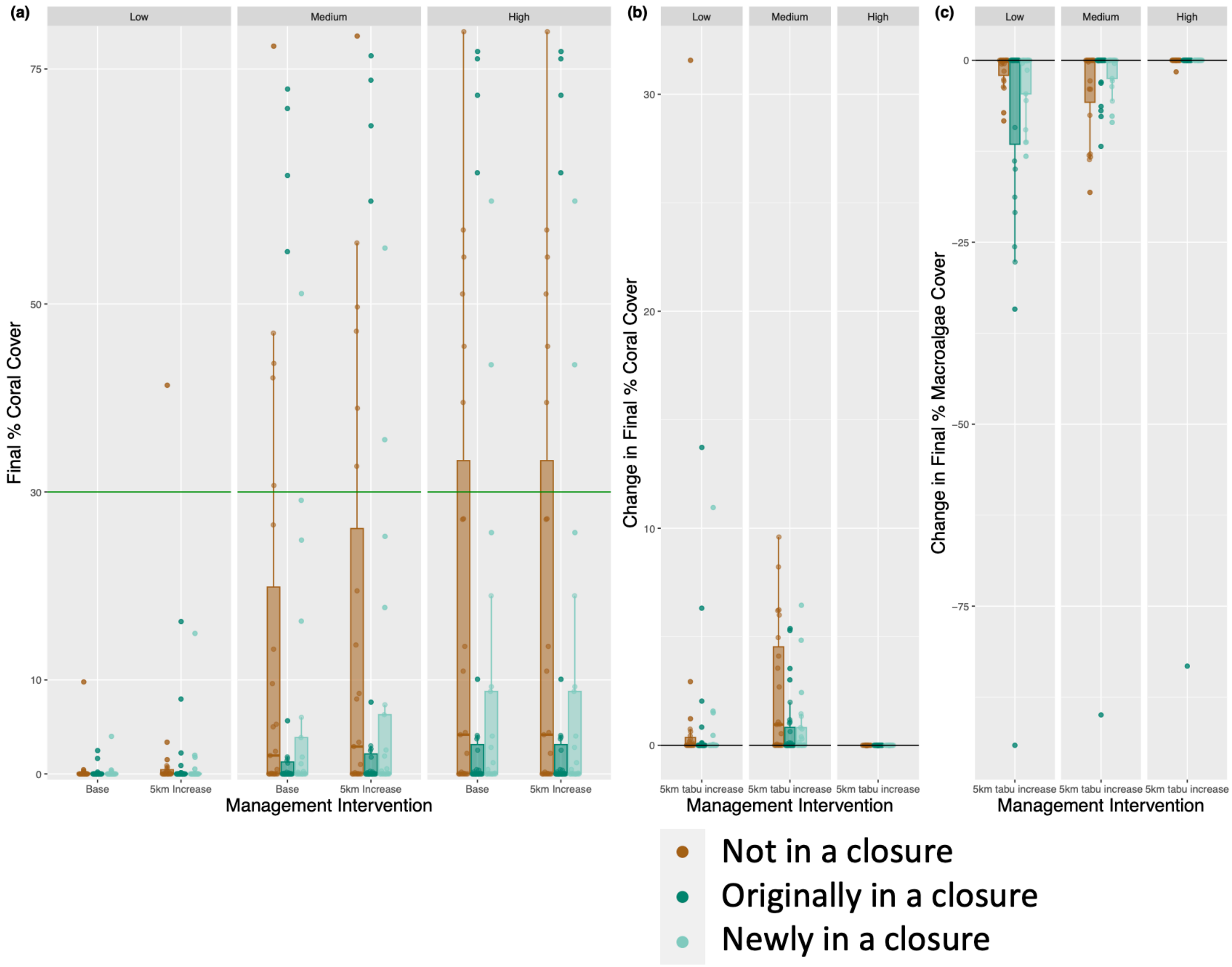

Figure S2: *Effects of M1-5km* - a-c show the effect of the management intervention on the final coral cover of each reef, while each panel shows the effect of the management under each grazing scenario. The reefs are separated by management status under the 5km fishery closure increase management intervention (i.e., M1-5km), with the teal dots representing reefs that are only under fishery closure protection when the fishery closure is extended to ~5km (i.e., M1-5km) and the light blue dots representing reefs that were originally and remain under fishery closure protection under all the interventions and the baseline simulation. (a) Final percent coral cover in each reef, with a green line at 30% indicating a healthy reef (Birrell et al., 2020; WCS 2022). (b) Difference in the percent coral cover in each reef between each management intervention and the baseline simulation, the black line at 0 indicates the reefs that went through no change in percent coral cover. (c) Difference in the percent macroalgal cover in each reef between each management intervention and the baseline simulation, the black line at 0 indicates the reefs that went through no change in percent macroalgal cover. 'baseline' represents the

baseline simulations with no modeled management interventions. In (a-c), each point represents the final % coral cover of a particular reef and box plots showing the inter-quartile range of the values are placed behind the points to indicate spread; in (a) the points are jittered along the *x*-axis to make it easier to distinguish individual points.

References

Birrell, C. L., E. Sola, R. H. Bennett, D. van Beuningen, H. M. Costa, J. J. Sitoe, N. Sidat, S. Fernando, E.S. Darling, N.A. Muthiga and T. R. McClanahan. 2020. "A summary of WCS knowledge of the state of coral reefs in Mozambique." Wildlife Conservation Society, Maputo, Mozambique. https://biblioteca.biofund.org.mz/wp-content/uploads/2021/03/1616752045-2020_WCS_Coral_Reefs_in_Mozambique.pdf

Wildlife Conservation Society (WCS). 2022. "Launching a Decade of Action for Coral Reefs." https://cdn.wcs.org/2021/04/21/99xudme990_4.16.21_English_CBD_Rec_2_Pager.pdf?_gl=1*1f71558*_ga*MTk4MTYyMzY4Ni4xNjc0ODM0MjI1*_ga_BTX9HXMYSX*MTY4MDE5Nzk2MC4xNi4wLjE2ODAxOTc5NjAuNjAuMC4w
