# Supplementary material for: Dispersal can spread management benefits: Insights from a modeled Fijian coral reef network
Source: Ecol Appl. 2025 Dec 8;35(8):e70156. doi: 10.1002/eap.70156 (PMC12683702; doi:10.1002/eap.70156)
Supplement: Supplementary file 7 — Appendix S7. [file EAP-35-e70156-s006.pdf]

Title: Dispersal can spread management benefits: Insights from a modeled Fijian coral reef network

Journal Name: Ecological Applications

Authors: Ariel Greiner, Marco Andrello, Martin Krkošek, Marie-Josée Fortin, Yashika Nand, Stacy D. Jupiter, Sangeeta Mangubhai, Amelia Wenger, Emily S. Darling

Appendix S7: Higher Sedimentation improvement Interventions

Results from the {30, 40, 50, 60, 70, 80, and 90} % interventions included below alongside the results from the other management interventions.

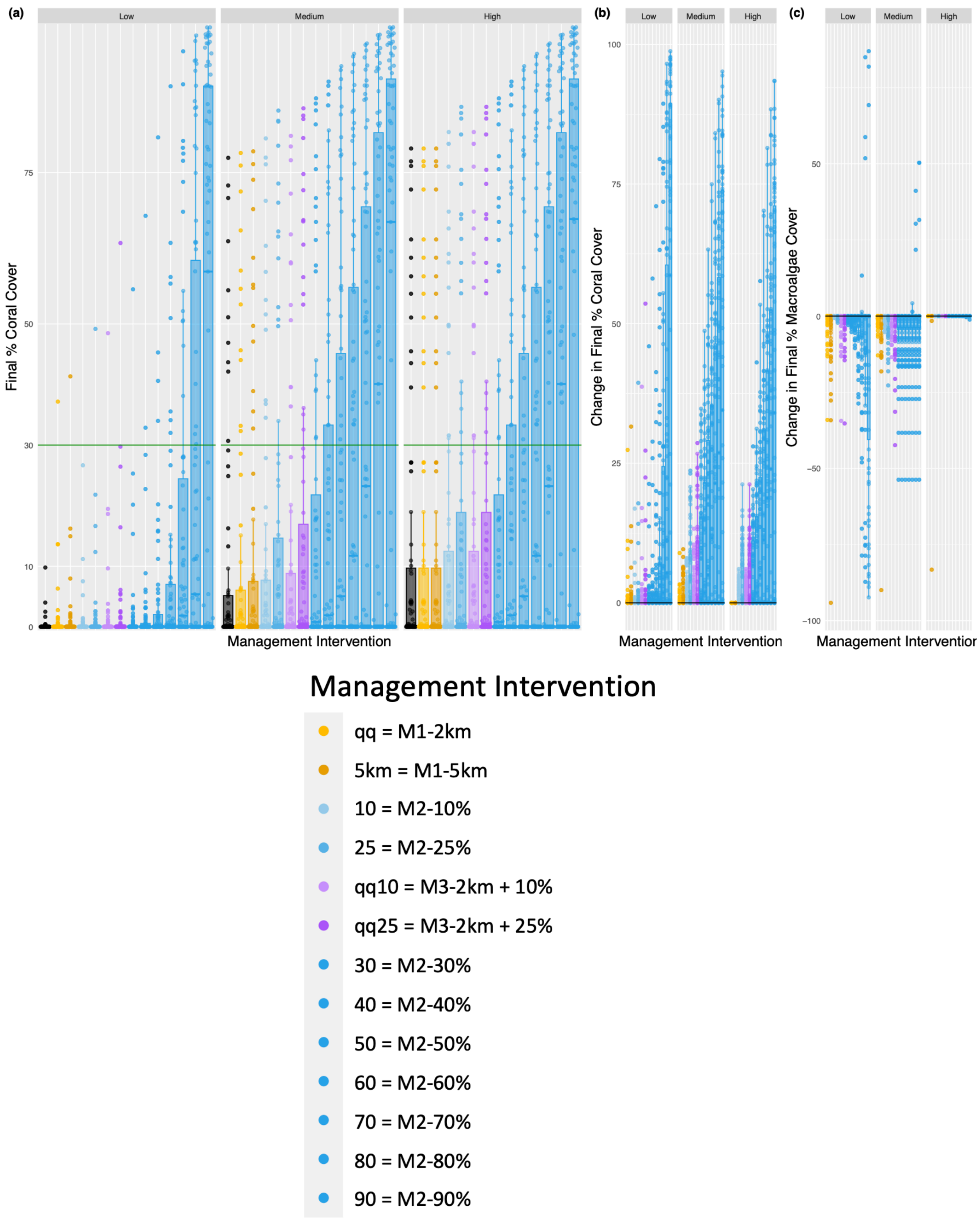

Figure S1: *Effects of all the Management Interventions* - a-c show the effect of the management intervention on the final coral cover of each reef, while each panel shows the effect of the management under each grazing scenario. (a) Final percent coral cover in each reef, with a green

line at 30% indicating a healthy reef (Birrell et al., 2020; WCS 2022). The black dots ('base') represents the baseline simulation with no additional interventions. (b) Difference in the percent coral cover in each reef between each management intervention and the baseline simulation, the black line at 0 indicates the reefs that went through no change in percent coral cover. (c) Difference in the percent macroalgal cover in each reef between each management intervention and the baseline simulation, the black line at 0 indicates the reefs that went through no change in percent macroalgal cover. In (a-c), each point represents the final % coral cover of a particular reef and box plots showing the inter-quartile range of the values are placed behind the points to indicate spread; in (a) the points are jittered along the x-axis to make it easier to distinguish individual points.

Table S1: Number of reefs with less than 1% final coral cover

Grazing Scenario/Management Interventions	Low Scenario	Medium Scenario	High Scenario
2km closure Increase within fishing grounds	66	46	44
5km closure Increase	66	45	44
10% Water Quality Improvement	69	45	43
25% Water Quality Improvement	64	41	40
Low Mixed	64	45	43
High Mixed	61	41	40
30% Water Quality Improvement	63	39	39
40% Water Quality Improvement	59	35	35
50% Water Quality Improvement	49	35	35
60% Water Quality Improvement	45	34	34
70% Water Quality Improvement	35	32	32

80% Water Quality Improvement	33	31	31
90% Water Quality Improvement	30	30	30

References

Birrell, C. L., E. Sola, R. H. Bennett, D. van Beuningen, H. M. Costa, J. J. Sitoe, N. Sidat, S. Fernando, E.S. Darling, N.A. Muthiga and T. R. McClanahan. 2020. "A summary of WCS knowledge of the state of coral reefs in Mozambique." Wildlife Conservation Society, Maputo, Mozambique. https://biblioteca.biofund.org.mz/wp-content/uploads/2021/03/1616752045-2020_WCS_Coral_Reefs_in_Mozambique.pdf

Wildlife Conservation Society (WCS). 2022. "Launching a Decade of Action for Coral Reefs." https://cdn.wcs.org/2021/04/21/99xudme990_4.16.21_English_CBD_Rec_2_Pager.pdf?_gl=1*1f71558*_ga*MTk4MTYyMzY4Ni4xNjc0ODM0MjI1*_ga_BTX9HXMYSX*MTY4MDE5Nzk2MC4xNi4wLjE2ODAxOTc5NjAuNjAuMC4w
