# Supplementary material for: Dispersal can spread management benefits: Insights from a modeled Fijian coral reef network
Source: Ecol Appl. 2025 Dec 8;35(8):e70156. doi: 10.1002/eap.70156 (PMC12683702; doi:10.1002/eap.70156)
Supplement: Supplementary file 8 — Appendix S8. [file EAP-35-e70156-s003.pdf]

Title: Dispersal can spread management benefits: Insights from a modeled Fijian coral reef network

Journal Name: Ecological Applications

Authors: Ariel Greiner, Marco Andrello, Martin Krkošek, Marie-Josée Fortin, Yashika Nand, Stacy D. Jupiter, Sangeeta Mangubhai, Amelia Wenger, Emily S. Darling

Appendix S8: Results of the Initial Condition Sensitivity Analyses

In order to directly assess whether the multiple stable state dynamics present in the model (see Appendix S3) determine the final coral cover of the reefs in the 75-Reef Fiji Model and to assess the sensitivity of the management results to the empirically derived initial conditions, we ran the 75-Reef Fiji Model under various initial conditions (see Table S1 below). For Overall Scenarios 1-6 and 13-18, every reef in the 75-Reef Fiji Model was given the same initial coral cover value and initial macroalgal cover value (the ones listed in Table S1; initial value of F_i for all reefs was set in the same way as before, see Appendix S3). For Overall Scenarios 7-12, the initial cover values of the reefs were set by moving the empirical median value to the median value listed in Table S1 and then re-adjusting all of the values to be between 0 and 1 as necessary (see https://doi.org/10.5281/zenodo.17340984 for more details; initial value of F_i for all reefs was set in the same way as before, see Appendix S3). Because of this constraint and because adding this variation between reefs did not result in different final benthic cover than the corresponding non-varying overall scenarios, we did not run scenarios where the median initial coral cover value was 0.01 and 0.98. We chose initial conditions at the extremes (or centred around the extremes) of the state space (as the state variables in this model are bound between 0 and 1 as they are proportions of total space) as this is where we are most likely to observe sensitivity to initial conditions in final benthic cover values owing to local multiple stable state dynamics. If the multiple stable state dynamics present in the 75-Reef Fiji Network Model behave similarly to

those in the single-reef and two-reef versions of the model (Mumby et al., 2007; Elmhirst et al., 2009; Greiner et al., 2022), we would anticipate observing negligible impacts of varying initial conditions under the low and high grazing scenarios and large impacts of varying initial conditions at the medium grazing scenario.

Varying the initial conditions did not noticeably alter the relative effectiveness of the different management interventions or change the number of reefs with final coral cover >30% under the low and high grazing scenario (Table S2, Fig. S1-6a). Under the medium grazing scenario it did not noticeably alter the effectiveness of the different management interventions beyond increasing the effectiveness of the M3-2km+25% intervention (i.e. larger changes in final benthic cover, one additional reef had a final coral cover >30% compared to the empirical initial condition values) (Table S2, Fig. S1-6).

Table S1: *Overall Scenarios* - This table describes all of the overall scenarios (Grazing*(Initial coral cover, initial macroalgae cover) shown in Table S2 below.

Overall Scenario	Grazing Scenario	Initial Coral Cover	Initial Macroalgae Cover
1	Low	0.01	0.98
2	Medium	0.01	0.98
3	High	0.01	0.98
4	Low	0.1	0.8
5	Medium	0.1	0.8
6	High	0.1	0.8
7	Low	Median = 0.1	Median = 0.8
8	Medium	Median = 0.1	Median = 0.8
9	High	Median = 0.1	Median = 0.8
10	Low	Median = 0.8	Median = 0.1
11	Medium	Median = 0.8	Median = 0.1
12	High	Median = 0.8	Median = 0.1
13	Low	0.8	0.1
14	Medium	0.8	0.1
15	High	0.8	0.1
16	Low	0.98	0.01
17	Medium	0.98	0.01
18	High	0.98	0.01

Table S2: *Number of Reefs >30% Final Coral Cover* - The numbers in the table below represent the number of reefs under each management intervention and overall scenario (grazing scenario and initial condition scenario) whose final percent coral cover is >30%.

Overall Scenario/ Management Intervention	1	2	3	4	5	6	7	8	9	10	11	12	13	14	15	16	17	18
1. M1-2km	1	11	12	1	11	12	1	11	12	1	11	12	1	11	12	1	11	12

2. M1-5km	1	12	12	1	12	12	1	12	12	1	12	12	1	12	12	1	12	12
3. M2-10%	0	12	14	0	12	14	0	12	14	0	12	14	0	12	14	0	12	14
4. M2-25%	1	13	16	1	13	16	1	13	16	1	13	16	1	13	16	1	13	16
5. M3-2km+10%	1	12	14	1	12	14	1	12	14	1	12	14	1	12	14	1	12	14
6. M3-2km+25%	1	15	16	1	15	16	1	15	16	1	**16**	16	1	**16**	16	1	**16**	16

Note: Numbers in **red** indicate instances where the number of reefs with final percent coral cover >30% under that management intervention and grazing scenario is different from the empirical initial condition value under the same management intervention and grazing scenario (Main Text - Table 2).

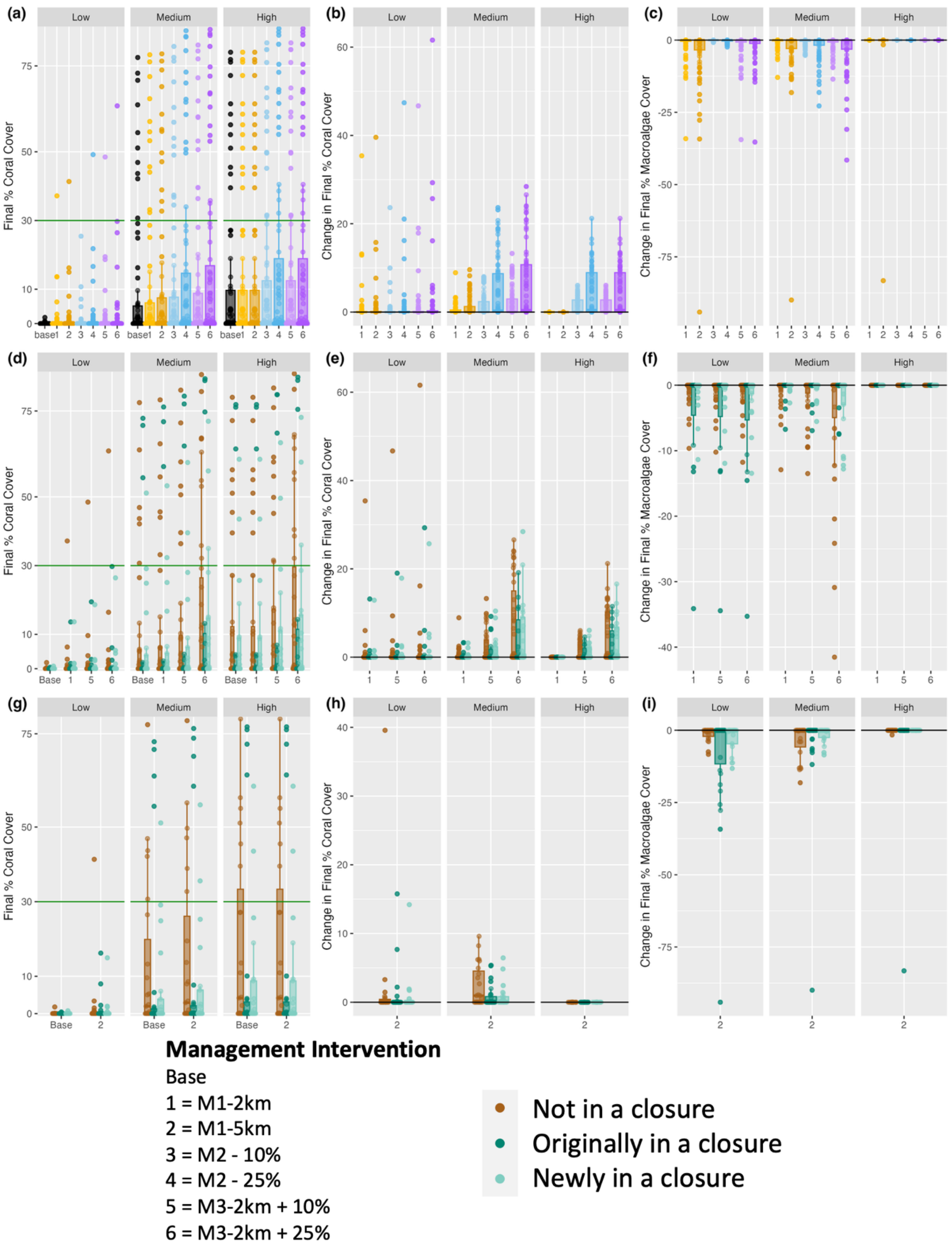

Figure S1. *Effects of the Management Interventions When* C_{init} *= 0.01,* M_{init} *= 0.98* - Each panel shows the effect of the management intervention on the final coral cover of each reef, while each

panel shows the effect of the management under each grazing scenario. (d, e, f) Effects of fishery closure management interventions bound by fishing ground restrictions; the reefs are separated by management status under the M1, M3 management interventions, with the teal dots representing reefs that are only under fishery closure protection when the fishery closure is extended and the light blue dots representing reefs that were originally and remain under fishery closure protection under all the interventions and the baseline simulation. (g, h, i) Effects of fishery closure management intervention not bound by fishing ground restrictions; the reefs are separated by management status under the 5km fishery closure increase management intervention (i.e., M1-5km), with the teal dots representing reefs that are only under fishery closure protection when the fishery closure is extended to ~5km (i.e., M1- 5km) and the light blue dots representing reefs that were originally and remain under fishery closure protection under all the interventions and the baseline simulation. (a, d, g) Final percent coral cover in each reef, with a green line at 30% indicating a healthy reef (Birrell et al., 2020; WCS 2022). (b, e, h) Difference in the percent coral cover in each reef between each management intervention and the baseline simulation; the black line at 0 indicates the reefs that went through no change in percent coral cover. (c, f, i) Difference in the percent macroalgal cover in each reef between each management intervention and the baseline simulation; the black line at 0 indicates the reefs that went through no change in percent macroalgal cover. 'Base' represents the baseline simulations with no modeled management intervention. Each point represents the final % coral cover of a particular reef and box plots showing the inter-quartile range of the values are placed behind the points to indicate spread; in (a, d, g) the points are jittered along the *x*-axis to make it easier to distinguish individual points.

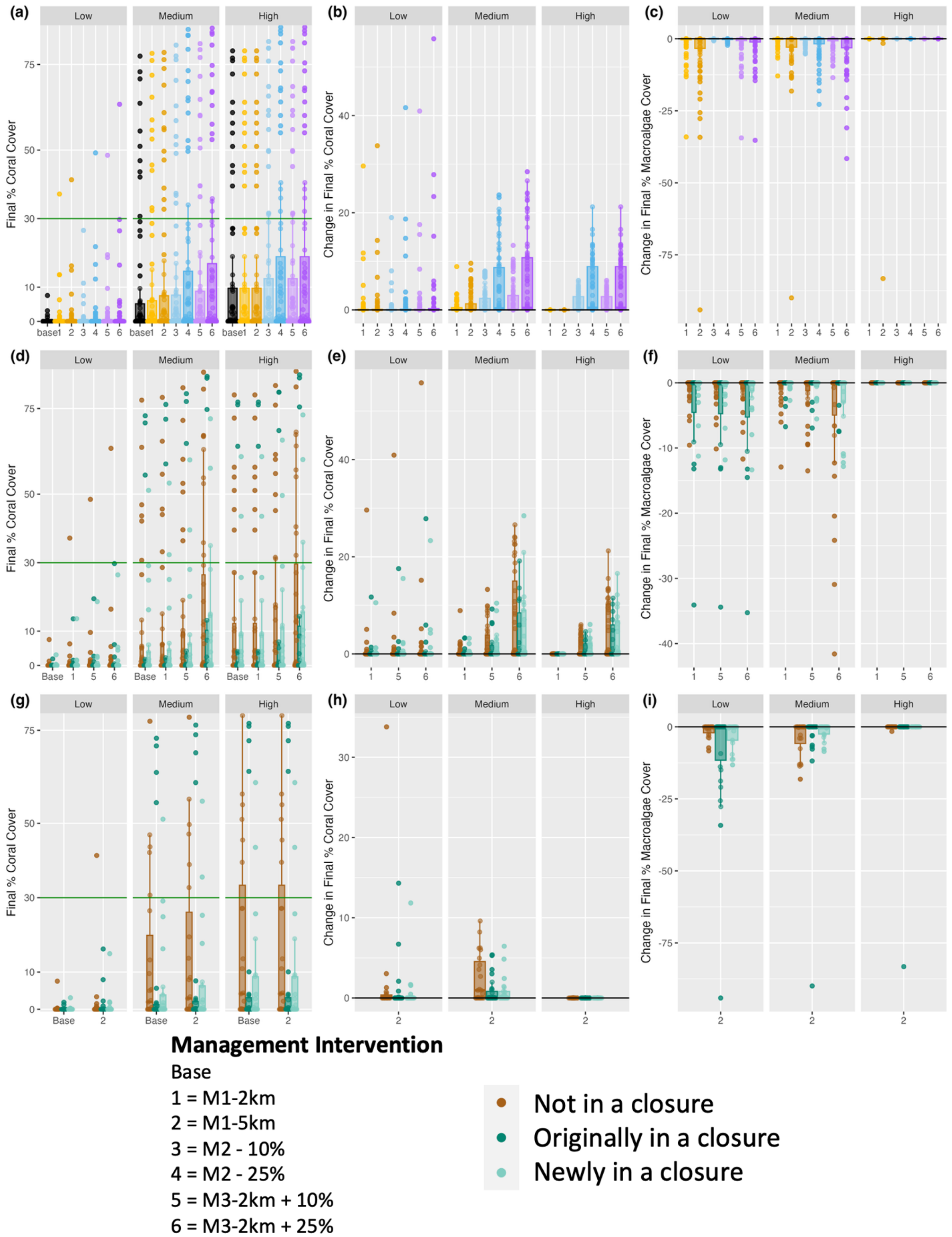

Figure S2. *Effects of the Management Interventions When $C_{init} = 0.1$, $M_{init} = 0.8$* - Each panel shows the effect of the management intervention on the final coral cover of each reef, while each

panel shows the effect of the management under each grazing scenario. (d, e, f) Effects of fishery closure management interventions bound by fishing ground restrictions; the reefs are separated by management status under the M1, M3 management interventions, with the teal dots representing reefs that are only under fishery closure protection when the fishery closure is extended and the light blue dots representing reefs that were originally and remain under fishery closure protection under all the interventions and the baseline simulation. (g, h, i) Effects of fishery closure management intervention not bound by fishing ground restrictions; the reefs are separated by management status under the 5km fishery closure increase management intervention (i.e., M1-5km), with the teal dots representing reefs that are only under fishery closure protection when the fishery closure is extended to ~5km (i.e., M1- 5km) and the light blue dots representing reefs that were originally and remain under fishery closure protection under all the interventions and the baseline simulation. (a, d, g) Final percent coral cover in each reef, with a green line at 30% indicating a healthy reef (Birrell et al., 2020; WCS 2022). (b, e, h) Difference in the percent coral cover in each reef between each management intervention and the baseline simulation; the black line at 0 indicates the reefs that went through no change in percent coral cover. (c, f, i) Difference in the percent macroalgal cover in each reef between each management intervention and the baseline simulation; the black line at 0 indicates the reefs that went through no change in percent macroalgal cover. ‘Base’ represents the baseline simulations with no modeled management intervention. Each point represents the final % coral cover of a particular reef and box plots showing the inter-quartile range of the values are placed behind the points to indicate spread; in (a, d, g) the points are jittered along the *x*-axis to make it easier to distinguish individual points.

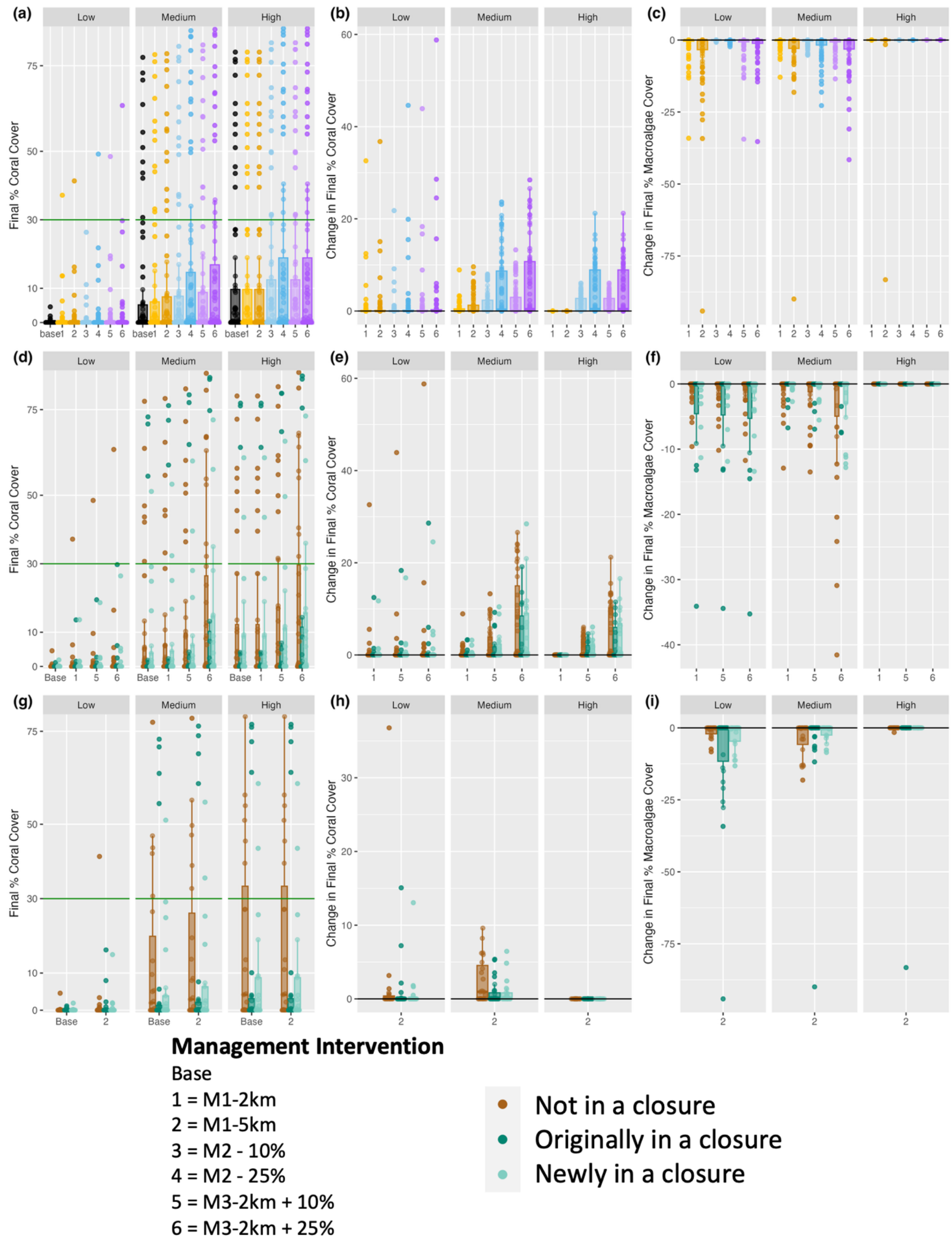

Figure S3. *Effects of the Management Interventions When median(C_{init}) = 0.1, median(M_{init}) = 0.8* - Each panel shows the effect of the management intervention on the final coral cover of each

reef, while each panel shows the effect of the management under each grazing scenario. (d, e, f) Effects of fishery closure management interventions bound by fishing ground restrictions; the reefs are separated by management status under the M1, M3 management interventions, with the teal dots representing reefs that are only under fishery closure protection when the fishery closure is extended and the light blue dots representing reefs that were originally and remain under fishery closure protection under all the interventions and the baseline simulation. (g, h, i) Effects of fishery closure management intervention not bound by fishing ground restrictions; the reefs are separated by management status under the 5km fishery closure increase management intervention (i.e., M1-5km), with the teal dots representing reefs that are only under fishery closure protection when the fishery closure is extended to ~5km (i.e., M1- 5km) and the light blue dots representing reefs that were originally and remain under fishery closure protection under all the interventions and the baseline simulation. (a, d, g) Final percent coral cover in each reef, with a green line at 30% indicating a healthy reef (Birrell et al., 2020; WCS 2022). (b, e, h) Difference in the percent coral cover in each reef between each management intervention and the baseline simulation; the black line at 0 indicates the reefs that went through no change in percent coral cover. (c, f, i) Difference in the percent macroalgal cover in each reef between each management intervention and the baseline simulation; the black line at 0 indicates the reefs that went through no change in percent macroalgal cover. 'Base' represents the baseline simulations with no modeled management intervention. Each point represents the final % coral cover of a particular reef and box plots showing the inter-quartile range of the values are placed behind the points to indicate spread; in (a, d, g) the points are jittered along the *x*-axis to make it easier to distinguish individual points.

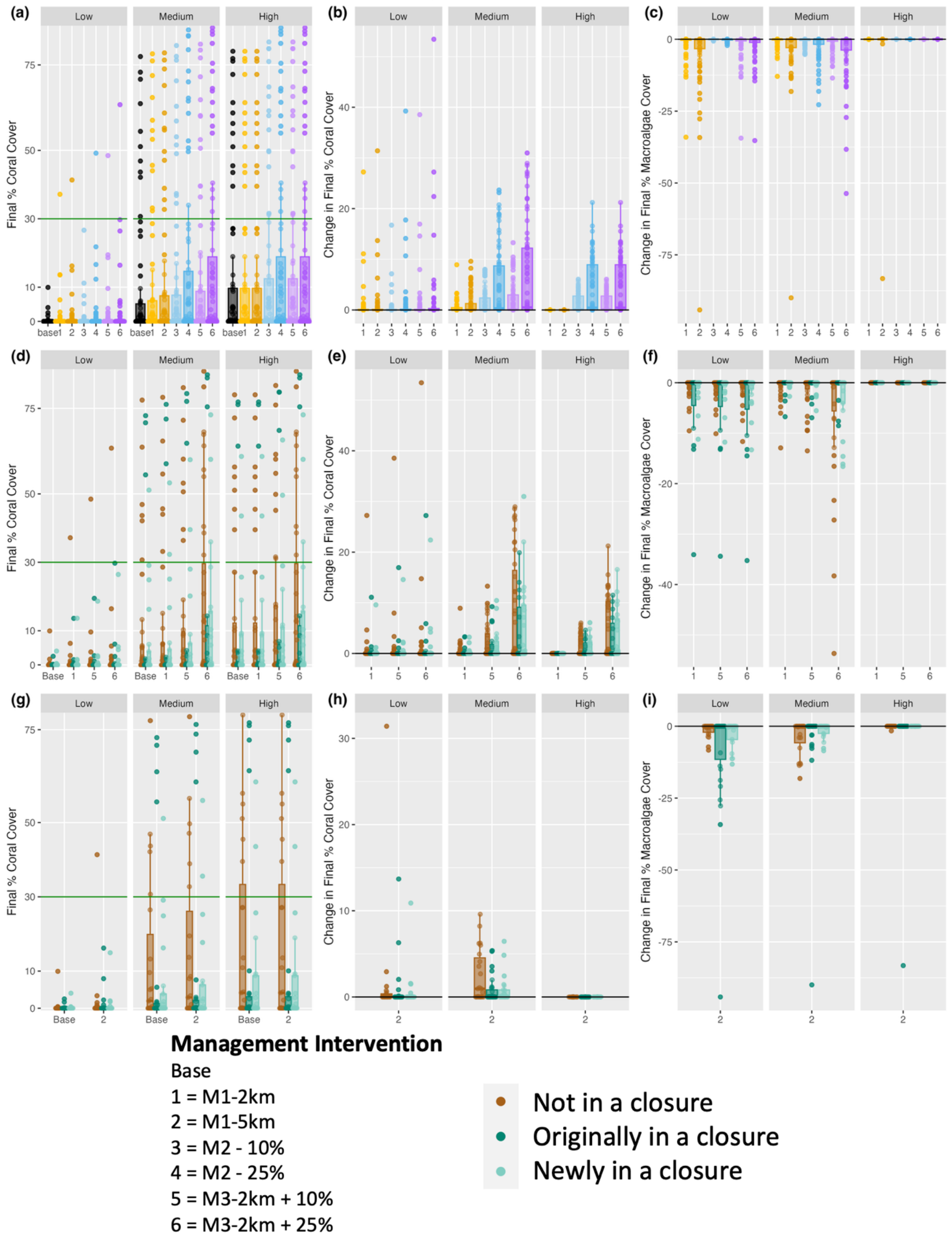

Figure S4. *Effects of the Management Interventions When median(C_{init}) = 0.8, median(M_{init}) = 0.1* - Each panel shows the effect of the management intervention on the final coral cover of each

reef, while each panel shows the effect of the management under each grazing scenario. (d, e, f) Effects of fishery closure management interventions bound by fishing ground restrictions; the reefs are separated by management status under the M1, M3 management interventions, with the teal dots representing reefs that are only under fishery closure protection when the fishery closure is extended and the light blue dots representing reefs that were originally and remain under fishery closure protection under all the interventions and the baseline simulation. (g, h, i) Effects of fishery closure management intervention not bound by fishing ground restrictions; the reefs are separated by management status under the 5km fishery closure increase management intervention (i.e., M1-5km), with the teal dots representing reefs that are only under fishery closure protection when the fishery closure is extended to ~5km (i.e., M1- 5km) and the light blue dots representing reefs that were originally and remain under fishery closure protection under all the interventions and the baseline simulation. (a, d, g) Final percent coral cover in each reef, with a green line at 30% indicating a healthy reef (Birrell et al., 2020; WCS 2022). (b, e, h) Difference in the percent coral cover in each reef between each management intervention and the baseline simulation; the black line at 0 indicates the reefs that went through no change in percent coral cover. (c, f, i) Difference in the percent macroalgal cover in each reef between each management intervention and the baseline simulation; the black line at 0 indicates the reefs that went through no change in percent macroalgal cover. 'Base' represents the baseline simulations with no modeled management intervention. Each point represents the final % coral cover of a particular reef and box plots showing the inter-quartile range of the values are placed behind the points to indicate spread; in (a, d, g) the points are jittered along the *x*-axis to make it easier to distinguish individual points.

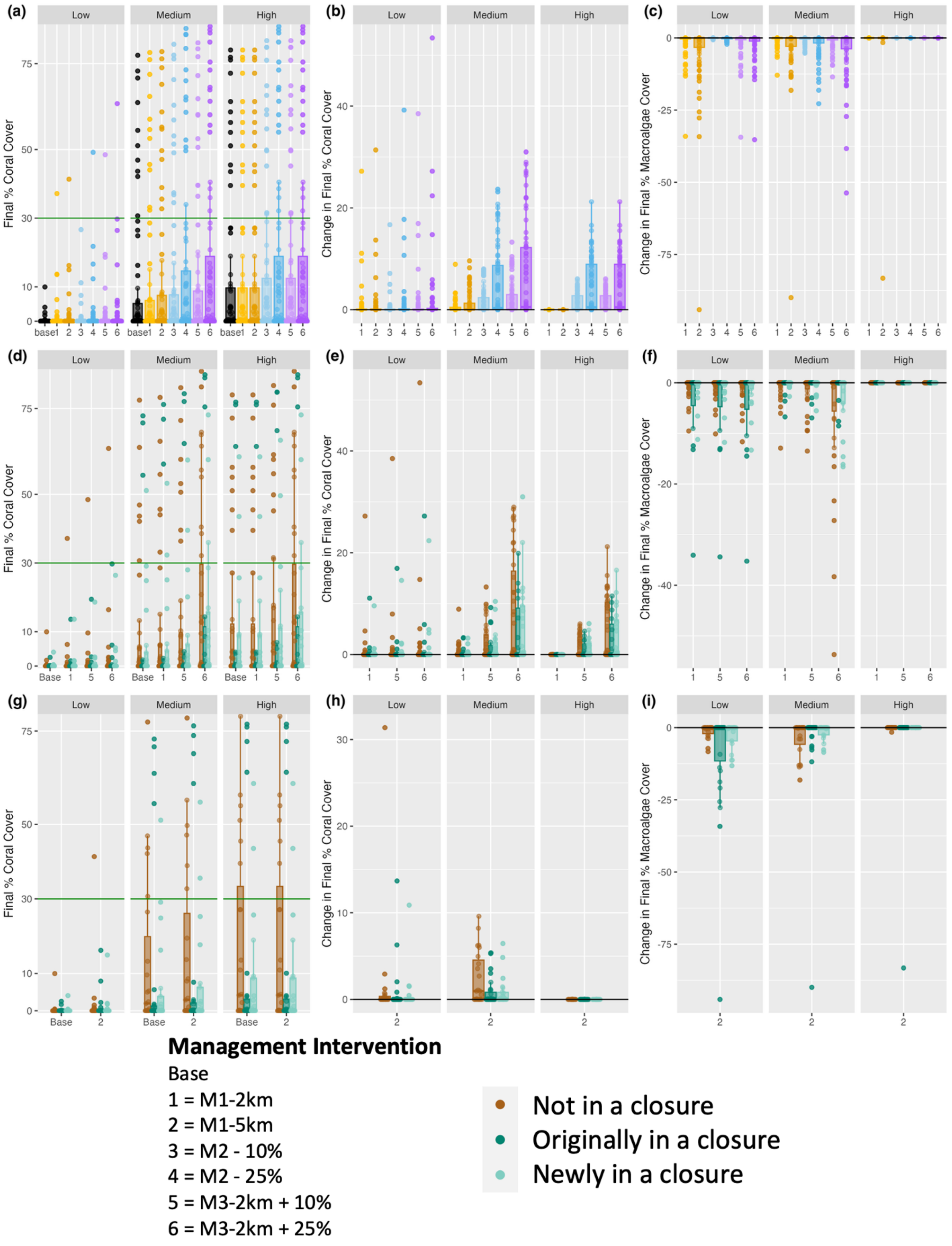

Figure S5. *Effects of the Management Interventions When C_{init} = 0.8, M_{init} = 0.1* - Each panel shows the effect of the management intervention on the final coral cover of each reef, while each

panel shows the effect of the management under each grazing scenario. (d, e, f) Effects of fishery closure management interventions bound by fishing ground restrictions; the reefs are separated by management status under the M1, M3 management interventions, with the teal dots representing reefs that are only under fishery closure protection when the fishery closure is extended and the light blue dots representing reefs that were originally and remain under fishery closure protection under all the interventions and the baseline simulation. (g, h, i) Effects of fishery closure management intervention not bound by fishing ground restrictions; the reefs are separated by management status under the 5km fishery closure increase management intervention (i.e., M1-5km), with the teal dots representing reefs that are only under fishery closure protection when the fishery closure is extended to ~5km (i.e., M1- 5km) and the light blue dots representing reefs that were originally and remain under fishery closure protection under all the interventions and the baseline simulation. (a, d, g) Final percent coral cover in each reef, with a green line at 30% indicating a healthy reef (Birrell et al., 2020; WCS 2022). (b, e, h) Difference in the percent coral cover in each reef between each management intervention and the baseline simulation; the black line at 0 indicates the reefs that went through no change in percent coral cover. (c, f, i) Difference in the percent macroalgal cover in each reef between each management intervention and the baseline simulation; the black line at 0 indicates the reefs that went through no change in percent macroalgal cover. 'Base' represents the baseline simulations with no modeled management intervention. Each point represents the final % coral cover of a particular reef and box plots showing the inter-quartile range of the values are placed behind the points to indicate spread; in (a, d, g) the points are jittered along the *x*-axis to make it easier to distinguish individual points.

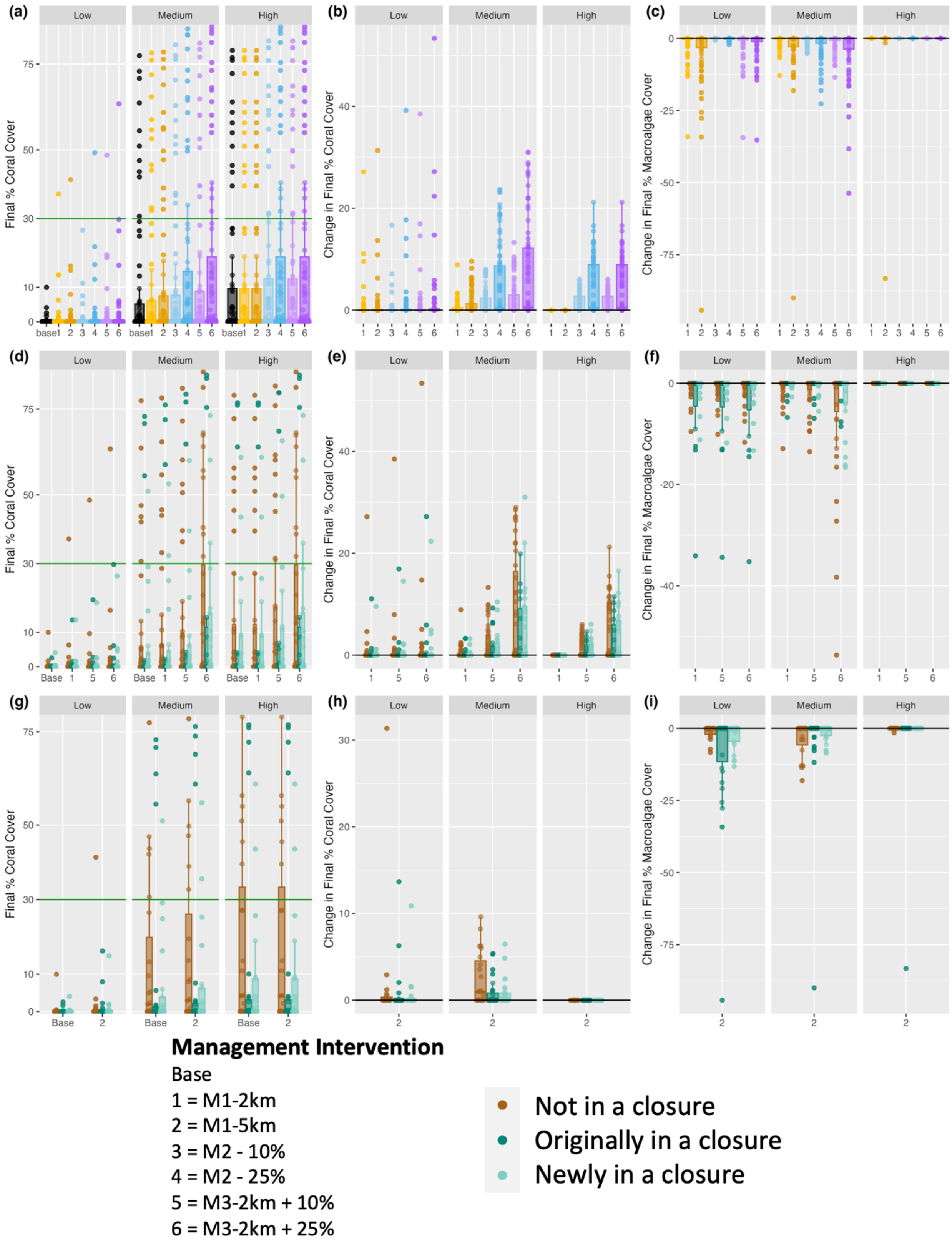

Figure S6. *Effects of the Management Interventions When C_{init} = 0.98, M_{init} = 0.01* - Each panel shows the effect of the management intervention on the final coral cover of each reef, while each

panel shows the effect of the management under each grazing scenario. (d, e, f) Effects of fishery closure management interventions bound by fishing ground restrictions; the reefs are separated by management status under the M1, M3 management interventions, with the teal dots representing reefs that are only under fishery closure protection when the fishery closure is extended and the light blue dots representing reefs that were originally and remain under fishery closure protection under all the interventions and the baseline simulation. (g, h, i) Effects of fishery closure management intervention not bound by fishing ground restrictions; the reefs are separated by management status under the 5km fishery closure increase management intervention (i.e., M1-5km), with the teal dots representing reefs that are only under fishery closure protection when the fishery closure is extended to ~5km (i.e., M1- 5km) and the light blue dots representing reefs that were originally and remain under fishery closure protection under all the interventions and the baseline simulation. (a, d, g) Final percent coral cover in each reef, with a green line at 30% indicating a healthy reef (Birrell et al., 2020; WCS 2022). (b, e, h) Difference in the percent coral cover in each reef between each management intervention and the baseline simulation; the black line at 0 indicates the reefs that went through no change in percent coral cover. (c, f, i) Difference in the percent macroalgal cover in each reef between each management intervention and the baseline simulation; the black line at 0 indicates the reefs that went through no change in percent macroalgal cover. 'Base' represents the baseline simulations with no modeled management intervention. Each point represents the final % coral cover of a particular reef and box plots showing the inter-quartile range of the values are placed behind the points to indicate spread; in (a, d, g) the points are jittered along the *x*-axis to make it easier to distinguish individual points.

References

Birrell, C. L., E. Sola, R. H. Bennett, D. van Beuningen, H. M. Costa, J. J. Sitoe, N. Sidat, S. Fernando, E.S. Darling, N.A. Muthiga and T. R. McClanahan. 2020. "A summary of WCS knowledge of the state of coral reefs in Mozambique." Wildlife Conservation Society, Maputo, Mozambique. https://biblioteca.biofund.org.mz/wp-content/uploads/2021/03/1616752045-2020_WCS_Coral_Reefs_in_Mozambique.pdf

Elmhirst, T., S. R. Connolly, and T. P. Hughes. 2009. Connectivity, regime shifts and the resilience of coral reefs. Coral Reefs 28:949-957.

Greiner, A., E. S. Darling, M. -J. Fortin, and M. Krkošek. 2022. "The combined effects of dispersal and herbivores on stable states in coral reefs." Theoretical Ecology 15:321-335.

Greiner, A., Andrello, M. 2025. ArielGreiner/Fiji_StabilityConnectivitySimulation: Associated with Ecological Applications Publication (v.1). Zenodo. https://doi.org/10.5281/zenodo.17340984

Mumby, P. J., A. Hastings, and H. J. Edwards. 2007. "Thresholds and the resilience of Caribbean coral reefs." Nature 450:98.

Wildlife Conservation Society (WCS). 2022. "Launching a Decade of Action for Coral Reefs."
