# Supplementary material for: Dispersal can spread management benefits: Insights from a modeled Fijian coral reef network
Source: Ecol Appl. 2025 Dec 8;35(8):e70156. doi: 10.1002/eap.70156 (PMC12683702; doi:10.1002/eap.70156)
Supplement: Supplementary file 9 — Appendix S9. [file EAP-35-e70156-s005.pdf]

Title: Dispersal can spread management benefits: Insights from a modeled Fijian coral reef network

Journal Name: Ecological Applications

Authors: Ariel Greiner, Marco Andrello, Martin Krkošek, Marie-Josée Fortin, Yashika Nand, Stacy D. Jupiter, Sangeeta Mangubhai, Amelia Wenger, Emily S. Darling

Appendix S9: Simulating Water Quality Management at Only a Subset of Reefs

In order to directly compare the impact of water quality management with the impact of expanding the fishery closures on the coral cover of the 75 reef network, we had to simulate the result of only improving the water quality at the same subset of reefs that were newly included in fishery closure regions (under M1-2km). We simulated these scattered water quality management interventions at the 10% and 25% levels (M2-10% scat, M2-25% scat; see Figure S1). We also simulated these two scattered water quality interventions in combination with M1-2km (M3-2km + 10% scat and M3-2km + 25% scat, respectively; see Figure S1). Ultimately, we found that the scattered water quality management interventions were less effective than their non-scattered counterparts (M2-10%, M2-25%, M3-2km + 10%, M3-2km + 25%) at increasing coral cover. The scattered water quality management interventions were also less effective at increasing coral cover than the expanding fishery closure management intervention that was performed on the same reefs (M1-2km), indicating that improving grazing rates on those reefs is more effective than improving the water quality for increasing coral cover.

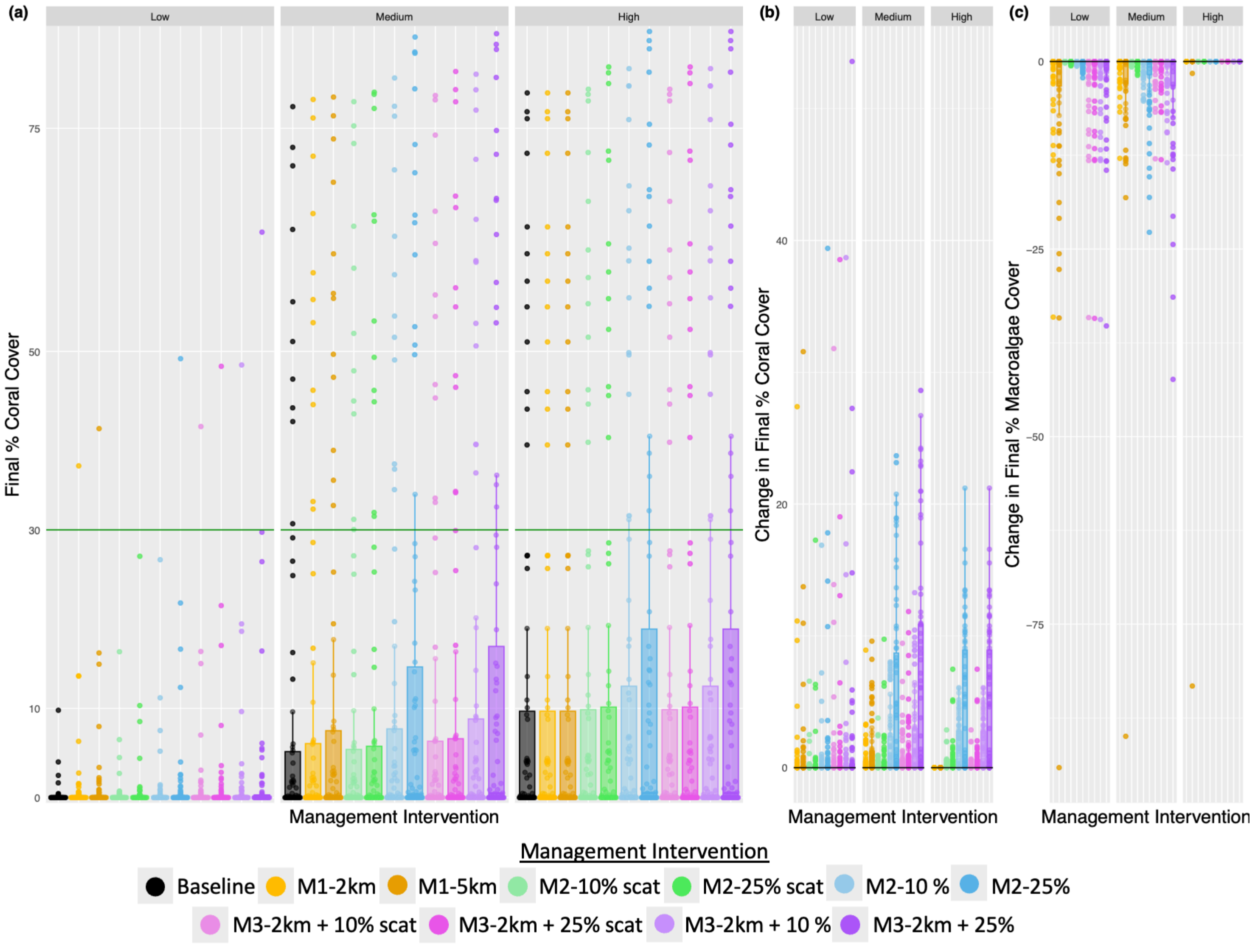

Figure S1. Effects of all the Management Interventions Including Scattered Water Management Interventions - a-c show the effect of the management intervention on the final coral cover of each reef, while each panel shows the effect of the management under each grazing scenario. (a) Final percent coral cover in each reef, with a green line at 30% indicating a healthy reef (Birrell et al., 2020; WCS 2022). (b) Difference in the percent coral cover in each reef between each management intervention and the baseline simulation, the black line at 0 indicates the reefs that went through no change in percent coral cover. (c) Difference in the percent macroalgal cover in each reef between each management intervention and the baseline simulation, the black line at 0 indicates the reefs that went through no change in percent macroalgal cover. 'Baseline' represents the baseline simulations with no modeled management interventions. In (a-c), each point represents the final % coral cover of a particular reef and box plots showing the inter-quartile range of the values are placed behind the points to indicate spread; in (a) the points are jittered along the x-axis to make it easier to distinguish individual points.

References

Birrell, C. L., E. Sola, R. H. Bennett, D. van Beuningen, H. M. Costa, J. J. Sitoe, N. Sidat, S. Fernando, E.S. Darling, N.A. Muthiga and T. R. McClanahan. 2020. "A summary of WCS knowledge of the state of coral reefs in Mozambique." Wildlife Conservation Society, Maputo, Mozambique. https://biblioteca.biofund.org.mz/wp-content/uploads/2021/03/1616752045-2020_WCS_Coral_Reefs_in_Mozambique.pdf

Wildlife Conservation Society (WCS). 2022. "Launching a Decade of Action for Coral Reefs." https://cdn.wcs.org/2021/04/21/99xudme990_4.16.21_English_CBD_Rec_2_Pager.pdf?_gl=1*1f71558*_ga*MTk4MTYyMzY4Ni4xNjc0ODM0MjI1*_ga_BTX9HXMYSX*MTY4MDE5Nzk2MC4xNi4wLjE2ODAxOTc5NjAuNjAuMC4w
