# Supplementary material for: Dispersal can spread management benefits: Insights from a modeled Fijian coral reef network
Source: Ecol Appl. 2025 Dec 8;35(8):e70156. doi: 10.1002/eap.70156 (PMC12683702; doi:10.1002/eap.70156)
Supplement: Supplementary file 10 — Appendix S10. [file EAP-35-e70156-s004.pdf]

Title: Dispersal can spread management benefits: Insights from a modeled Fijian coral reef network

Journal Name: Ecological Applications

Authors: Ariel Greiner, Marco Andrello, Martin Krkošek, Marie-Josée Fortin, Yashika Nand, Stacy D. Jupiter, Sangeeta Mangubhai, Amelia Wenger, Emily S. Darling

Appendix S10: Most Abundant Herbivores observed in WCS Fiji Survey Data

Out of the top 10 most abundant herbivores reported in Fiji by WCS Fiji since 2009, four of them are reported to consume macroalgae (*Zebrasoma scopas*, *Siganus doliatus, Naso lituratus, Siganus uspi*) and the rest are reported to consume various other types of algae, with reported total abundances of n = 5456 for the macroalgae consumers and n = 9045 for the consumers of other algae. This is in line with the findings of Bonaldo et al. (2017) and indicates that there may be more grazing of other algae than macroalgae, overall. However, this still does not clarify whether the consumption rate of the two types of benthos is not indiscriminate on the reefs that were included in this study and during the specified time period (2017-2020).

Table S1: *Top 10 Most Abundant Herbivores Observed in Fiji by WCS Fiji* - This table describes the top 10 most abundant herbivores observed in Fiji by WCS Fiji. This table summarizes data from WCS Fiji from 2009 - 2025 and includes data from reef sites included in this study, among others. The 'Food Items' column lists the reported food items, based on internal data from WCS Fiji (from 2009) and from fishbase.se (Accessed on July 26, 2025; Froese & Pauly, 2025).

Fish Taxon	n	Food Items
Chlorurus bleekeri	2900	benthic algae/weeds
Scarus schlegeli	2321	benthic algae/weeds, zoobenthos, zooplankton
Zebrasoma scopas	2108	Macroalgae, filamenteous algae, benthic algae, other phytoplankton
Siganus doliatus	1375	benthic algae, macroalgae*
Naso lituratus	1067	Macroalgae, zooplankton
Chlorurus microrhinos	1058	benthic algae/weeds
Scarus ghobban	1056	benthic algae/weeds, detritus
Siganus uspi	906	Macroalgae*, benthic algae/weeds, zooplankton
Acanthurus nigrofuscus	860	Benthic algae/weeds, filamenteous algae, phytoplankton, blue-green algae
Centropyge bicolor	850	Benthic algae/weeds, coral polyps, zoobenthos, tunicates, sponges

*Listed as consuming 'seaweed' or 'benthic seaweed' on fishbase.se, confirmed that this was indeed referring to macroalgae consumption by checking Woodland (1997).

References

Bonaldo, R. M., M. M. Pires, P. R. Guimaraes, A. S. Hoey, and M. E. Hay. 2017. "Small marine protected areas in Fiji provide refuge for reef fish assemblages, feeding groups, and corals." PloS one 12:e0170638.

Froese, R. and D. Pauly. Editors. 2025. FishBase. World Wide Web electronic publication. www.fishbase.org, (04/2025)

Woodland, D., 1997. Siganidae. Rabbitfishes (spinefoots). p. 3627-3650. In K.E. Carpenter and V. H. Niem (eds.) FAO Identification Guide for Fishery Purposes. The Western Central Pacific. 837 p.
